# Cinchona‐Based Hydrogen‐Bond Donor Organocatalyst Metal Complexes: Asymmetric Catalysis and Structure Determination

**DOI:** 10.1002/open.202300180

**Published:** 2024-01-08

**Authors:** Sándor Nagy, Dóra Richter, Gyula Dargó, Balázs Orbán, Gergő Gémes, Tibor Höltzl, Zsófia Garádi, Zsuzsanna Fehér, József Kupai

**Affiliations:** ^1^ Department of Organic Chemistry and Technology Budapest University of Technology and Economics Műegyetem rkp. 3 1111 Budapest Hungary; ^2^ Euroapi Hungary Kft. Tó utca 1–5 1045 Budapest Hungary; ^3^ ELKH-BME Computation Driven Chemistry Research Group Department of Inorganic and Analytical Chemistry Budapest University of Technology and Economics Műegyetem rkp. 3 1111 Budapest Hungary; ^4^ Furukawa Electric Institute of Technology Késmárk utca 28/A 1157 Budapest Hungary; ^5^ Department of Pharmacognosy Semmelweis University Üllői út. 26 1085 Budapes Hungary

**Keywords:** metal complex, cinchona, hydrogen-bond donor, asymmetric catalysis, structure elucidation

## Abstract

In this study, we describe the synthesis of cinchona (thio)squaramide and a novel cinchona thiourea organocatalyst. These catalysts were employed in pharmaceutically relevant catalytic asymmetric reactions, such as Michael, Friedel–Crafts, and A^3^ coupling reactions, in combination with Ag(I), Cu(II), and Ni(II) salts. We identified several organocatalyst‐metal salt combinations that led to a significant increase in both yield and enantioselectivity. To gain insight into the active catalyst species, we prepared organocatalyst‐metal complexes and characterized them using HRMS, NMR spectroscopy, and quantum chemical calculations (B3LYP‐D4/def2‐TZVP), which allowed us to establish a structure‐activity relationship.

## Graphical abstract

Three different cinchona‐based organocatalysts containing different hydrogen‐bond donor moieties worked together with inorganic transition metal salts to catalyze pharmaceutically relevant asymmetric reactions. They were also studied experimentally and quantum chemically, providing insight into the complex structures of these catalytically active species. In many cases, the application of these catalysts led to significant increases in both yield and enantioselectivity.

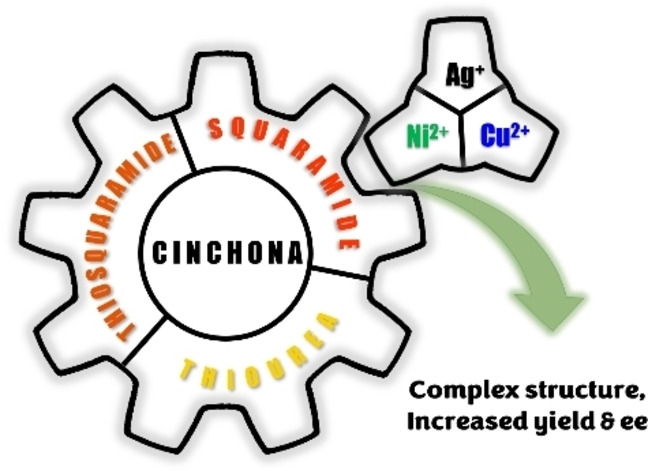

## Introduction

In contrast to historical accomplishments, when chiral molecules were synthesized using asymmetric reagents obtained from the chiral pool, asymmetric synthesis provides a more efficient way to produce enantiopure products. Applying asymmetric catalysts, enantioselective reactions can be carried out, avoiding the resolution of a racemic product. This can be beneficial from both environmental and economic points of view. Since the 1960s,[^1^, ^2^, ^3^, ^4^] asymmetric metal catalysis has provided the only solution to prepare enantiopure products until the first applications of enzymes and organocatalysts. From the early 2000s, the first successful applications of asymmetric organocatalysis have been published,[^5^, ^6^, ^7^, ^8^, ^9^] and nowadays, their application[^10^, ^11^] in asymmetric synthesis is as widespread as that of transition metal‐based catalysts with chiral ligands.[^12^, ^13^, ^14^]

Within organocatalysis, bifunctional catalysts have become comprehensive. This bifunctionality means the capability to activate the nucleophile and electrophile reactants simultaneously.[^15^, ^16^] Cinchona moiety is one of the privileged chiral skeletons in asymmetric organocatalysis.^[17]^ The tertiary amino group in the quinuclidine ring gives basic character to the molecule, hence this can activate/fix nucleophiles or electrophiles, and possessing a chiral skeleton, the cinchona unit is responsible for chiral induction also. Connecting the cinchona skeleton to a double hydrogen‐bond donor moiety [thiourea, (thio)squaramide], which can fix the corresponding substrate by forming a strong double hydrogen bond, extends their applicability.[^18^, ^19^, ^20^, ^21^, ^22^] Their outstanding versatility is proven by their successful applications in asymmetric reactions with high yields and enantiomeric excess values. Such reactions include but are not limited to, aza‐Henry,^[23]^ Mannich,^[24]^ (aza‐)Michael,[^25^, ^26^] and selenosulfonylation^[27]^ reactions.

Thiosquaramides,^[28]^ sulfur analogs of squaramides were first predicted computationally as potentially more effective hydrogen‐bond donating organocatalysts.^[29]^ Recently, Rawal and co‐workers, and other research groups synthesized thiosquaramide catalysts and applied them in Michael addition, conjugate,^[30]^ and aza‐Diels–Alder reactions.[^31^, ^32^, ^33^, ^34^, ^35^] These bifunctional thiosquaramides are a promising new class of catalysts, which possess increased aromaticity, higher acidity, and greater solubility in non‐polar solvents compared to those of squaramides and thioureas.

Transition metals combined with organocatalysts can merge their superior qualities to promote reaction systems that can lead to new transformations.[^36^, ^37^, ^38^, ^39^, ^40^, ^41^, ^42^, ^43^, ^44^, ^45^] This way, multicomponent or multistep reactions could take place that cannot be realized without their combined application.[^46^, ^47^, ^48^, ^49^, ^50^, ^51^, ^52^, ^53^, ^54^, ^55^, ^56^, ^57^]

Consequently, based on our previous work, procuring the corresponding knowledge in the field of organocatalysis, we aimed to combine cinchona (thio)squaramide and thiourea organocatalysts with copper(II), nickel(II), and silver(I) salts in reactions that are relevant in pharmaceutical aspects, such as Michael addition, Friedel–Crafts reaction and A^3^ coupling reaction. Furthermore, we have found explanations for the formation and activity of the prepared organocatalyst‐metal complexes via the applied quantum chemical calculations linked to analytical techniques.

## Results and Discussion

### Preparation of the organocatalyst units

**Scheme 1:**
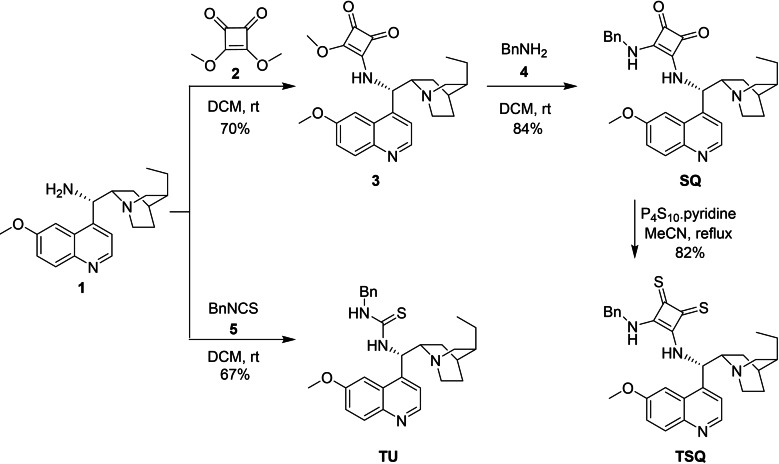
Synthetic routes of organocatalyst representatives (**SQ**, **TSQ**, **TU**).

The key intermediate cinchona amine (**1**) was prepared starting from quinine using published methods.^[58]^ Cinchona amine (**1**) was then converted to the corresponding cinchona half squaramide (**3**) by reacting it with dimethyl squarate (**2**).^[34]^ The cinchona squaramide (**SQ**) organocatalyst was obtained in the reaction of cinchona half squaramide (**3**) and benzylamine (**4**). By the thionation of cinchona squaramide (**SQ**) with phosphorus pentasulfide pyridine complex, cinchona thiosquaramide (**TSQ**) was gained.^[59]^ The third organocatalyst representative, cinchona thiourea (**TU**), was prepared in the reaction of cinchona amine (**1**) and benzyl isothiocyanate (**5**). For the synthetic route, see Scheme 1.

### Application of organocatalysts combined with metal salts

The metal cations [Ag(I), Ni(II), Cu(II)] thanks to their Lewis acidic character, coordinate the Lewis basic atom of the corresponding electrophiles, reducing their LUMO energy level. As a result, the energy of the electrophile approaches the HOMO energy of the attacking nucleophile. This process reduces the activation energy barrier. This phenomenon also occurs in organocatalysis, as well. In addition, the chiral skeleton of the organocatalyst facilitates chiral induction, which results in increased selectivity towards the corresponding enantiomer.

#### Michael addition

Organocatalysts **SQ**, **TSQ**, and **TU** combined with Cu(OAc)_2_, Ni(OAc)_2_ and AgOAc were firstly used in Michael addition, in a known typically organocatalytic test reaction. By way of comparison, Michael addition was carried out without the addition of metal salts, and without organocatalyst, as well. When only metal salts were added to the reaction, product was not found in the reaction mixture, only the starting materials. The results can be seen in Table 1.

**Table 1:** Test of organocatalysts **SQ**, **TSQ** and **TU** combined with Cu(II), Ni(II) and Ag(I) acetates in Michael reaction using *trans*‐β‐nitrostyrene (**6**) and pentane‐2,4‐dione (**7**).^[a]^

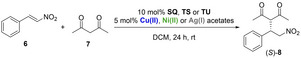				
				
Entry	Organocatalyst	Metal acetate	Yield [%]^[b]^	ee [%]^[c]^
1	**SQ**	**Cu(II)**	86	99
2	**SQ**	**Ni(II)**	83	>99
3	**SQ**	**Ag(I)**	>99	>99
4	**SQ**	–	90	88
5	**TSQ**	**Cu(II)**	99	99
6	**TSQ**	**Ni(II)**	99	99
7	**TSQ**	**Ag(I)**	53	60
8	**TSQ**	–	92	96
9	**TU**	**Cu(II)**	86	84
10	**TU**	**Ni(II)**	86	89
11	**TU**	**Ag(I)**	79	47
12	**TU**	–	74	92

[a] Reaction conditions: 0.005 mmol of Cu(II), Ni(II) or Ag(I) acetate was added to the solution of 0.010 mmol **SQ**, **TSQ** or **TU** in 0.5 mL of DCM. To this solution *trans*‐β‐nitrostyrene (**6**, 0.100 mmol) then pentane‐2,4‐dione (**7**, 0.190 mmol) were added. The resulting mixture was stirred at room temperature for 24 hours. [b] Isolated yields. [c] Determined by chiral HPLC (*S* enantiomer).

Based on the literature, double hydrogen bond organocatalysts, such as thiosquaramides, could form a 2 : 1 complex with metal salts.[^44^, ^60^, ^61^] Thus, the organocatalyst:metal ratio was set to 2 : 1.

In the case of squaramide (**SQ**), AgOAc facilitated the Michael addition by giving a higher yield (Entry 3, Table 1) compared with the purely organocatalytic reaction (Entry 4, Table 1). Meanwhile, in the case of Cu(II) and Ni(II) salts, the yields were lower (Entries 1 and 2, Table 1). The enantiomeric excess values were significantly higher in the presence of metal salts.

Regarding the yields, the opposite effect was observed in case of thiosquaramide (**TSQ**), its combination with AgOAc gave the lowest yield (Entry 7, Table 1). With Cu(OAc)_2_ and Ni(OAc)_2_, the yields were higher (Entries 5 and 6, Table 1). The same tendency was observed in the enantiomeric excess values: those were higher in any cases except in the presence of AgOAc.

The metal salts combined with **TU** showed an increase in yields but lowered the *ee* compared to the values with the application of pure organocatalyst (**TU**, Entry 12, Table 1). As a summary of Michael addition reactions, organocatalysts kept their activity in the presence of metal salts, moreover, in their presence, the yield and *ee* increased in numerous cases.

#### Friedel–Crafts reaction

Friedel–Crafts reaction is acknowledged as a typically metal‐(Lewis acid) catalyzed reaction, and its asymmetric catalysis is known, as well.^[62]^ But as in the case of Michael addition, several asymmetric organocatalytic examples of Friedel–Crafts reaction are known.[^63^, ^64^, ^65^] Therefore, it is such a test reaction, in which both the organocatalyst and metal cation moieties could be involved during the catalytic cycle.

As starting materials, indole (**9**) and ethyl trifluoropyruvate (**10**) were chosen, the ratio of the organocatalyst:metal salt was set to 2 : 1. By the application of squaramide (**SQ**) in a combination of any metal acetate, the reaction was nearly complete in any cases (Entries 1–4, Table 2). However, practically racemic product was gained using squaramide (**SQ**) with AgOAc (Entry 3, Table 2). In other cases, the *ee* fell in the region of 19–25 %, however, the presence of Cu(OAc)_2_ gave the *S* enantiomer (Entry 1, Table 2), otherwise the *R* enantiomer was in excess (Entries 2–4, Table 2).

**Table 2:** Test of organocatalysts **SQ**, **TSQ** and **TU** combined with Cu(II), Ni(II) and Ag(I) acetates in Friedel–Crafts reaction using indole (**9**) and ethyl trifluoropyruvate (**10**).^[a]^

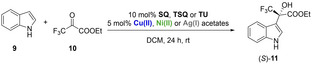				
				
Entry	Organocatalyst	Metal acetate	Yield [%]^[b]^	ee [%]^[c]^
1	**SQ**	**Cu(II)**	94	19
2	**SQ**	**Ni(II)**	>99	22^[d]^
3	**SQ**	**Ag(I)**	>99	3^[d]^
4	**SQ**	–	>99	25^[d]^
5	**TSQ**	**Cu(II)**	88	41
6	**TSQ**	**Ni(II)**	93	72
7	**TSQ**	**Ag(I)**	87	55
8	**TSQ**	–	99	13
9	**TU**	**Cu(II)**	90	36
10	**TU**	**Ni(II)**	99	44
11	**TU**	**Ag(I)**	60	35
12	**TU**	–	81	42
13	–	**Cu(II)**	87	0
14	–	**Ni(II)**	98	0
15	–	**Ag(I)**	92	0

[a] Reaction conditions: 0.006 mmol of Cu(II), Ni(II) or Ag(I) acetate was added to the solution of 0.012 mmol **SQ**, **TSQ** or **TU** in 1.5 mL of DCM. To this solution indole (**9**, 0.120 mmol) then ethyl trifluoropyruvate (**10**, 0.132 mmol) were added. the resulting mixture was stirred at room temperature for 24 hours. [b] Isolated yields. [c] Determined by chiral HPLC (*S* enantiomer). [d] *R* enantiomer.

Using thiosquaramide (**TSQ**), the yields were above 85 % (Entries 5–9, Table 2). The best yield was achieved without the addition of metal salts, but in this case the enantioselectivity was poor (Entry 8, Table 2). The best result (72 % *ee*, 93 % yield) was observed when thiosquaramide was combined with nickel(II) acetate (Entry 6, Table 2). Thiourea (**TU**) showed a medium enantioselectivity in any combinations (35–44 %), with a good to high yield, except in the presence of AgOAc, when the yield was only 60 % (Entry 11, Table 2).

As it could be predicted, metal salts in the absence of organocatalysts were capable of catalyzing Friedel–Crafts reaction giving racemic products (Entries 13–15, Table 2). In most cases, presumably a competing reaction occurred between organocatalytic and metal units considering the high yields given by pure metal catalysis.

On the other hand, the cooperation of organocatalysts with metal salts can also be observed. By the application of pure **TSQ**, 13 % *ee* and 99 % yield (Entry 8, Table 2), with only Ni(OAc)_2_ 98 % yield and racemic product were observed (Entry 14, Table 2). But their combination is favorable: 72 % enantiomeric excess with a high 93 % yield was achieved (Entry 6, Table 2).

#### A^3^‐coupling reaction

The A^3^‐coupling reaction is considered a typically metal‐catalyzed (mainly by Cu salts) reaction. Its Cu(OTf)_2_ catalyzed asymmetric reactions with PyBOX ligands are already known.[^66^, ^67^] One of our goals was to investigate the catalytic effect of bifunctional cinchona‐based organocatalysts combined with metal salts in such a metal‐catalyzed reaction, to see whether these organocatalysts acted as ligands only or catalysts as well.

As starting materials, aniline (**12**), benzaldehyde (**13**), and phenylacetylene (**14**) were chosen. The same, previously used catalytic systems were applied in DCM, but in the A^3^ reaction, no or traces of product was detected in the reaction mixtures. Based on the previously mentioned literature,[^66^, ^67^] we applied Cu(OTf)_2_ in the reactions instead of Cu(OAc)_2_. However, this change did not influence the outcomes. Presumably, the ratio of the organocatalytic moiety to Cu(OTf)_2_, solvent, or addition sequence of components have effects on the catalytic activity of Cu(II) cation. Hence,

- five different types of solvents were tested,
- the organocatalyst‐metal salt ratio and addition sequence of components were changed,
- additives were applied.

Product was not detected by using thiosquaramide (**TSQ**) or organocatalyst without metal salts, and in most cases, the same results can be established in MeCN or in MeOH. The best results were obtained using toluene and THF (Table 3).

**Table 3:** Test of organocatalysts **SQ** and **TU** combined with Cu(II), Ni(II) and Ag(I) acetates in Friedel–Crafts reaction using aniline (**12**), benzaldehyde (**13**) and phenylacetylene (**14**).^[a]^

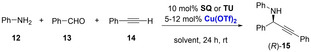					
					
Entry	Organocat.	Orgcat. : Cu(OTf)_2_ ratio	Solvent	Yield [%]^[b]^	ee [%]^[c]^
1	**SQ**	1 : 1	toluene	17	22
2	**SQ**	1 : 1.2	toluene	6	19
3	**SQ**	1.2 : 1	toluene	34	23
4	**SQ**	1 : 1	THF	29	2
5	**SQ**	1 : 1.2	THF	17	3
6	**SQ**	1.2 : 1	THF	7	rac.
7	**TU**	1 : 1	toluene	32	rac.
8	**TU**	1 : 1.2	toluene	26	rac.
9	**TU**	1.2 : 1	toluene	18	5
10	**TU**	1 : 1	THF	21	13^d^
11	**TU**	1 : 1.2	THF	22	14^d^
12	**TU**	1.2 : 1	THF	33	15^d^

[a] Reaction conditions: 0.05–0.012 mmol Cu(II), Ni(II) or Ag(I) acetate was added to the solution of 0.010 mmol of **SQ**, **TSQ** or **TU** in 0.5 mL of DCM. To this solution aniline (**12**, 0.121 mmol) and benzaldehyde (**13**, 0.100 mmol) were added. Next, phenylacetylene (**14**, 0.146 mmol) was added. The resulting mixture was stirred at room temperature for 24 hours. [b] Isolated yields. [c] Determined by chiral HPLC (*R* enantiomer). [d] *S* enantiomer.

It can be thought that the applied organocatalytic moieties acted as catalyst poison (especially **TU**), as in many cases no product was detected. This phenomenon, the poor yield and *ee* in the A^3^ reaction, can be explained by the strong interaction between the organocatalyst and Cu(II). Organocatalysts may be coordinated too strongly to Cu(II) cation which led to poor yield and enantiomeric excess values. It is important to underline that reaction took place only when organocatalyst:Cu(OTf)_2_ ratio was below 2 : 1. Copper(II) cation is bound strongly in solution by organocatalyst, during which Cu(II) was hidden by the organocatalyst skeleton leading to the reduction of catalytic activity in the A^3^ coupling reaction.

By applying **SQ**, the best result was obtained in toluene with a 1.2 : 1 organocatalyst:Cu(OTf)_2_ ratio (34 % yield, 23 % *ee*, Entry 3, Table 3). A similar yield (33 %) was gained when **TU** was applied with the same catalyst ratio in THF, but that catalyst‐solvent system gave the opposite (*S*) enantiomer in excess (15 % *ee*). These reactions were also carried out at 0 °C, and in the presence of acetic acid additive, but the results were not significantly different.

After the best results were gained in ethereal and hydrocarbon‐type solvents, the reactions were also carried out in MTBE, anisole, and mesitylene, but these reactions showed lower yields and *ee* values (see Table S3 Supporting Information).

Further investigations on the low catalytic activity of these complexes in the A^3^‐coupling reaction can be seen in the quantum chemical calculation section.

#### Preparation and analytical measurements of organocatalyst‐metal complexes

The organocatalyst‐metal complexes were active in a typical organocatalytic reaction (Michael addition), and in a reaction that can be catalyzed by organocatalyst and metal salts, too (Friedel–Crafts reaction). Meanwhile, in a copper‐catalyzed reaction (A^3^ reaction), these complexes were not as active as the metal salt itself, especially **TSQ** was the most effective in Friedel–Crafts reaction regarding enantioselectivity (Entry 6, Table 2). For better understanding, in parallel with quantum chemical calculations, we prepared the catalyst complexes to investigate their structure.

**Figure 1:**
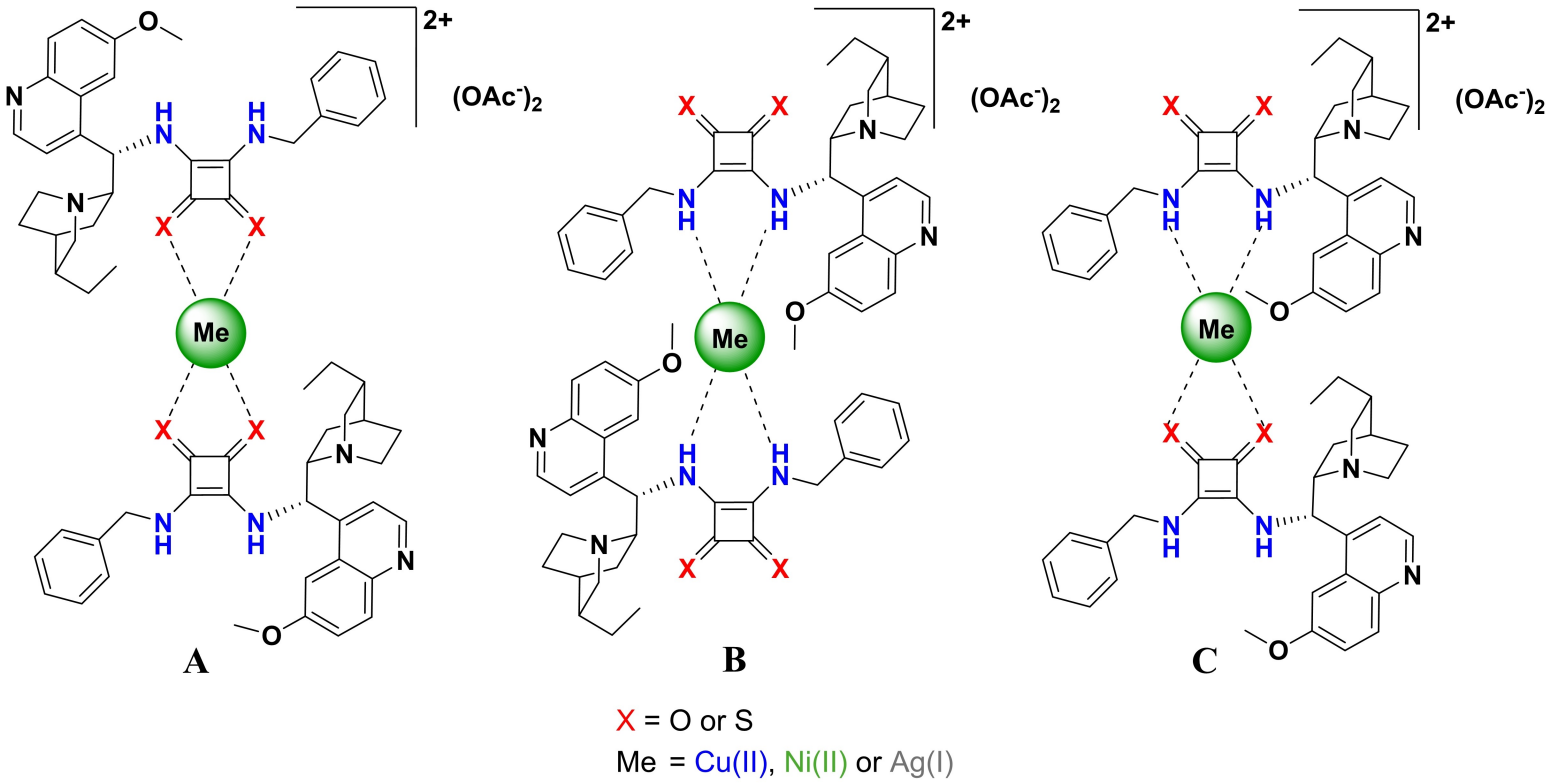
Three possible structures of **SQ**‐ and **TSQ**‐metal complexes.

According to Busch and co‐workers, in the case of thiosquaramides, the coordinative bonds are formed between the thiocarbonyl group and the transition metals[^60^, ^61^] (Figure 1A). In contrast, Santos and co‐workers claim that these bonds are formed only between the nitrogen atoms of the thiosquaramides and the transition metals (Figure 1B).^[44]^ Derived from these statements, a third possible structure (Figure 1C) can be drawn up combined with results in the literature that examined solid‐state squaramides.[^68^, ^69^, ^70^, ^71^]

**Figure 2:**
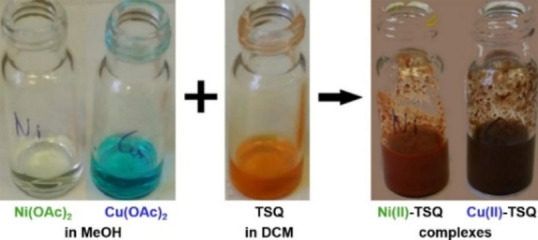
Color change preparing **TSQ**‐Ni(II) and Cu(II) complexes.

To understand the behavior of these complexes, they were prepared by adding **SQ**, **TSQ**, or **TU** dissolved in DCM to a solution of the corresponding metal acetate in a ratio of 2 : 1, then the solvent was evaporated. Significant color change was observed in the reaction of **TSQ** and Ni(II) or Cu(II) acetates (Figure 2).

During the analytical measurements, the molecule ion of organocatalyst:metal=2 : 1 complexes was detected by HRMS with high accuracy (see Table 4 and Figure S13–S24 in Supporting Information). The complex of Ag(I) with **SQ** or **TU**, and Ni(II) with **SQ** was not detected in HRMS‐ESI.

**Table 4:** Test of organocatalysts **SQ** and **TU** combined with Cu(II), Ni(II) and Ag(I) acetates in Friedel–Crafts reaction using aniline (**12**), benzaldehyde (**13**) and phenylacetylene (**14**).^[a]^

Entry	Complex	Observed specimen	HRMS‐ESI		Difference (ppm)	Appearance
			Calcd.	Found		
1	2SQ ⋅ AgOAc	Complex was not detected in HRMS			–	Grey powder
2	2TSQ ⋅ AgOAc	M^+^ [2TS ⋅ Ag(I)]	1191.3399	1191.3420	1.8	Yellow powder
3	2TU ⋅ AgOAc	Complex was not detected in HRMS			–	Black powder
4	2SQ ⋅ Cu(OAc)_2_	M−H [2SQ ⋅ Cu(II)]	1082.4480	1082.4498	1.7	Dark brown powder
5	2TSQ ⋅ Cu(OAc)_2_	M^+^ [2TS ⋅ Cu(II) ]	1147.3644	1147.3662	1.6	Dark brown powder
6	2TU ⋅ Cu(OAc)_2_	M−H^+^ [2TU ⋅ Cu(II) ]	1010.4124	1010.4114	−1.0	Dark green powder
7	2SQ ⋅ Ni(OAc)_2_	Complex was not detected in HRMS			–	Light green powder
8	2TSQ ⋅ Ni(OAc)_2_	M−H [2TS ⋅ Ni(II) ]	1141.3623	1141.3621	−0.2	Dark red powder
9	2TU ⋅ Ni(OAc)_2_	M−H^+^ [2TU ⋅ Ni(II) ]	1005.4182	1005.4169	−1.3	Off‐white powder

[a] The observed specimen and HRMS measurement results of organocatalyst–metal complexes, and their appearance.

**Figure 3:**
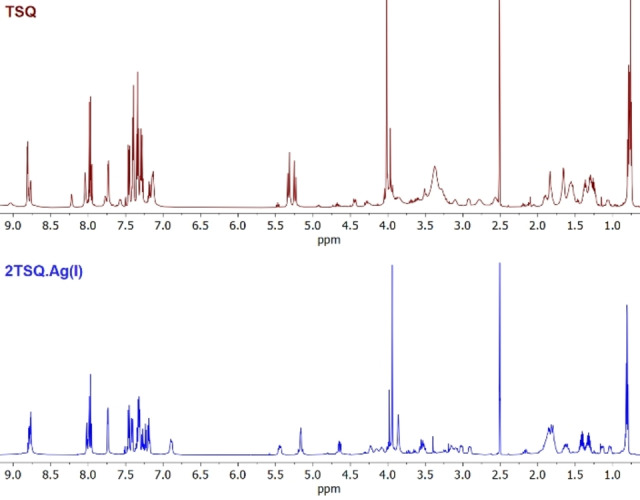
Stacked ^1^H‐NMR spectra of **TSQ** and its Ag(I) complex.

As Ni(II) and Cu(II) have paramagnetic properties, only **TSQ**‐Ag(I) complex gave evaluable NMR spectra (see Figure 3). Noticeable differences can be seen in the aliphatic region (2.5–5.5) between thiosquaramide (**TSQ**) and its Ag(I) complex. The shifts of NMR signals show that the complex unequivocally differs from **TSQ**. Based on the lack of duplicated proton signals in the ^1^H NMR, the spectrum shows that it is a unitary, single material implying that the complex must have symmetry element(s). For ^13^C, COSY, HSQC, HMBC, TOCSY, ROESY spectra, see ESI Figures S13–S18 in SI.

Regrettably, growth of single crystal was unsuccessful, hence; investigation by its X‐ray diffraction was not possible. Therefore, we applied quantum chemical calculations to further study the structures of organocatalyst‐metal complexes.

#### Quantum chemical calculations

**Figure 4:**
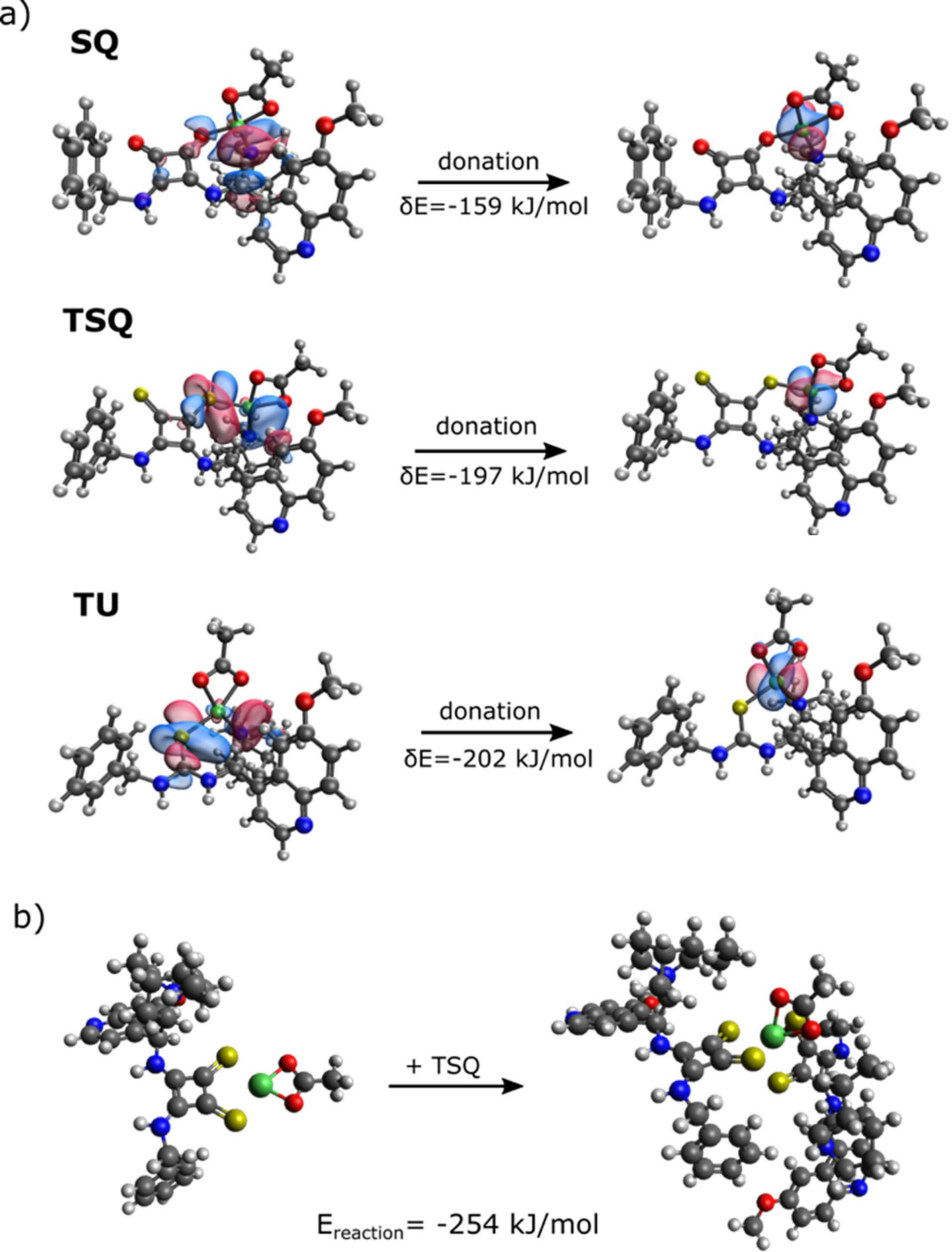
(a) Optimized structure of L−Ni(II)‐ac complexes (L=SQ, TSQ, TU) indicating the donor‐acceptor orbitals and the energy change of the interaction between the ligand and Ni(II)‐ac. (b) Structure and reaction energy of Ni complex with two TSQ ligands formed with the coordination of a TSQ ligand to Ni complex with one ligand. Yellow, red, blue, grey and white colors indicate sulfur, oxygen, nitrogen, carbon and hydrogen atoms, respectively.

Quantum chemical computations were performed using the Q‐Chem 6.0 program package^[72]^ to study the geometric and electronic structure of the nickel complexes and to further understand the relationship of the complex formation and their reactivities (see chapter 6 in Supporting Information for the details of the computations). The quantum chemical computations were performed using the B3LYP‐D3(0)/def2‐SVP method. First, different coordination modes of one SQ, TSQ and TU ligand with Ni(II)‐ac were investigated (Figure 4a and see also Chapter 6 in Supporting Information with more details). The computations showed that in the most stable structure the ligand coordinates to the nitrogen atom of the quinuclidine group (and at the same time also one of the O/S atoms of the SQ/TSQ/TU unit). The energy decomposition analysis^[73]^ showed that the strong stabilization of the nitrogen‐nickel interactions is due to the polarization and charge transfer involving the nitrogen atom in the quinuclidine group. This can be attributed to the larger overlap of the atomic orbitals of Ni and N than those of the Ni and O/S. Figure 4a shows that the charge transfer originates from the electron donation of ligand p system to one of the formally unoccupied *d* orbitals of Ni. Furthermore, it was found that the coordination with N atoms of the TSQ and TU unit is the least stable while in the case of SQ ligand it is slightly more favorable than coordinating with the oxygen atoms of the squaramide ring (differing by 16 kJ mol^−1^ in energy).

For TSQ, the structure and formation of the complex with two ligands were also examined. The most stable structures were observed when the Ni binds to the S atoms, while binding to the N atoms of the **TSQ** units makes the structure significantly less stable (Figure 4a). This can be explained by the breaking of the delocalized π system around the nitrogen atoms due to the presence of the Ni−N bond. However, in the case of complex with Ni−S binding, the coordination of two ligands is more favorable (−254 kJ mol^−1^) than that of only one ligand (Figure 4b). This suggests that in solution the Ni coordination to two ligands is preferred. It has to be noted that the optimized structure with two ligands shown in Figure 4b might not be the most stable coordination mode due to the complexity of the structure with several possible binding groups to the central nickel atom. However, the facile formation clearly indicates that the complex formation with two ligands is strongly preferred.

As it has been described above, coordination of two ligands to the Ni atom can reduce its catalytic activity in A^3^ coupling reaction. To prevent the deactivation of metal catalyst, smaller organocatalyst‐metal salt ratio may promote the complex formation with only one ligand and even the presence of free metal salt in the system.

Thus, the formation of the complexes with one ligand was also examined in six different solvents: DMSO, MeCN, MeOH, DCM, THF and toluene. Based on the calculated formation energies the order of stability of complex formation is MeOH>MeCN>DMSO>DCM>THF>toluene. The formation of less stable complexes implies that metal catalysts and ligands (organocatalysts) are present in the system in their catalytically active forms, as explained above. This also prevents the formation of complex with two ligands which would reduce the catalytic activity in A^3^ coupling reaction. Thus, these computations are in line with the experimentally observed best results for A^3^ coupling reaction in THF and toluene, which are the solvents with the lowest complex stability while the reaction was not successful in DCM, MeCN and MeOH where the complex is more stable.

## Conclusions

We synthesized cinchona squaramide (**SQ**), thiosquaramide (**TSQ**), and a new thiourea (**TU**) organocatalysts. They were combined with Ag(I), Cu(II), and Ni(II) salts to explore their potential in catalysis. These organocatalyst‐metal salt systems were used in pharmaceutically relevant reactions, specifically Michael addition, Friedel–Crafts, and A^3^ coupling reactions. While the Michael addition is typically organocatalytic, the Friedel–Crafts reaction can be carried out using both organocatalytic and metal‐catalytic methods. The A^3^ coupling reaction, on the other hand, is typically copper‐catalyzed.

We developed metal‐enhanced organocatalytic systems that significantly increased both yield and enantioselectivity, comparing these values to those gained without metal salts. For example, combining AgOAc with cinchona squaramide (**SQ**) resulted in >99 % yield and ee in the Michael addition reaction of *trans*‐β‐nitrostyrene (**6**) and pentane‐2,4‐dione (**7**). This showed a 10 % increase in yield and a 12 % increase in ee compared to using the corresponding organocatalyst (**SQ**) alone. In Friedel–Crafts reactions, the highest enantioselectivity (72 % ee with 93 % yield) was achieved using cinchona thiosquaramide (**TSQ**) in combination with Ni(OAc)_2_, compared to only 13 % ee (with 99 % yield) using **TSQ** without metal salt. In A^3^ coupling reactions, the best yield was 34 % with 23 % ee (**SQ** : Cu(OTf)_2_=1.2 : 1, in toluene).

Applying **TU** in THF, A^3^ reaction resulted in the opposite enantiomer.

To identify the active species in the reactions, we prepared organocatalyst‐metal complexes. Their structures were identified using HRMS, NMR spectroscopy, and quantum chemical calculations (B3LYP‐D3(0)/def2‐SVP). The ratio of organocatalyst‐metal cation was found to be 2 : 1 in these complexes, with the **TSQ** coordinating to the metal ion via sulfur atoms. In the case of **SQ** and **TU**, in the most stable structure, the metal ion coordinated to the nitrogen atom of the quinuclidine group and to one of the O/S atoms of the SQ/TU unit.

## Experimental Section

### General

NMR spectra were recorded at the Directorate of Drug Substance Development, Egis Pharmaceuticals Plc., on a Bruker Avance III HD (at 600 MHz for ^1^H and at 150 MHz for ^13^C NMR spectra) or at the Department of Inorganic & Analytical Chemistry, Budapest University of Technology and Economics, on a Bruker DRX‐500 Avance spectrometer (at 500 MHz for ^1^H and at 125 MHz for ^13^C NMR spectra) or on a Bruker 300 Avance spectrometer (at 300 MHz for ^1^H and at 75 MHz for ^13^C NMR spectra) at temperatures given. The exact mass measurements were performed using a Q‐TOF Premier mass spectrometer (Waters Corporation, 34 Maple St, Milford, MA, USA) in positive electrospray ionization mode. Optical rotations were measured on a Perkin–Elmer 241 polarimeter that was calibrated by measuring the optical rotations of both enantiomers of menthol. Starting materials and solvents were purchased from Merck (Darmstadt, Germany); unless stated otherwise, starting materials and reagents [quinine, dimethyl squarate (**2**), benzyl amine (**4**), phosphorous pentasulfide, pyridine, benzyl isothiocyanate (**5**), *trans*‐β‐nitrostyrene (**6**), pentane‐2,4‐dione (**7**), Cu(OAc)_2_, Ni(OAc)_2_, AgOAc, indole (**9**), ethyl trifluoropyruvate (**10**), aniline (**12**), benzaldehyde (**13**) and phenylacetylene (**14**)] were purchased from Sigma–Aldrich (Saint Louis, MO, USA) and used without purification. The enantiomeric excess (*ee*) values were determined by chiral HPLC on a Perkin Elmer Series 200 instrument. For Michael additions: Phenomenex Lux® 5 μm, Cellulose‐1 column (250×4.6 mm ID, a mixture of hexane:ethanol=85 : 15 as the eluent with a flow rate of 0.8 mL min^−1^, UV detector α=254 nm). For Friedel–Crafts reactions: Phenomenex Lux® 5 μm, Cellulose‐1 column (250×4.6 mm ID, a mixture of water (0.1 % NH_4_OAc):MeCN=40 : 60 as the eluent with a flow rate of 0.8 mL min^−1^, UV detector α=254 nm). For A^3^ reactions: Kromasil® AmyCoat 5 μm, Cellulose‐1 column (250×4.6 mm ID, a mixture of hexane:ethanol=85 : 15 as the eluent with a flow rate of 0.8 mL min^−1^, UV detector α=254 nm). Melting points were taken on a Boetius micro‐melting point apparatus and they were uncorrected. Silica gel 60 F_254_ (Merck) plates were used for TLC. The spots of materials on TLC plates were visualized by UV light at 254 nm or using potassium permanganate as visualizer. Silica gel 60 (70–230 mesh, Merck) was used for column chromatography. Ratios of solvents for the eluents are given in mL.

All computations were carried out using the Q‐Chem 6.0 software package.^[72]^ Density Functional Theory (DFT) computations were performed using the B3LYP functional[^74^, ^75^] def2‐SVP^[76]^ basis set. To consider dispersion effects in the complexes the D3(0) empirical correction^[77]^ was employed. In geometry optimization. Convergence of Self‐consistent field (SCF) energy calculation was set to 10^−8^ Ha while equilibrium structure was obtained once the change in total energy was less than 10^−6^ Ha and gradient was smaller than 3×10^−4^ Ha/Å. The geometries of the complexes were optimized in both singlet and triplet states. For the investigation of the electronic structure and bonds of the complexes Natural Bond Orbital (NBO) and Energy Decomposition Analysis based on Absolutely Localized Molecular Orbitals (ALMO‐EDA) were carried out.^[78]^ To account for solvent effects, the SM12 implicit solvent model was chosen and geometries optimized in gas phase were used.^[79]^

#### 1‐Benzyl‐3‐((1S)‐((1S,2S,4S)‐5‐ethylquinuclidin‐2‐yl)(6‐methoxyquinolin‐4‐yl)methyl)thiourea (TU)

To a solution of cinchona amine (**1**, 1.00 g, 3.08 mmol) in chloroform (10 mL) MgSO_4_ (150 mg) was added to absorb moisture and the mixture was stirred for 5 min. To this suspension, benzyl isothiocyanate (**5**, 459 mg, 0.408 mL, 3.08 mmol) was added slowly, and the mixture was stirred overnight. The reaction mixture was filtered, volatile components were evaporated under reduced pressure. The crude product was purified by column chromatography (silica gel, DCM : methanol:25 % NH_4_OH=10 : 1 : 0.3, (*R*_f_=0.44). **TU** was obtained as an off‐white crystalline solid (977 mg, 67 %). TLC (SiO_2_ TLC; DCM : methanol:25 % NH_4_OH=10 : 1 : 0.3, *R*_f_=0.44); M.p. 107–110 °C; ). ${\left[a\right]}_{20}^{D}$ −154.5 (c 1.00, CHCl_3_); IR *ν*_max_ 3258, 3029, 2953, 2926, 2962, 1621, 1544, 1508, 1475, 1453, 1432, 1368, 1343, 1296, 1260, 1241, 1227, 1029, 854, 698 cm^−1^; ^1^H NMR (500 MHz, CDCl_3_, 25 °C) δ 8.67 (m, 1H), 7.95 (d, *J*=9.5 Hz, 1H), 7.83 (br s, 1H), 7.42 (m, 1H), 7.33 (dd, *J*=9.0 Hz, 1.5 Hz, 1H), 7.13–7.18 (m, 5H), 6.34 (br s, 1H), 4.61 (q, δ_A_: 4.61 and δ_B_: 4.64 ; *J*_AB_=13.3 Hz, 2H), 3.94 (s, 3H), 3.82 (m, 2H), 3.37 (m, 1H), 2.87 (m, 1H), 2.71 (m, 1H), 1.93 (m, 1H), 1.69–1.77 (m, 2H), 1.49 (m, 1H), 1.18–1.27 (m, 3H), 1.08–1.10 (m, 1H), 0.77 (t, *J*=7.5 Hz, 3H) ppm; ^13^C NMR (125 MHz, CDCl_3_, 25 °C) δ 182.4, 158.4, 147.7 (2C), 145.0, 131.9, 128.6 (3C), 127.7 (3C), 127.4, 122.5, 102.1, 56.1, 48.8, 41.9, 35.2, 31.9, 29.7, 26.2, 25.6, 24.7, 23.9, 21.4, 11.5 ppm; HRMS‐ESI+ (m/z): [M+H^+^] calcd. for C_28_H_35_N_4_OS: 475.2532, found: 475.2530. Anal. calc. (%): C, 70.85; H, 7.22; N, 11.80; S, 6.75. Found: C, 70.81; H, 7.26; N, 11.72; S, 6.77.

To the best of our knowledge, the synthesis of **TU** has not been reported.

#### General procedure for Michael addition of pentane‐2,4‐dione (7) to trans‐b–nitrostyrene (6)

To a solution of organocatalyst (0.010 mmol **SQ**, **TSQ** or **TU**) in DCM (0.5 mL) metal acetate (0.005 mmol Cu(OAc)_2_ or Ni(OAc)_2_ or AgOAc) was added. To this solution *trans*‐β‐nitrostyrene (**6**, 0.100 mmol), then pentane‐2,4‐dione (**7**, 0.190 mmol) were added. The resulting mixture was stirred at room temperature for 24 hours. The volatile components were removed under reduced pressure. The crude product was purified by preparative thin layer chromatography on silica gel using hexane:ethyl acetate=2 : 1 mixture (*R*_f_=0.36) as eluent to obtain Michael adduct as pale‐yellow crystals. ^1^H NMR (500 MHz, CDCl_3_, 25 °C) δ 1.93 (s, 3H), 2.28 (s, 3H), 4.21–4.26 (m, 1H), 4.35–4.37 (m, 1H), 4.61–4.63 (m, 2H), 7.11–7.18 (m, 2H), 7.29–7.32 (m, 3H); ^13^C NMR (125 MHz, CDCl_3_, 25 °C) δ 29.7, 30.6, 42.9, 70.9, 78.3, 128.1, 128.7, 129.5, 136.1, 201.1, 201.9 ppm. Yields and enantiomeric excess (*ee*) values can be seen in Table 1. These products had the same spectroscopic data than those of reported (the absolute configuration was determined by the optical rotation of the products).^[26]^ Phenomenex Lux® 5 μm, Cellulose‐1 column (250×4.6 mm ID, a mixture of hexane:ethanol=85 : 15 as the eluent with a flow rate of 0.8 mL min^−1^, UV detector α=254 nm); UV detector 254 nm, 5 μL or 10 μL injection, 20 °C. Retention time for (*S*)‐**8**: 16.5 min, for (*R*)‐**8**: 18.1 min.

#### General procedure for Friedel–Crafts reaction of ethyl 3,3,3‐trifluoropyruvate (10) to indole (9)

To a solution of organocatalyst (0.012 mmol **SQ** or **TSQ** or **TU**) in DCM (1.5 mL) metal acetate (0.006 mmol Cu(OAc)_2_ or Ni(OAc)_2_ or AgOAc) was added. To this solution indole (**9**, 0.120 mmol) then ethyl 3,3,3‐trifluoropyruvate (**9**, 0.132 mmol) were added. The resulting mixture was stirred at room temperature for 24 hours. The volatile components were removed under reduced pressure. The crude product was purified by preparative thin layer chromatography on silica gel using dichloromethane (*R*_f_=0.57) as eluent to obtain the adduct as a colorless liquid. ^1^H NMR (500 MHz, CDCl_3_, 25 °C) δ 1.37 (t, *J*=7.5 Hz, 3H), 4.33–4.53 (m, 3 H), 7.19 (m, *J*=5.0 Hz, 1H), 7.26 (m, *J*=5.0 Hz, 1H), 7.36 (m, *J*=10.0 Hz, 1H), 7.44 (s, 1H), 7.94 (m, *J*=10.0 Hz, 1H), 8.28 (s, 1H); ^13^C NMR (125 MHz, CDCl_3_, 25 °C) δ 13.9, 64.2, 108.7, 111.4, 120.5, 121.1, 122.4, 122.7, 124.4, 124.7, 125.1, 136.3, 169.4 ppm. Yields and enantiomeric excess (*ee*) values can be seen in Table 2. These products had the same spectroscopic data than those of reported (the absolute configuration was determined by the optical rotation of the products).^[65]^ Phenomenex Lux® 5 μm, Cellulose‐1 column (250×4.6 mm ID, a mixture of water (0.1 % NH_4_OAc):MeCN=40 : 60 as the eluent with a flow rate of 0.8 mL min^−1^, UV detector α=254 nm); UV detector 254 nm, 5 μL or 10 μL injection, 20 °C. Retention time for (*S*)‐**11**: 12.3 min, for (*R*)‐**11**: 13.1 min.

#### General procedure for A^3^ reaction of aniline (12), benzaldehyde (13), and phenylacetylene (14)

To a solution of organocatalyst (0.010 mmol **SQ** or **TS** or **TU**) in the corresponding solvent (0.5 mL) metal acetate (0.005–0.012 mmol Cu(OAc)_2_ or Ni(OAc)_2_ or AgOAc) was added. To this solution aniline (**12**, 11.3 mg, 11 μL, 0.121 mmol), benzaldehyde (**13**, 10.6 mg, 10 μL, 0.100 mmol), finally phenylacetylene (**14**, 14.9 mg, 16 μL, 0.146 mmol) were added. Then the resulting mixture was stirred at room temperature for 24 hours. The volatile components were removed under reduced pressure. The crude product was purified by preparative thin layer chromatography on silica gel using dichloromethane:hexane=2 : 1 (*R*_f_=0.87) as eluent to obtain the adduct as a colorless liquid. Yields and enantiomeric excess (*ee*) values can be seen in Table 3. These products had the same spectroscopic data than those of reported (the absolute configuration was determined by the optical rotation of the products).^[80]^ Kromasil® AmyCoat 5 μm, Cellulose‐1 column (250×4.6 mm ID, a mixture of hexane:ethanol=85 : 15 as the eluent with a flow rate of 0.8 mL min^−1^, UV detector α=254 nm); UV detector 254 nm, 5 μL or 10 μL injection, 20 °C. Retention time for (*S*)‐**15**: 8.5 min, for (*R*)‐**15**: 9.7 min. The amounts of the catalysts and reaction times are shown in Table 3 and in Table S1 and Table S2 in Supporting Information.

#### General procedure for preparation of organocatalyst‐metal complexes

The appropriate metal acetate solution (AgOAc or Cu(OAc)_2_ or Ni(OAc)_2_, 0.025 mmol) in MeOH (0.5 mL) was added dropwise to the organocatalyst solution (**SQ** or **TSQ** or **TU**, 0.050 mmol) in DCM (1 mL) and stirred at room temperature for 5 minutes. The solvent was evaporated under reduced pressure to obtain the corresponding organocatalyst‐metal complex as a powder‐like solid. The HRMS measurement results of the prepared organocatalyst‐metal complexes can be seen in Table 4.

#### 2[3‐(Benzylamino)‐4‐(((S)‐((1S,2S,4S,5R)‐5‐ethylquinuclidin‐2‐yl)(6‐methoxyquinolin‐4‐yl)methyl)amino)cyclobut‐3‐ene‐1,2‐dithione].silver(I) acetate (2TSQ ⋅ AgOAc)

To a solution of **TSQ** (27.1 mg, 0.050 mmol), AgOAc (4.2 mg, 0.025 mmol) in MeOH (0.5 mL) was added dropwise, and stirred at room temperature for 5 minutes. The solvent was evaporated under reduced pressure to obtain **2TSQ ⋅ Ag(I)** complex as a yellow powder‐like solid (31 mg, 99 %). ^1^H NMR (600 MHz, DMSO‐d_6_, 50 °C) δ 8.80–8.78 (m, 1 H), 8.78–8.76 (m, 1 H), 8.03–8.01 (m, 1 H), 8.01–7.95 (m, 3 H), 7.76–7.70 (m, 2 H), 7.47–7.44 (m, 2 H), 7.44–7.40 (m, 2 H), 7.35–7.30 (m, 4 H), 7.29–7.26 (m, 1 H), 7.25–7.22 (m, 1 H), 7.21–7.15 (m, 2 H), 7.00–6.79 (m, 2 H), 5.49–5.39 (m, 1 H), 5.22–5.10 (m, 2 H), 4.68–4.61 (m, 1 H), 4.28–4.19 (m, 1 H), 4.19–4.03 (m, 2 H), 4.00–3.97 (m, 1 H), 3.94 (s, 6 H), 3.86 (m, 2 H), 3.60–3.48 (m, 2 H), 3.18–3.06 (m, 2 H), 3.05–2.98 (m, 1 H), 2.94–2.87 (m, 1 H), 2.09 (s, 2 H), 1.94–1.74 (m, 9 H), 1.67–1.57 (m, 2 H), 1.45–1.37 (m, 2 H), 1.36–1.28 (m, 2 H), 1.17–1.10 (m, 1 H), 1.07–1.00 (m, 1 H), 0.81 (6 H, t, *J*_H,H_ 6.0 Hz); ^13^C NMR (150 MHz, CDCl_3_, 25 °C) δ 206.7, 206.1, 178.1, 176.7, 164.1, 163.0, 157.9, 157.7, 148.0, 147.7, 147.6, 144.5, 137.8, 137.2, 131.7, 131.6, 128.8, 128.7, 128.6, 128.6, 128.2, 128.1, 128.0, 127.8, 127.4, 127.3, 127.3, 122.3, 122.1, 121.7, 119.5, 102.6, 77.0, 69.4, 58.6, 57.6, 56.4, 56.1, 55.2, 55.1, 55.0, 47.9, 47.8, 47.6, 41.4, 41.3, 40.2, 36.3, 34.5, 34.4, 30.9, 26.0, 25.4, 25.3, 25.0, 24.5, 24.3, 24.1, 24.0, 23.9, 23.1, 22.8, 11.8, 11.6, 11.5. HRMS‐ESI+ (m/z): [M^+^] calcd. for C_62_H_68_N_8_O_2_S_4_ ⋅ Ag: 1191.3399, found: 1191.3420.

## Conflict of interests

The authors declare no conflict of interest.

1

## Supporting information

As a service to our authors and readers, this journal provides supporting information supplied by the authors. Such materials are peer reviewed and may be re‐organized for online delivery, but are not copy‐edited or typeset. Technical support issues arising from supporting information (other than missing files) should be addressed to the authors.

Supporting Information

## Data Availability

The data that support the findings of this study are available in the supplementary material of this article.
