# Supplementary material for: Cinchona‐Based Hydrogen‐Bond Donor Organocatalyst Metal Complexes: Asymmetric Catalysis and Structure Determination
Source: ChemistryOpen. 2024 Jan 8;13(4):e202300180. doi: 10.1002/open.202300180 (PMC11004460; doi:10.1002/open.202300180)
Supplement: Supplementary file 1 — Supporting Information [file OPEN-13-e202300180-s001.pdf]

ChemistryOpen

Supporting Information

Cinchona-Based Hydrogen-Bond Donor Organocatalyst Metal Complexes: Asymmetric Catalysis and Structure Determination

Sándor Nagy, Dóra Richter, Gyula Dargó, Balázs Orbán, Gergő Gémes, Tibor Höltzl, Zsófia Garádi, Zsuzsanna Fehér, and József Kupai*

1 Further results of A^3 reaction 2

2 FT-IR spectra 4

3 NMR spectra 5

3.1 NMR spectra of TU 5

3.2 NMR spectra of 8 6

3.3 NMR spectra of 11 8

3.4 NMR spectra of 2TSQ·Ag 9

4 MS spectra 13

4.1 2SQ·AgOAc 13

4.2 2TSQ·AgOAc 13

4.3 2TU·AgOAc 14

4.4 2SQ·$Cu(OAc)_2$ 14

4.5 2TSQ·$Cu(OAc)_2$ 15

4.6 2TU·$Cu(OAc)_2$ 15

4.7 2SQ·$Ni(OAc)_2$ 16

4.8 2TSQ·$Ni(OAc)_2$ 16

4.9 2TU·$Ni(OAc)_2$ 17

5 Chiral HPLC profiles 18

5.1 Michael adduct (8) 18

5.2 Friedel–Crafts adduct (11) 19

5.3 A^3 adduct (15) 21

6 Quantum chemical computations 22

6.1. Complex formation with two ligands 22

6.1.1. S-Ni-S complex 22

6.1.2. N-Ni-S and N-Ni-N complexes 24

6.2. Complex formation with one ligand 26

6.3. Stability of Ni(II) complexes with one or two thiosquaramide ligands 30

6.4. Comparison of thiosquaramide (TSQ), thiourea (TU) and squaramide (SQ) ligands 30

6.5. Analysis of L-Ni(II)-Acetate complexes 35

6.5.1. Natural Bond Orbital (NBO) analysis 35

6.5.2. Energy decomposition analysis (EDA) 36

6.6. Complex formation with one ligand in different solvents 37

6.7. Data of optimized structures 39

6.7.1. Basic structures 39

6.7.2. Structures of ligands 41
6.7.3. Structures of complexes with one ligand 51
6.7.4. Structures of complexes with two ligands 96

1 Further results of A^3 reaction

Table S1. Preparative yields of A^3 reaction in six different solvents and applying different organocatalyst–Cu(II) ratio

Organo-catalyst	OrgCat:Cu ratio	Entries	Solvent: MeCN 1	MeOH 2	toluene 3	THF 4	DMSO 5	DCM 6
TSQ	1.2:1	A	traces	traces	traces	traces	7	0
	1:1	B	0	0	0	0	0	0
	1:1.2	C	0	0	0	0	0	0
	2:1	D	0	0	0	0	0	0
TU	1.2:1	E	traces	traces	18	33	8	0
	1:1	F	0	0	32	21	6	0
	1:1.2	G	0	0	26	22	13	0
	2:1	H	0	0	9	5	6	0
SQ	1.2:1	I	0	0	34	7	0	14
	1:1	J	0	0	17	29	0	28
	1:1.2	K	0	0	6	17	0	0
	2:1	L	0	6	8	0	0	0

Table S2. Enantiomeric excess values of A^3 reaction in six different solvents and applying different organocatalyst–Cu(II) ratio

Catalyst	Cat:Cu ratio	Entries	Solvent: MeCN 1	MeOH 2	toluene 3	THF 4	DMSO 5	DCM 6
TSQ	1.2:1	A	0	0	0	0	8	0
	1:1	B	0	0	0	0	0	0
	1:1.2	C	0	0	0	0	0	0
	2:1	D	0	0	0	0	0	0
TU	1.2:1	E	0	0	5	15*	0	0
	1:1	F	0	0	0	13*	0	0
	1:1.2	G	0	0	0	14*	0	0
	2:1	H	0	0	9	12*	0	0
SQ	1.2:1	I	0	0	23	0	0	7
	1:1	J	0	0	22	2	0	10
	1:1.2	K	0	0	19	3	0	0
	2:1	L	0	0	6	0	0	0

*Opposite enantiomer (*R*)

Based on the best results, better yields and enantiomeric excess values were expected in ether-, or in hydrocarbon-type solvents with organocatalyst:Cu(II) = 1.2:1 ratio. The results are shown in Table S3.

Table S3. Preparative yields and enantiomeric excess values of A^3 reaction in methyl *tert*-butyl ether (MTBE), mesitylene and anisole

Organo-catalyst	OrgCat:Cu ratio	Solvent					
		MTBE		Mesitylene		Anisole	
		Yield (%)	ee (%)	Yield (%)	ee (%)	Yield (%)	ee (%)
**TU**	1.2:1	3	0	2	0	5	0
**SQ**	1.2:1	4	7	3	6	3	7

2 FT-IR spectra

1.1FT-IR spectrum of TU

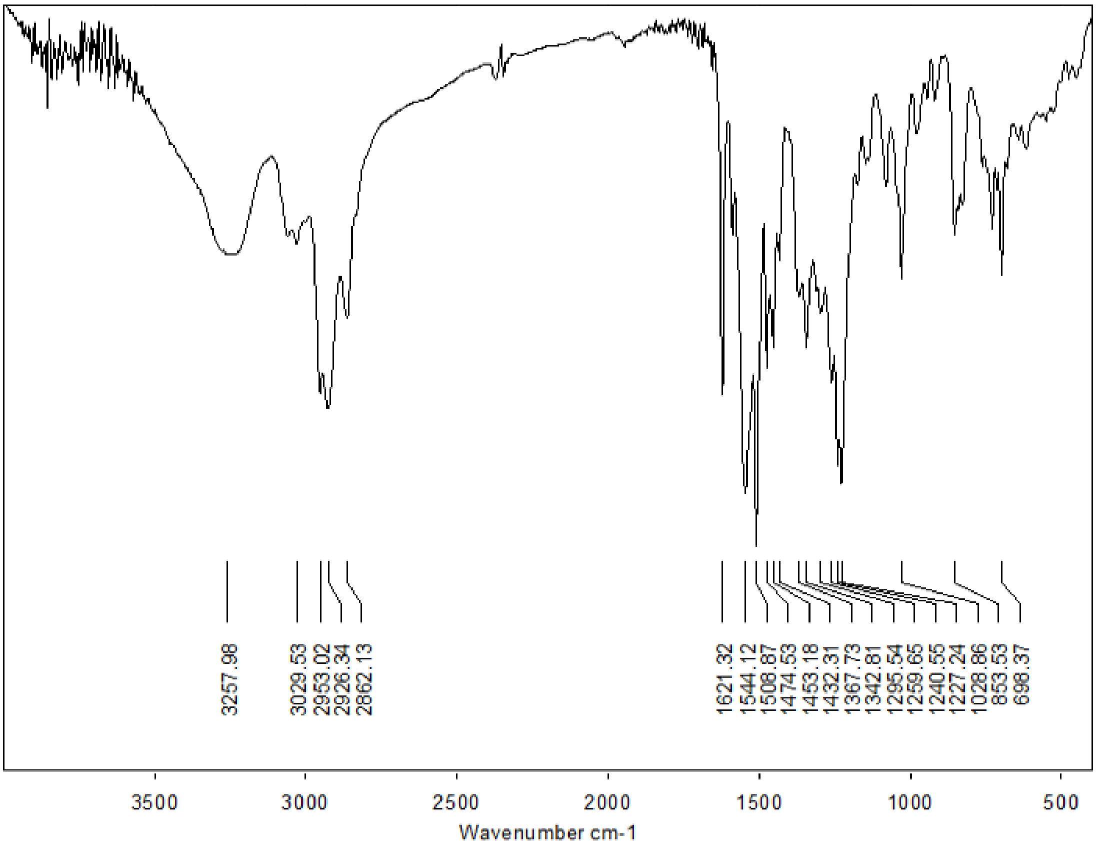

Figure S1 FT-IR spectrum of **TU**

3 NMR spectra

3.1 NMR spectra of TU

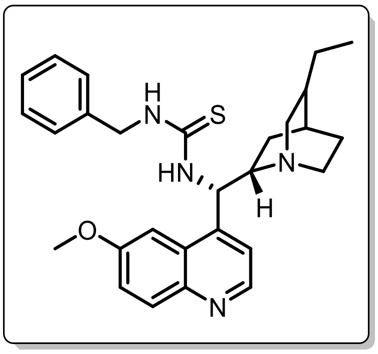

Figure S2 Molecule structure of **TU**

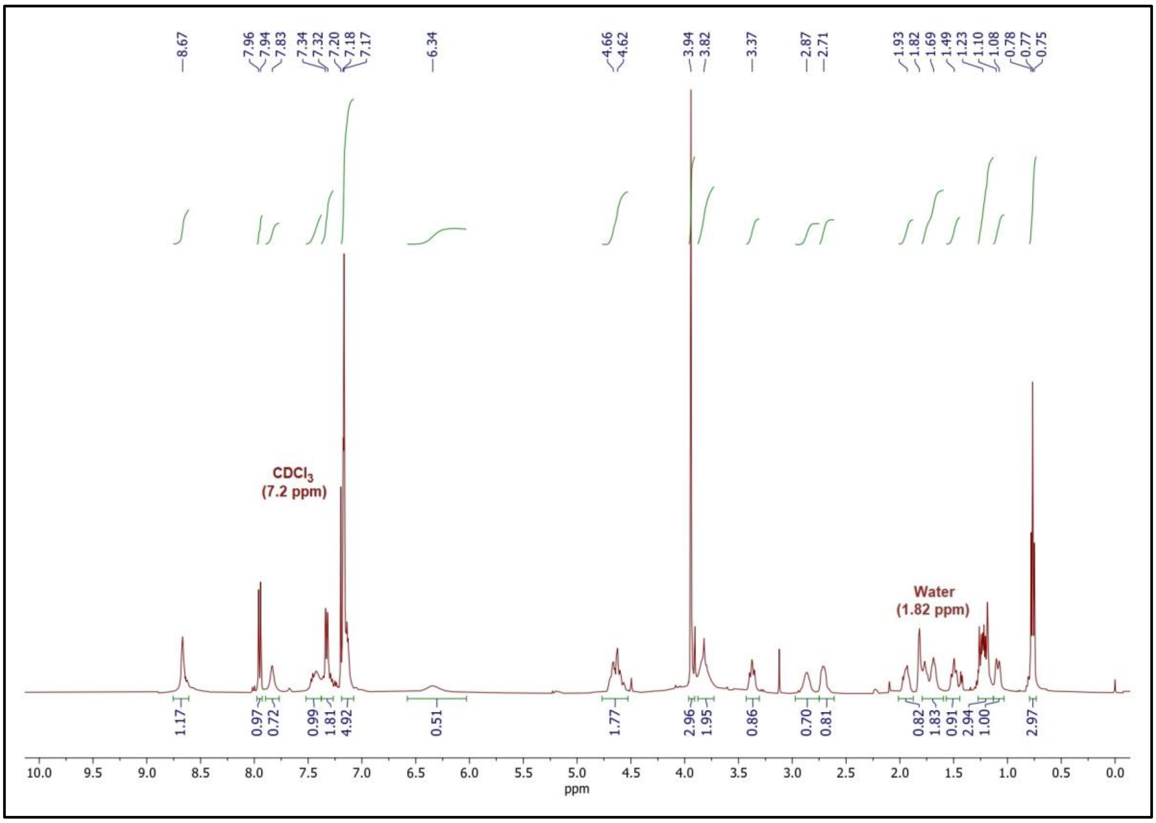

Figure S3 ^{1}H NMR spectrum of **TU** (500 MHz, $CDCl_3$, 25 °C)

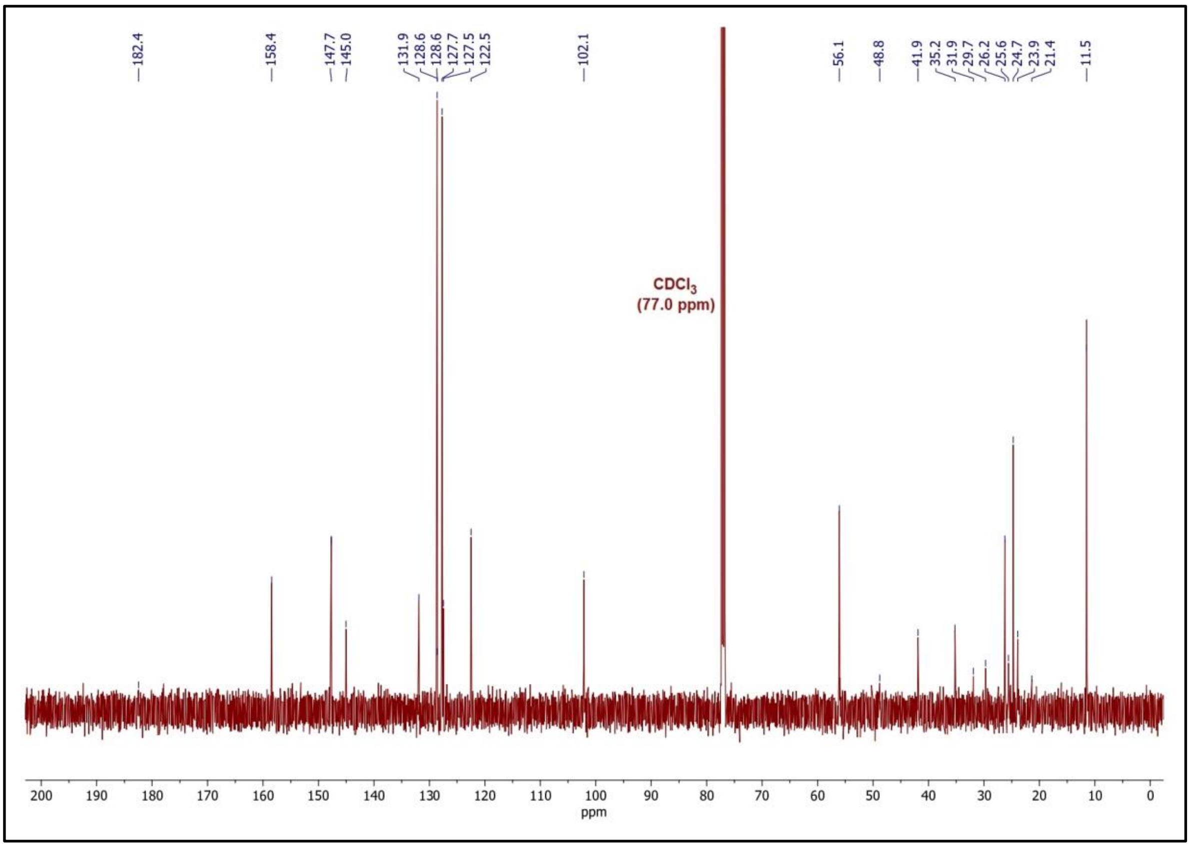

Figure S4 ^{13}C NMR spectrum of **TU** (125 MHz, $CDCl_3$, 25 °C)

3.2 NMR spectra of 8

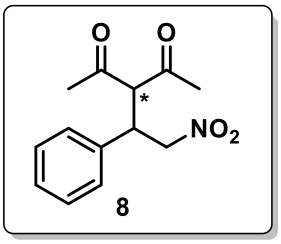

Figure S5 Molecule structure of **8**

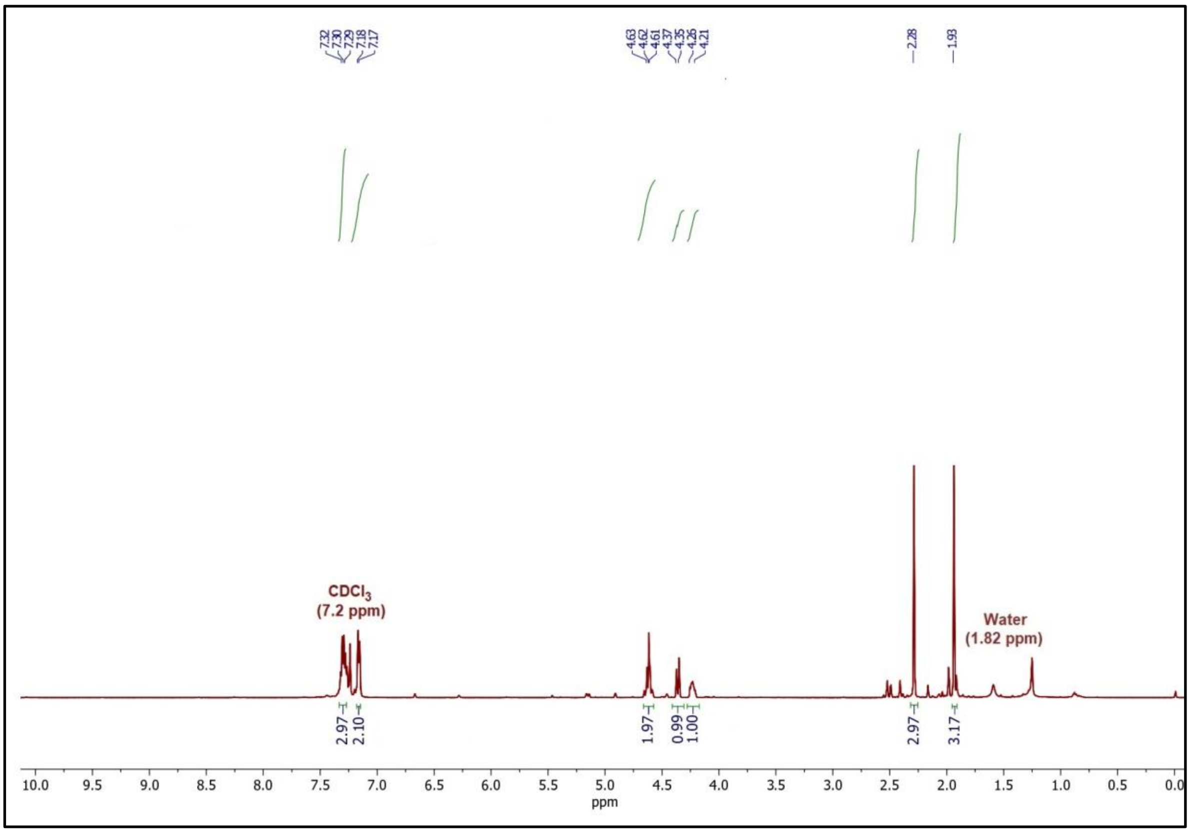

Figure S6 ^{1}H NMR spectrum of **8** (500 MHz, $CDCl_3$, 25 °C)

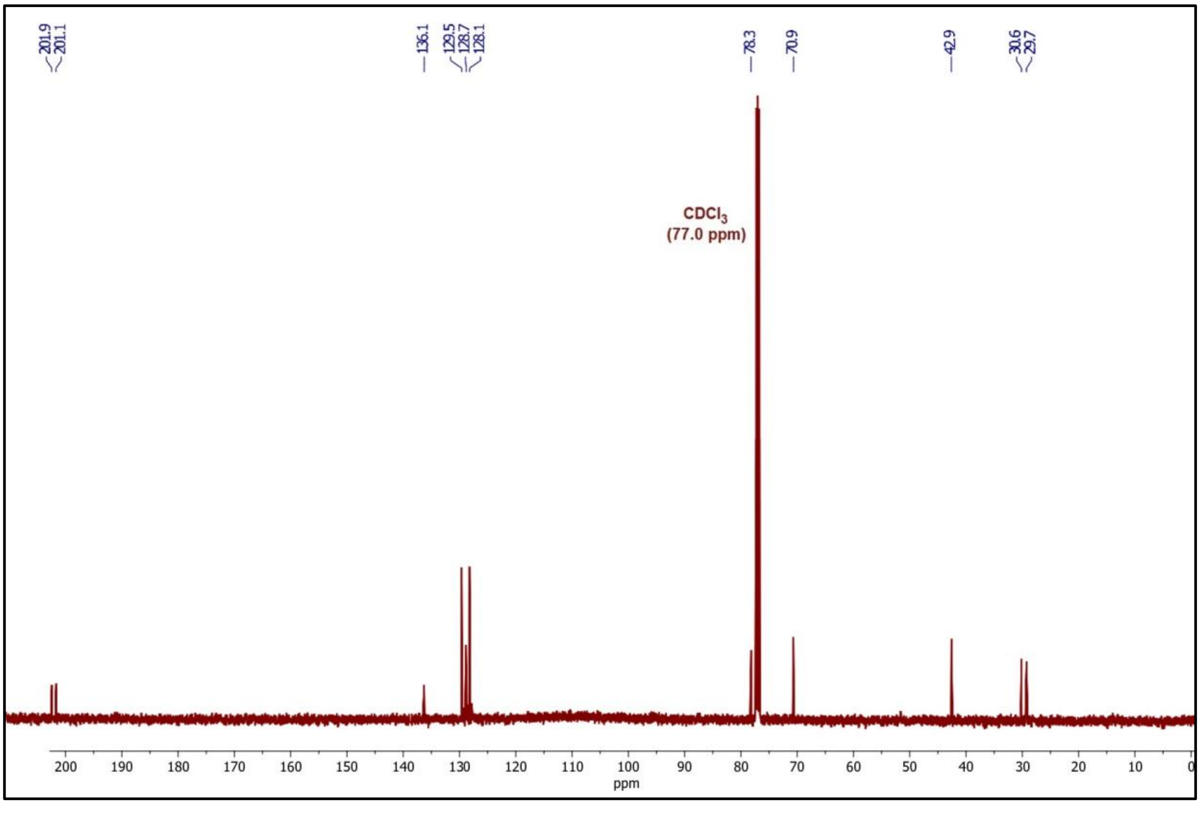

Figure S7 ^{13}C NMR spectrum of **8** (125 MHz, $CDCl_3$, 25 °C)

3.3 NMR spectra of 11

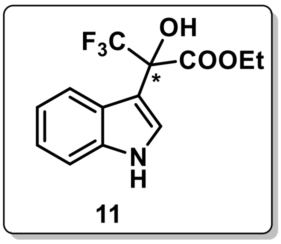

Figure S8 Molecule structure of **11**

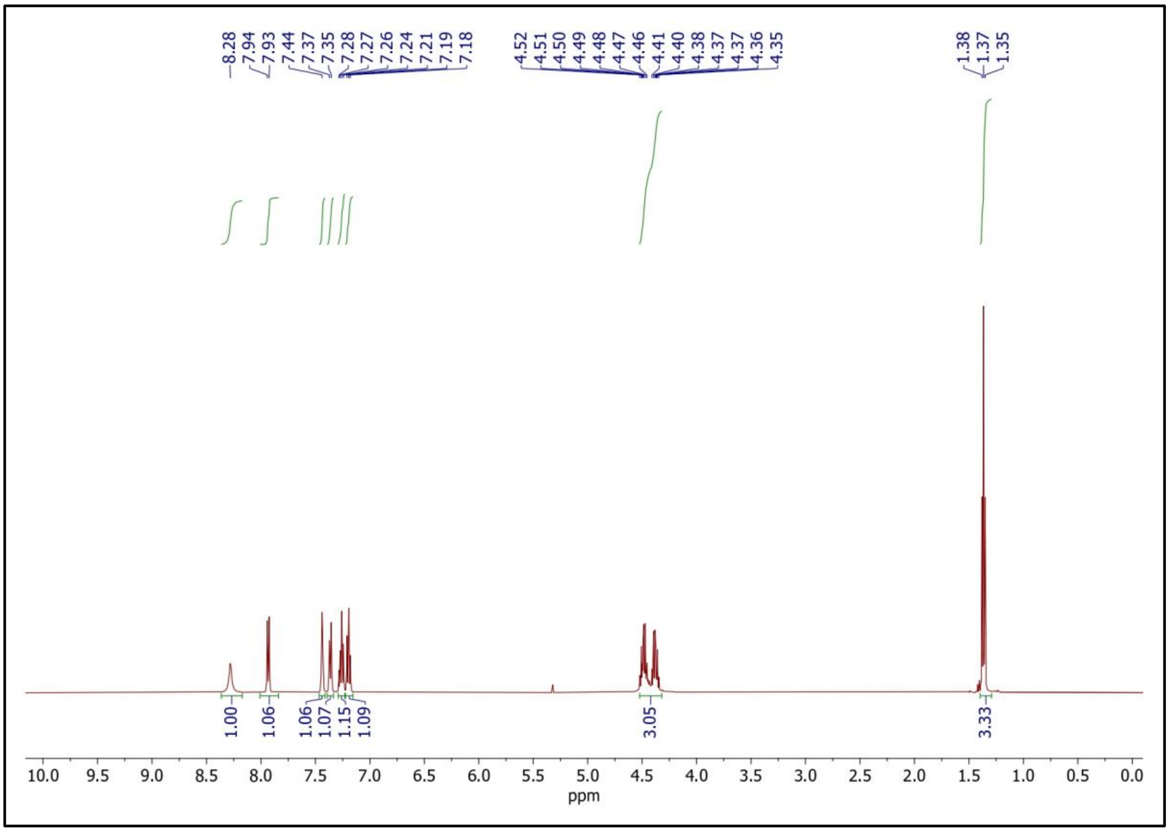

Figure S9 ^{1}H NMR spectrum of **11** (500 MHz, $CDCl_3$, 25 °C)

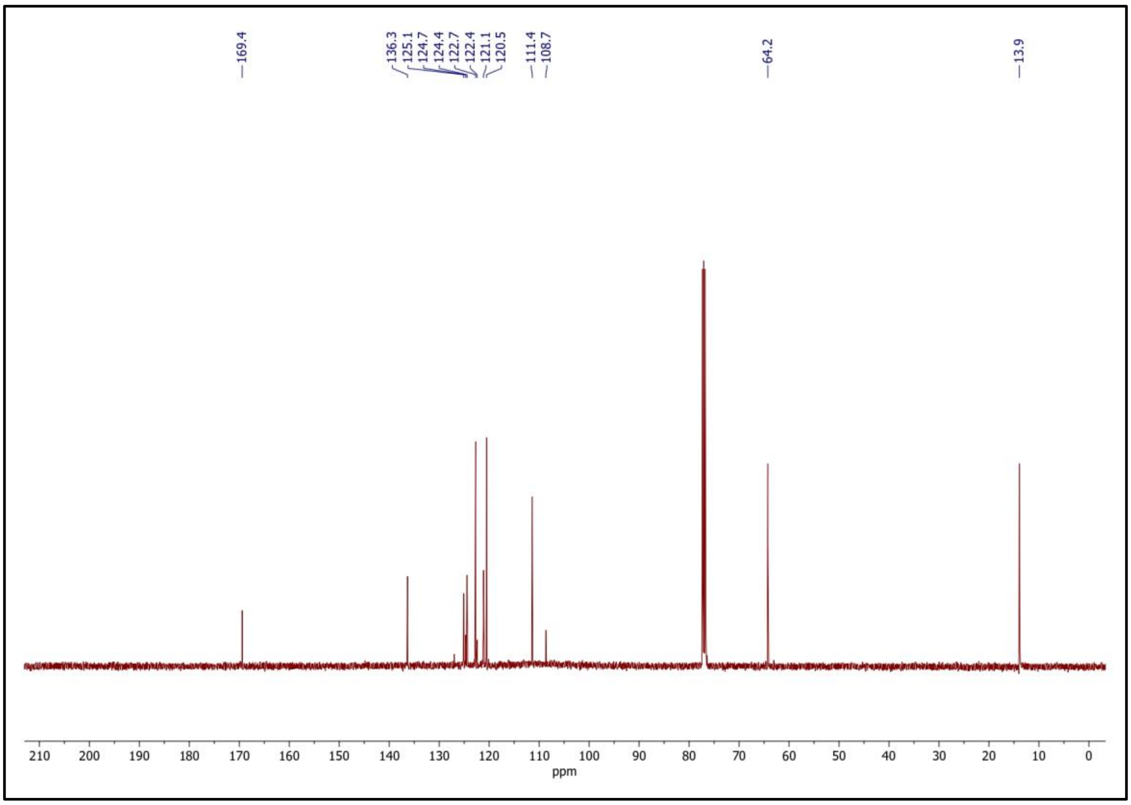

Figure S10 ^{13}C NMR spectrum of **11** (125 MHz, $CDCl_3$, 25 °C)

3.4 NMR spectra of 2TSQ·Ag

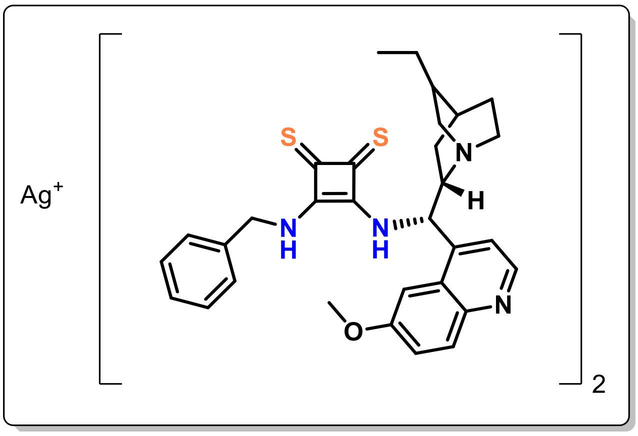

Figure S11 Molecule structure of **2TSQ·Ag**

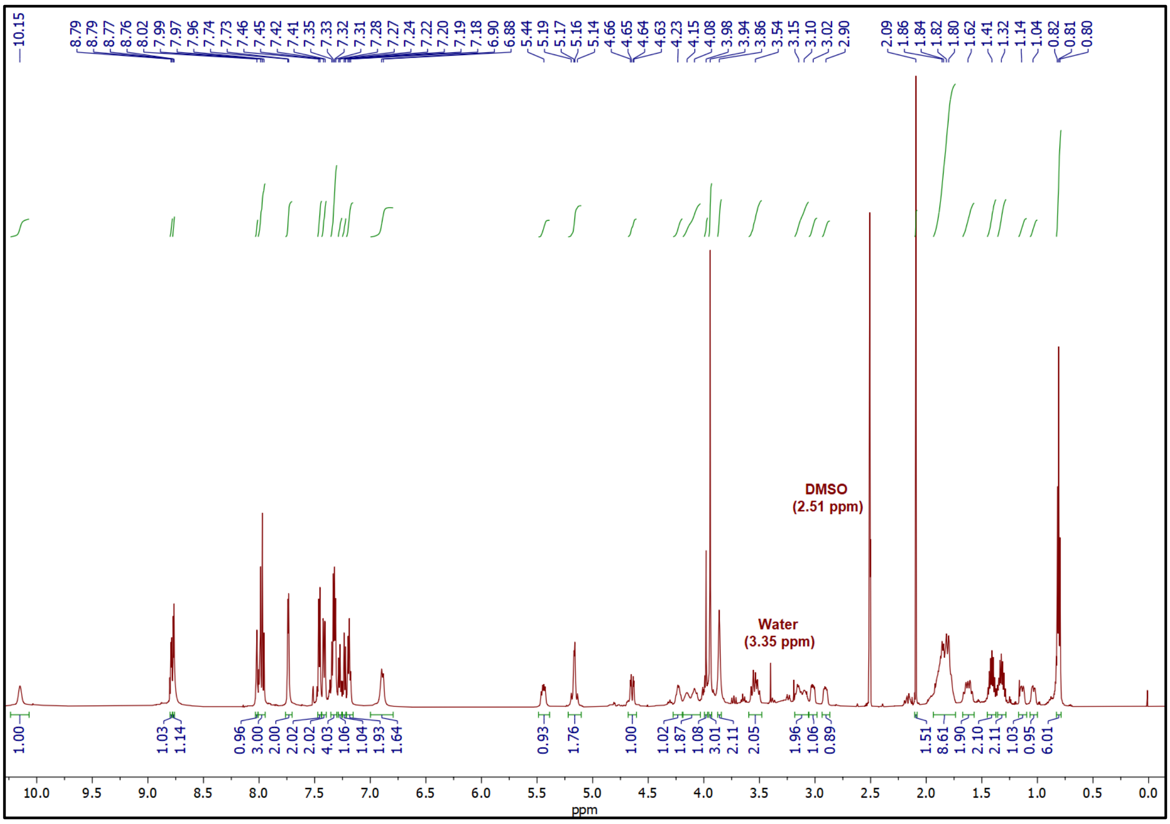

Figure S12 ^{1}H NMR spectrum of 2**TSQ**·Ag complex (600 MHz, DMSO, 50 °C)

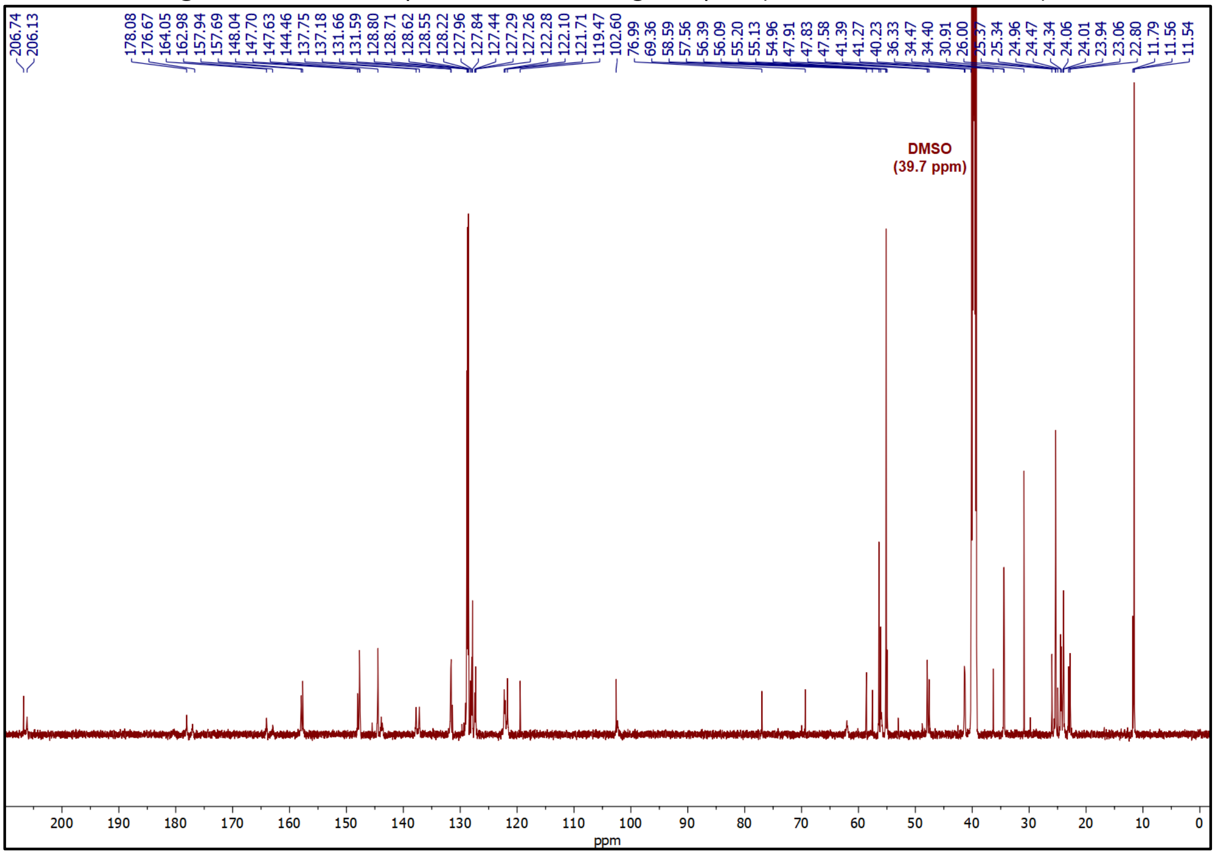

Figure S13 ^{13}C NMR spectrum of 2**TSQ**·Ag complex (150 MHz, DMSO, 50 °C)

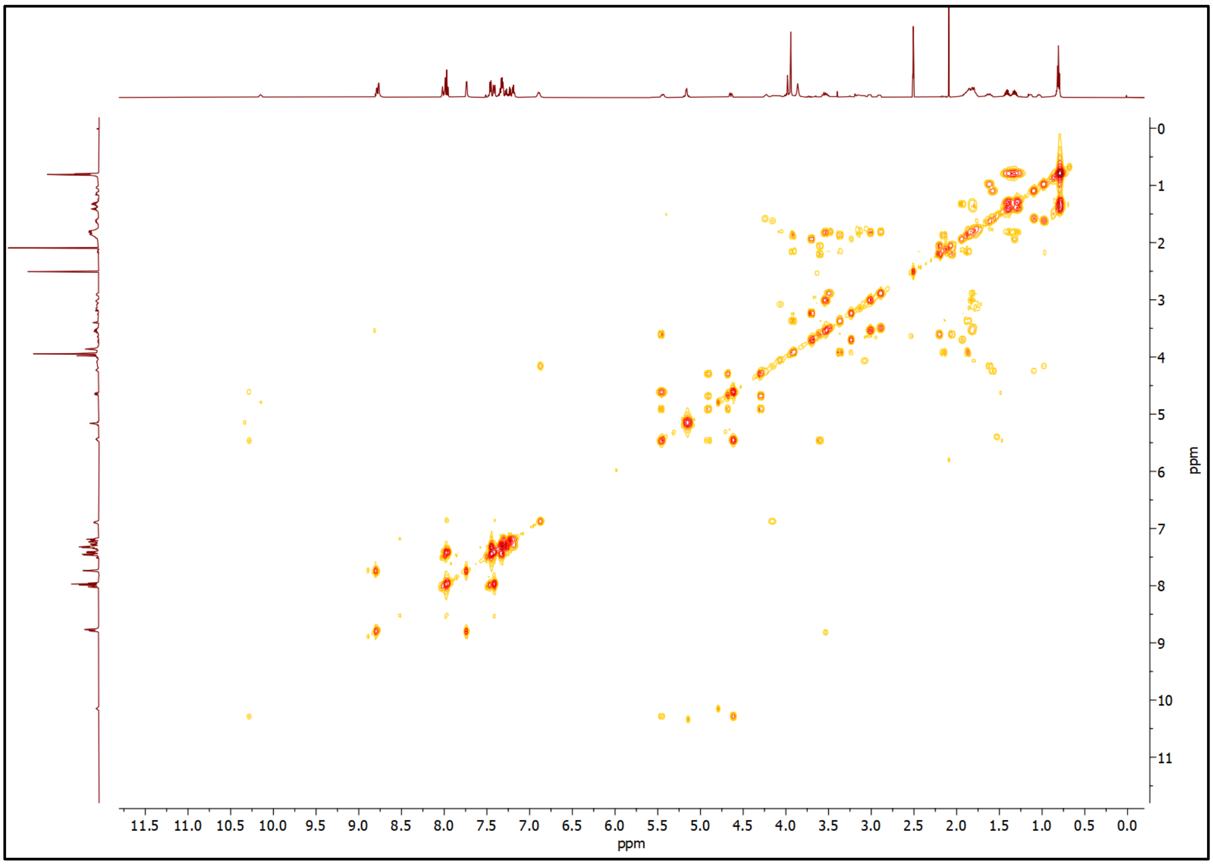

Figure S14. COSY NMR spectrum of 2**TSQ**·Ag complex (DMSO, 50 °C)

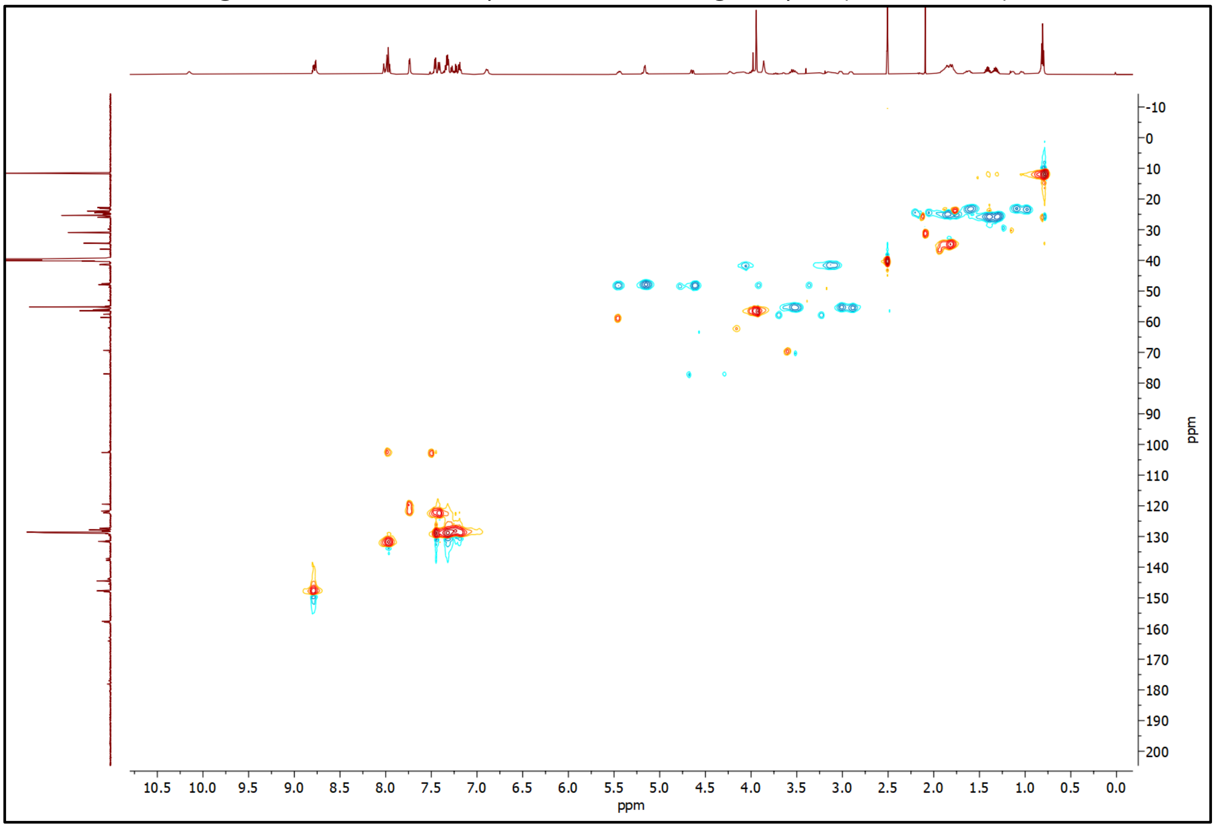

Figure S15. HSQC NMR spectrum of 2**TSQ**·Ag complex (DMSO, 50 °C)

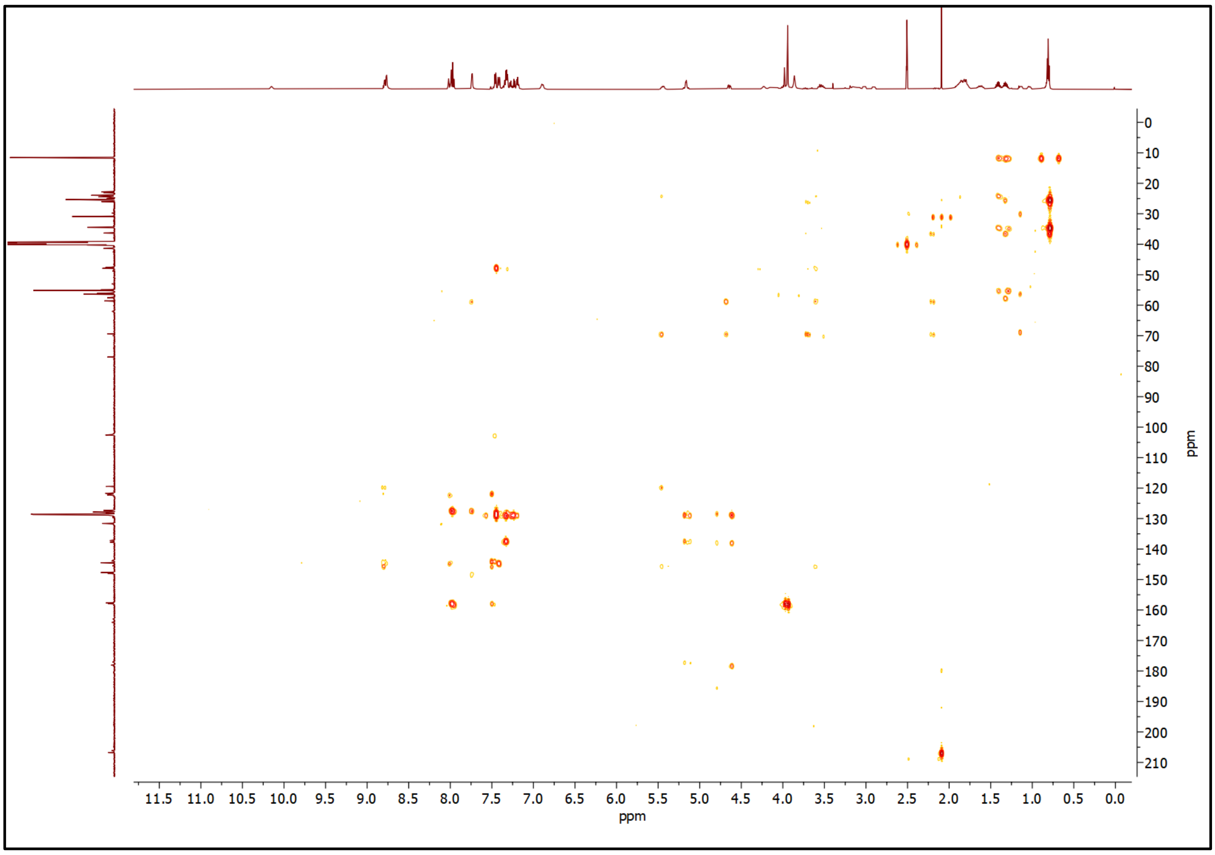

Figure S16. HMBC NMR spectrum of 2**TSQ**·Ag complex (DMSO, 50 °C)

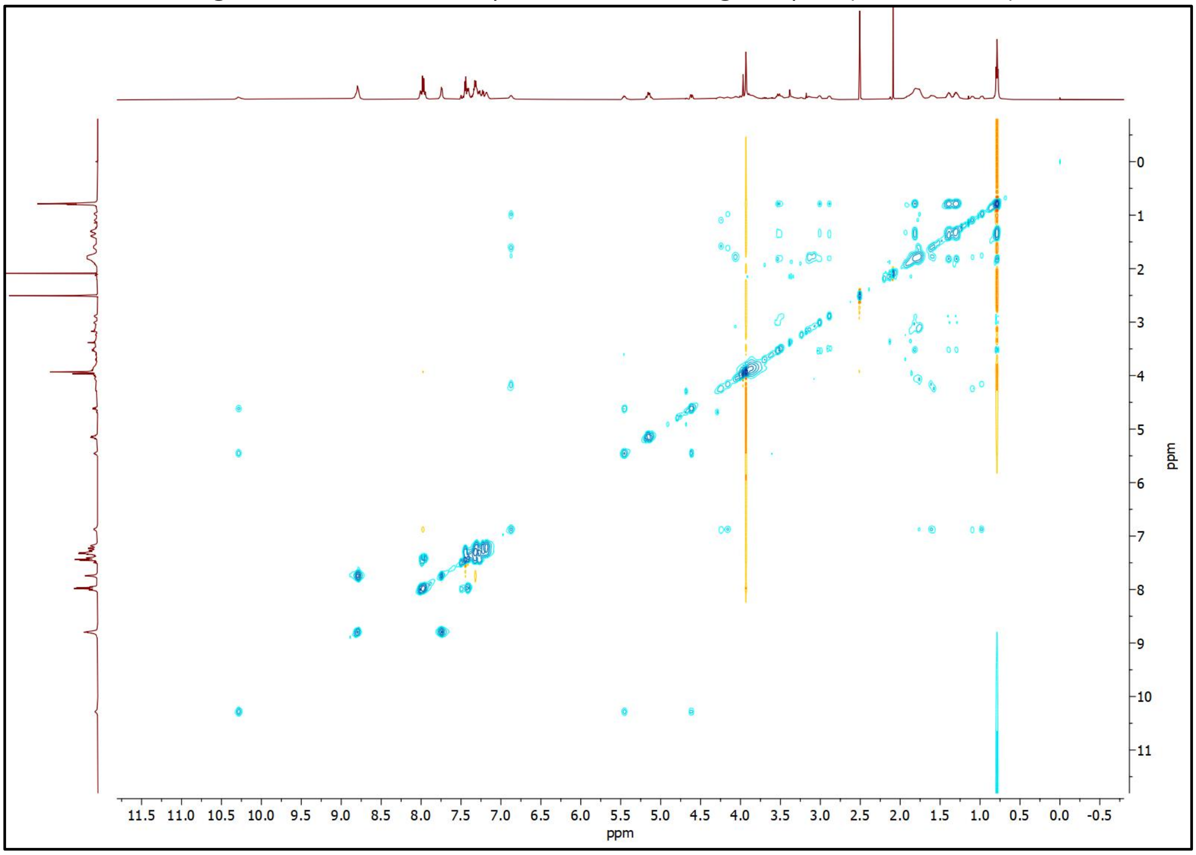

Figure S17. TOCSY NMR spectrum of 2**TSQ**·Ag complex (DMSO, 50 °C)

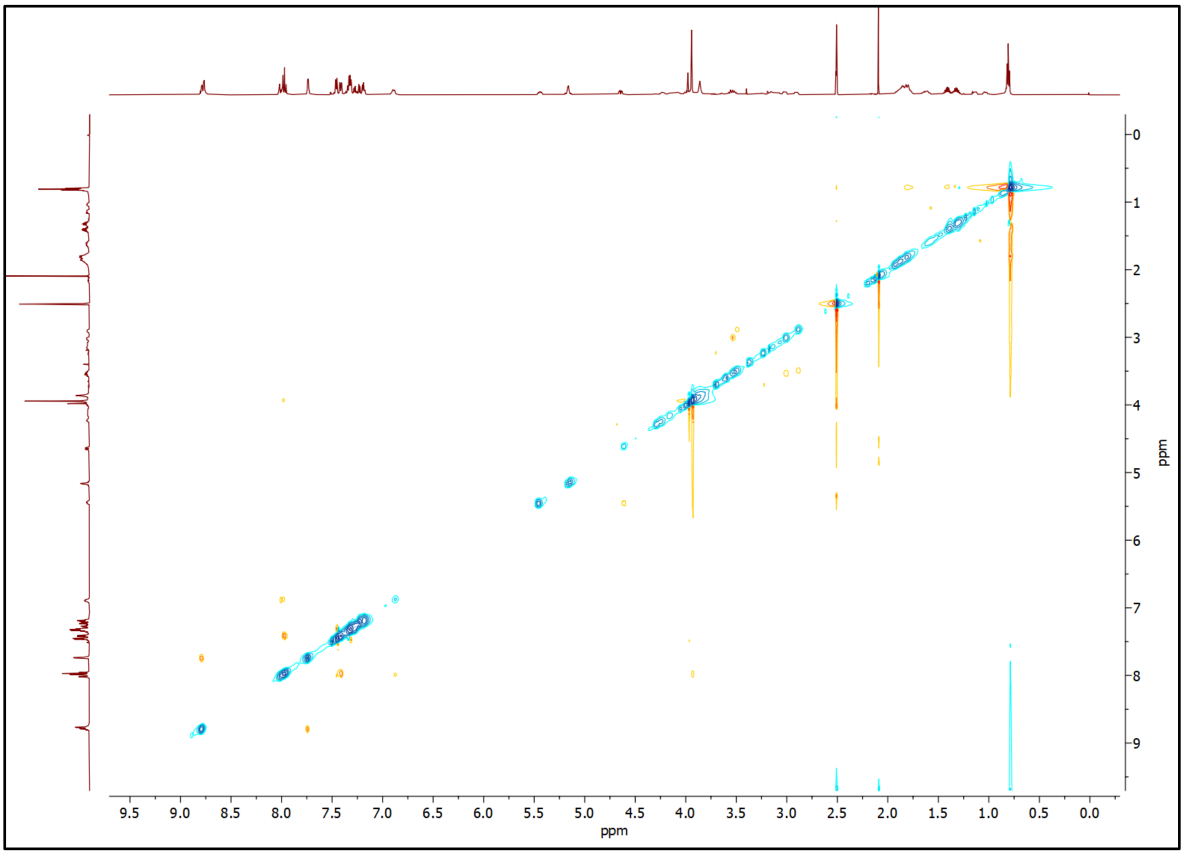

Figure S18. ROESY NMR spectrum of 2**TSQ**·Ag complex (DMSO, 50 °C)

4 MS spectra

Table S4. The observed specimen and HRMS measurement results of organocatalyst–metal complexes, and their appearance

Entry	Complex	Observed specimen	HRMS-ESI Calcd	HRMS-ESI Found	Difference (ppm)	Appearance
1	2SQ.AgOAc	Complex was not detected in HRMS			-	Grey powder
2	2TSQ.AgOAc	M$^+$ [2TS.Ag(I)]	1191.3399	1191.3420	1.8	Yellow poweder
3	2TU.AgOAc	Complex was not detected in HRMS			-	Black powder
4	2SQ·Cu(OAc)$_2$	M-H [2SQ·Cu(II)]	1082.4480	1082.4498	1.7	Dark brown powder
5	2TSQ·Cu(OAc)$_2$	M$^+$ [2TS·Cu(II)]	1147.3644	1147.3662	1.6	Dark brown powder
6	2TU·Cu(OAc)$_2$	M-H$^+$ [2TU·Cu(II)]	1010.4124	1010.4114	-1.0	Dark green powder
7	2SQ.Ni(OAc)$_2$	Complex was not detected in HRMS			-	Light green powder
8	2TSQ.Ni(OAc)$_2$	M-H [2TS.Ni(II)]	1141.3623	1141.3621	-0.2	Dark red powder
9	2TU.Ni(OAc)$_2$	M-H$^+$ [2TU.Ni(II)]	1005.4182	1005.4169	-1.3	Off-white powder

4.1 2SQ·AgOAc

Complex was not detected in HRMS.

4.2 2TSQ·AgOAc

The molecule ion (**2TSQ·Ag**$^+$, M$^+$ = 1193) is detected in spectrum, protonated molecule ion of organocatalyst moiety (**TSQ**, [M+H]$^+$ = 543) gives intensive peak (Figure S19). M/z = 651 can presumably be TSQ + Ar. Isotopic distribution of molecule matches the expected Ag content (Figure **S20**).

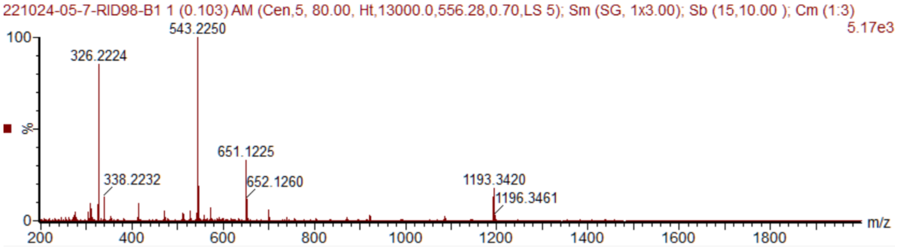

Figure S19. HRMS Spectrum of 2TSQ·AgOAc

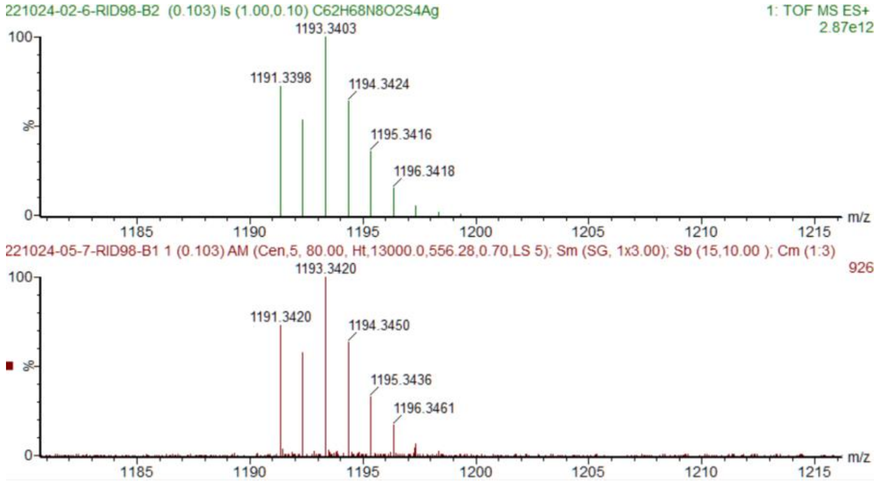

Figure S20. The theoretical (above) and measured (below) isotopic distribution of 2TSQ·AgOAc

4.3 2TU·AgOAc

Complex was not detected in HRMS.

4.4 2SQ·Cu(OAc)$_2$

Deprotonated form of molecule (**2SQ·Cu−H**$^+$, [M−H]$^+$ = 1082) can be detected in spectrum, protonated molecule ion of organocatalyst moiety (**SQ**, [M+H]$^+$ = 511) gives intensive peak (Figure S21). Molecule ion of **2SQ·Cu** can be detected in isotopic distribution overlapped with the second peak of **2SQ·Cu−H**$^+$ (Figure S22). The isotopic distribution matches the two structure's (**2SQ·Cu** and **2SQ·Cu−H**$^+$) superimposed spectrum (Figure S22).

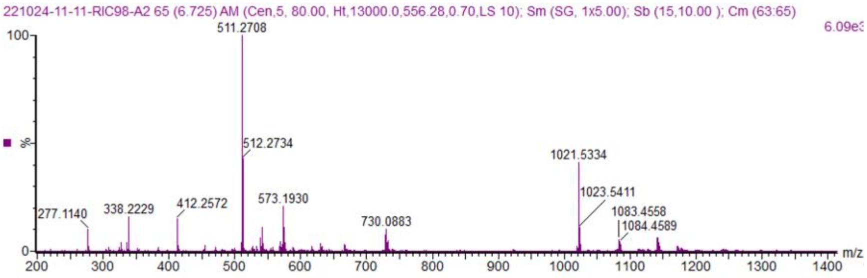

Figure S21 HRMS Spectrum of 2SQ·Cu(OAc)$_2$

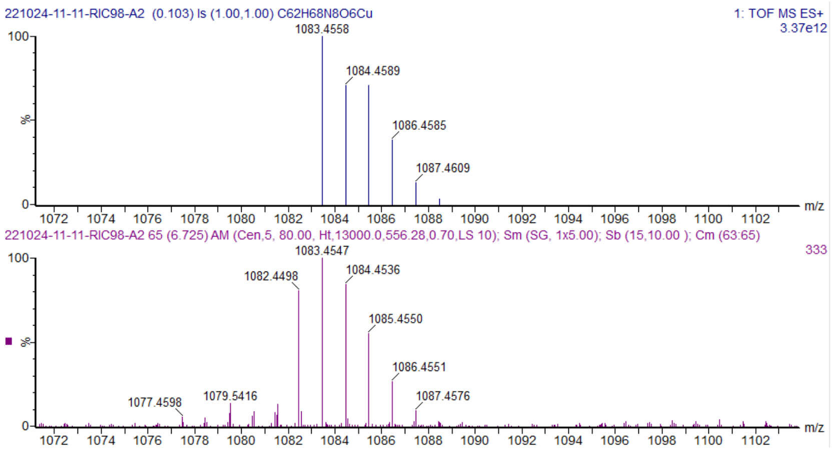

Figure S22. The theoretical (above) and measured (below) isotopic distribution of 2SQ·Cu(OAc)$_2$

4.5 2TSQ·Cu(OAc)$_2$

Molecule ion (**2TSQ·Cu$^+$**, $[M]^+$ = 1147) and the protonated molecule ion of organocatalyst moiety (**TSQ+H$^+$**, $[M+H]^+$ = 543) can be detected in spectrum (**Figure S23**). M/z = 605 can presumably be TSQ + Cu. Isotopic distribution of molecule shows the expected Cu content (**Figure S24**).

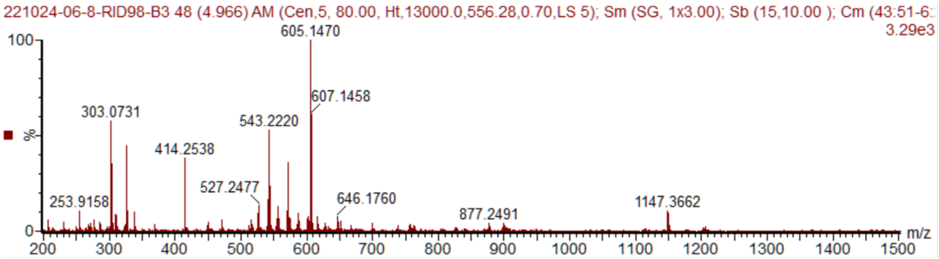

Figure S23 HRMS Spectrum of 2TSQ·Cu(OAc)$_2$

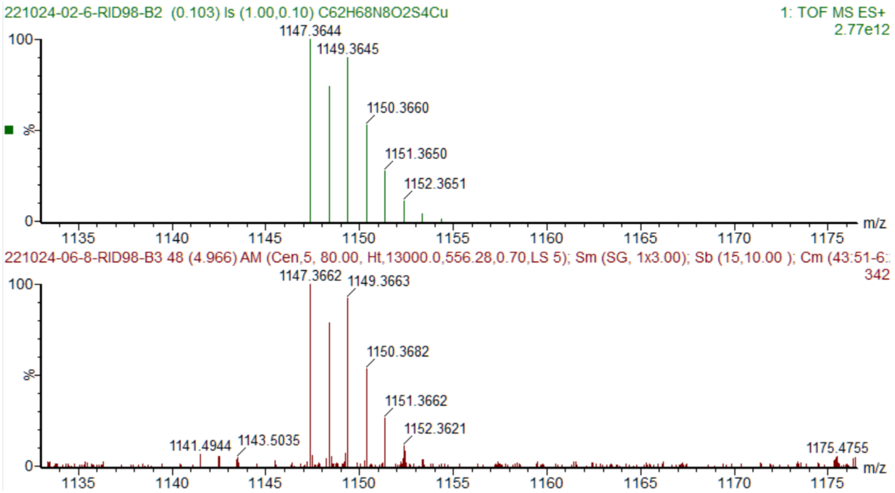

Figure S24 The theoretical (above) and measured (below) isotopic distribution of 2TSQ·Cu(OAc)$_2$

4.6 2TU·Cu(OAc)$_2$

Deprotonated form of molecule (**2TU·Cu–H$^+$**, $[M-H]^+$ = 1010) and molecule ion (**2TU·Cu$^+$**, $[M]^+$ = 1011, overlapped with the second isotopic peak of **2TU·Cu–H$^+$**) can be detected in spectrum, protonated molecule ion of organocatalyst moiety (**TU**, $[M+H]^+$ = 475) gives intensive peak (Figure S25). The

isotopic distribution matches the two structure’s (**2SQ·Cu** and **2SQ·Cu−H**$^+$) superimposed spectrum (Figure S26).

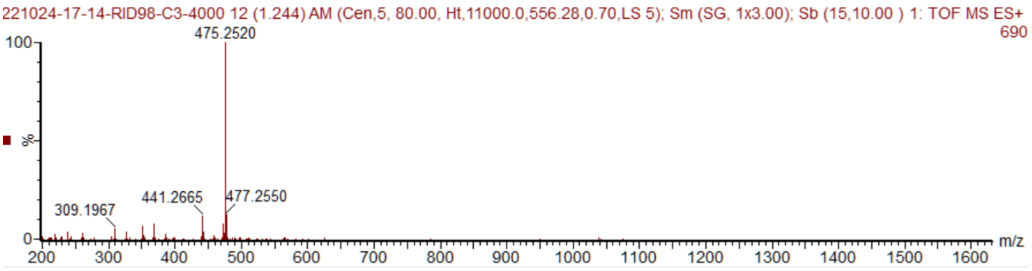

Figure S25 HRMS Spectrum of 2TU·Cu(OAc)$_2$

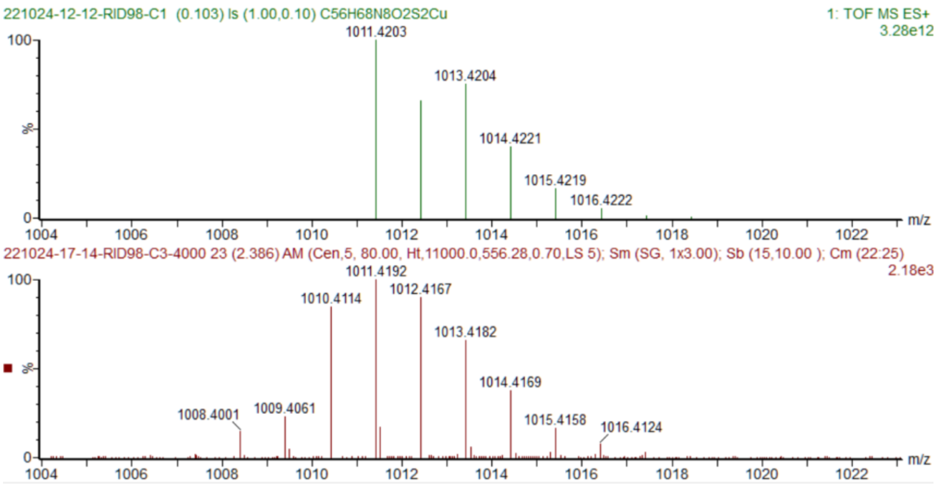

Figure S26 The theoretical (above) and measured (below) isotopic distribution of 2TU·Cu(OAc)$_2$

4.7 2SQ·Ni(OAc)$_2$

Complex was not detected in HRMS.

4.8 2TSQ·Ni(OAc)$_2$

Deprotonated form of molecule (**2TSQ·Ni−H**$^+$, $[M{-}H]^+$ = 1141) can be detected in spectrum, doubly protonated molecule ion (**2TSQ·Ni+2H**$^+$, M^{2+} = 571) and protonated molecule ion of organocatalyst moiety (**TSQ**, $[M{+}H]^+$ = 543) give intensive peaks (Figure S27). Isotopic distribution of molecule matches the expected Ag content (Figure S28).

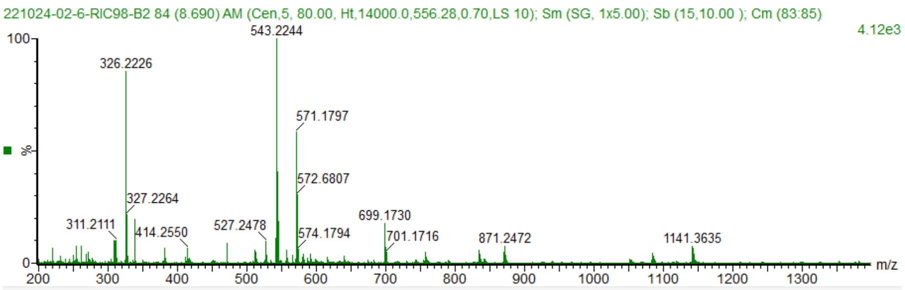

Figure S27 HRMS Spectrum of 2TSQ·Ni(OAc)$_2$

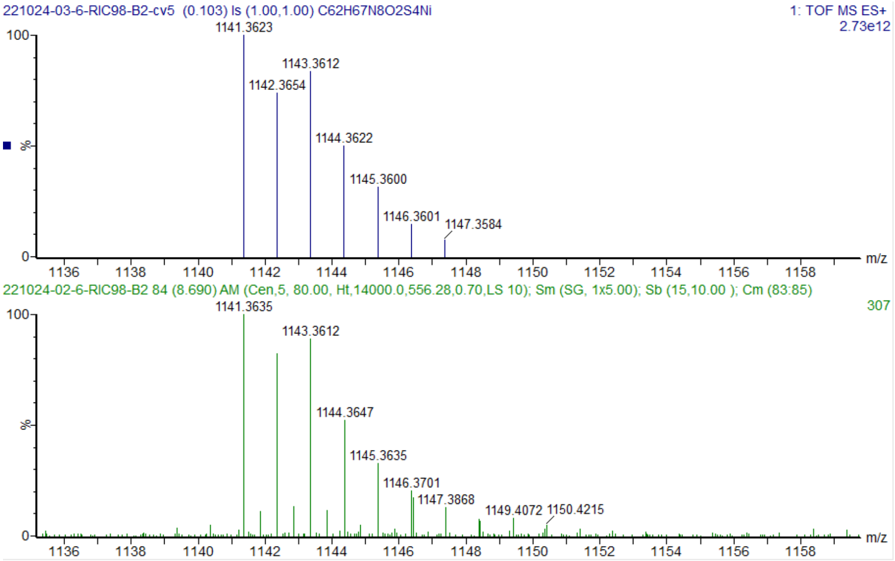

Figure S28 The theoretical (above) and measured (below) isotopic distribution of 2TSQ·Ni(OAc)$_2$

4.9 2TU·Ni(OAc)$_2$

Deprotonated form of molecule (**2TU·Ni–H**$^+$, [M−H]$^+$ = 1005) can be detected in spectrum, the protonated molecule ion of organocatalyst moiety (**TU+H**$^+$, [M+H]$^+$ = 475) gives intensive peak (Figure S29). Isotopic distribution of molecule matches the expected Ni content (Figure S30).

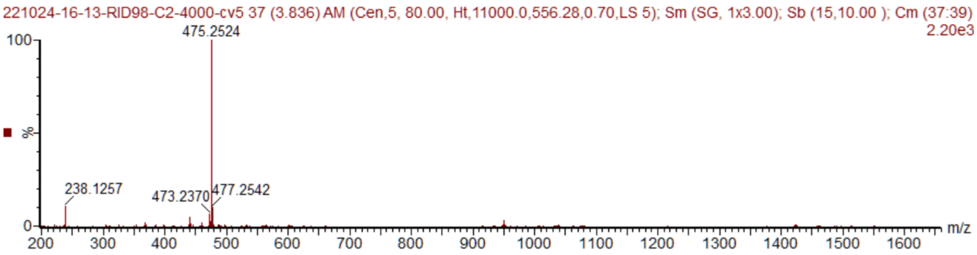

Figure S29 HRMS Spectrum of 2TU·Ni(OAc)$_2$

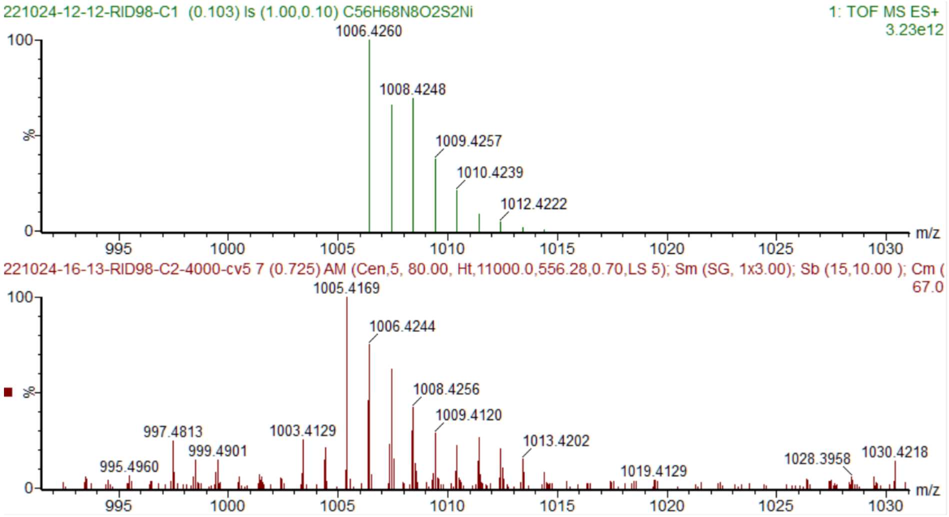

Figure S30 The theoretical (above) and measured (below) isotopic distribution of 2TU·Ni(OAc)$_2$

5 Chiral HPLC profiles

5.1 Michael adduct (8)

Parameters of the measurements: Phenomenex Lux® 5 µm, Cellulose-1 column (250 × 4.6 mm ID, eluent hexane:ethanol = 85:15 with a flow rate of 0.8 mL·min^{-1}, UV detector α = 254 nm), 5 mL or 10 mL injection, 20 °C. Retention time for (*S*)-**8**: 16.5 min, for (*R*)-**8**: 18.1 min.

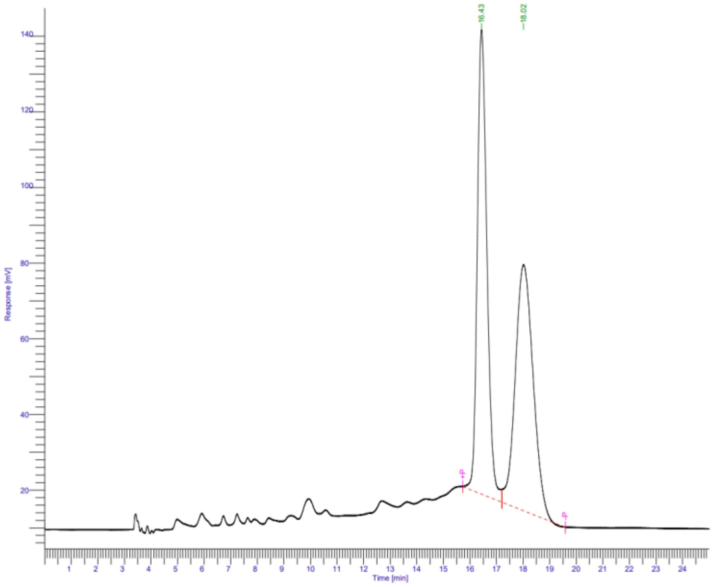

Peak #	Time [min]	Area [µV·s]	Height [µV]	Area [%]	Norm. Area [%]	BL	Area/Height [s]
1	16.434	3088585.90	122787.50	50.46	50.46	BV	25.1539
2	18.018	3032870.06	64993.53	49.54	49.54	*VB	46.6642
		6121455.96	187781.03	100.00	100.00		

Figure S31. HPLC chromatogram of racemic **9**

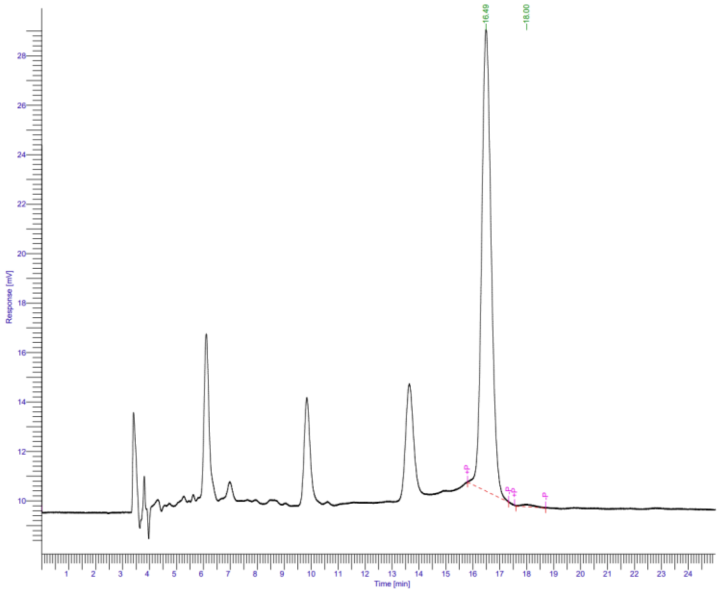

Peak #	Time [min]	Area [µV·s]	Height [µV]	Area [%]	Norm. Area [%]	BL	Area/Height [s]
1	16.490	459286.38	18648.99	99.45	99.45	*BB	24.6279
2	17.996	2517.42	87.76	0.55	0.55	*BB	28.6841
		461803.80	18736.76	100.00	100.00		

Figure S32. HPLC chromatogram of enantiomeric enriched **9** (Table 1, Entry 1)

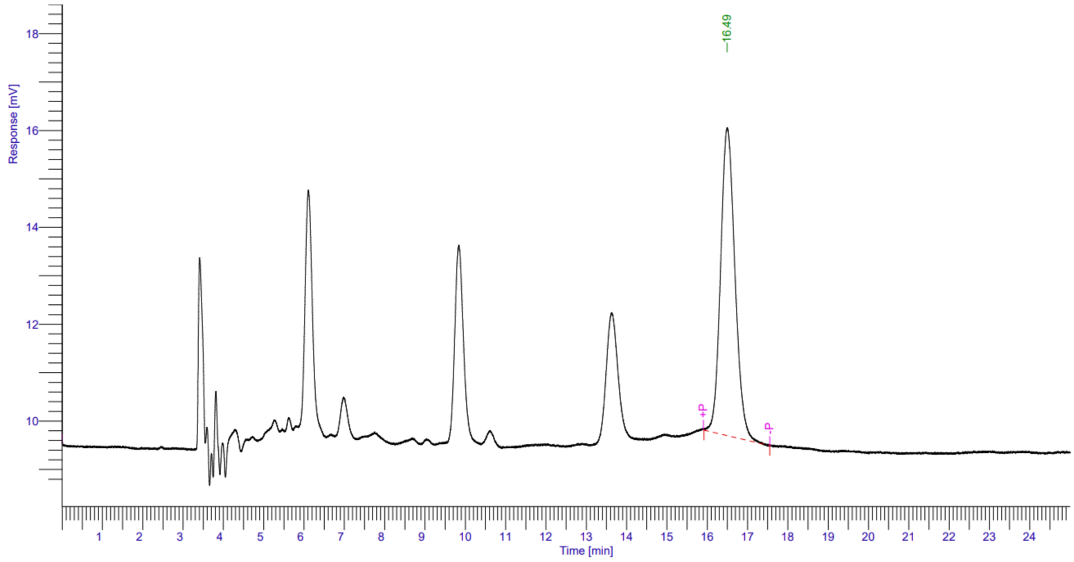

Peak #	Time [min]	Area [µV·s]	Height [µV]	Area [%]	Norm. Area [%]	BL	Area/Height [s]
1	16.492	157557.92	6357.21	100.00	100.00	*BB	24.7841
		157557.92	6357.21	100.00	100.00		

Figure S33. HPLC chromatogram of enantiomeric enriched **9** (Table 1, Entry 2)

5.2 Friedel–Crafts adduct (11)

Phenomenex Lux® 5 µm, Cellulose-1 column (250 × 4.6 mm ID, a mixture of water (0.1% NH_4OAc):MeCN = 40:60 as the eluent with a flow rate of 0.8 mL·min^{-1}, UV detector α = 254 nm); UV detector 254 nm, 5 mL or 10 mL injection, 20 °C. Retention time for (*S*)-**11**: 12.3 min, for (*R*)-**11**: 13.1 min.

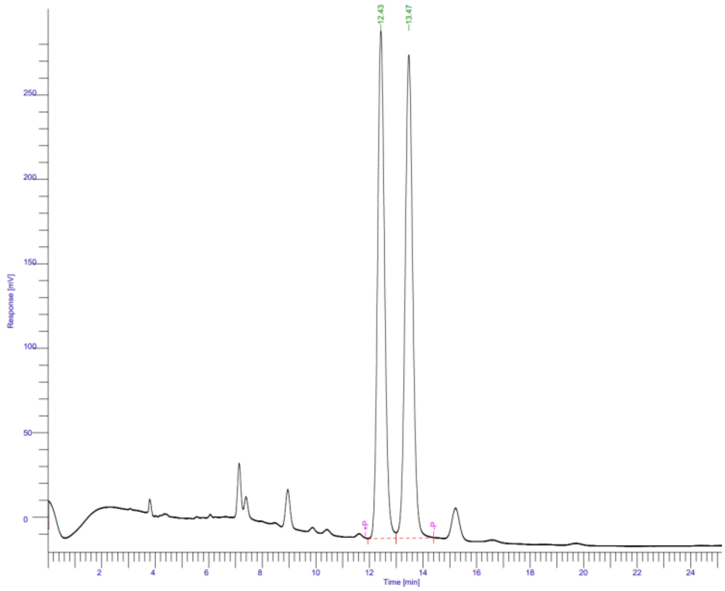

Peak #	Time [min]	Area [µV·s]	Height [µV]	Area [%]	Norm. Area [%]	BL	Area/Height [s]
1	12.426	5506329.97	300663.52	49.11	49.11	BV	18.3139
2	13.470	5705822.93	285964.91	50.89	50.89	*VB	19.9529
		11212152.89	586628.43	100.00	100.00		

Figure S34. HPLC chromatogram of racemic **11**

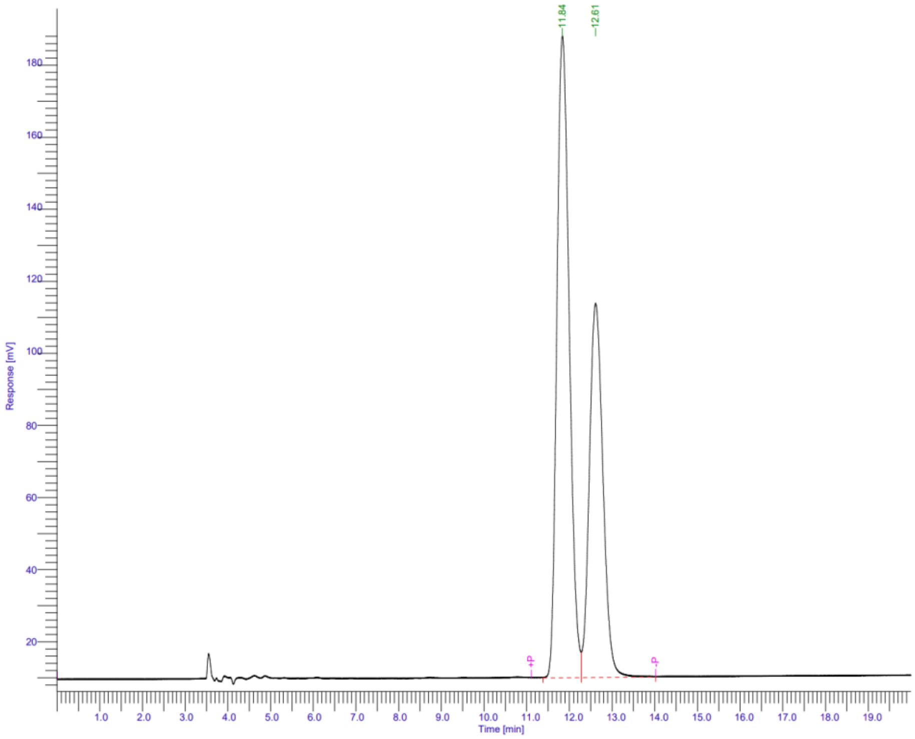

Peak #	Time [min]	Area [µV·s]	Height [µV]	Area [%]	Norm. Area [%]	BL	Area/Height [s]
1	11.837	3589416.18	178018.36	61.01	61.01	BV	20.1632
2	12.614	2293672.89	103716.83	38.99	38.99	*VB	22.1148
		5883089.07	281735.20	100.00	100.00		

Figure S35. HPLC chromatogram of enantiomeric enriched **11** (Table 2, Entry 2)

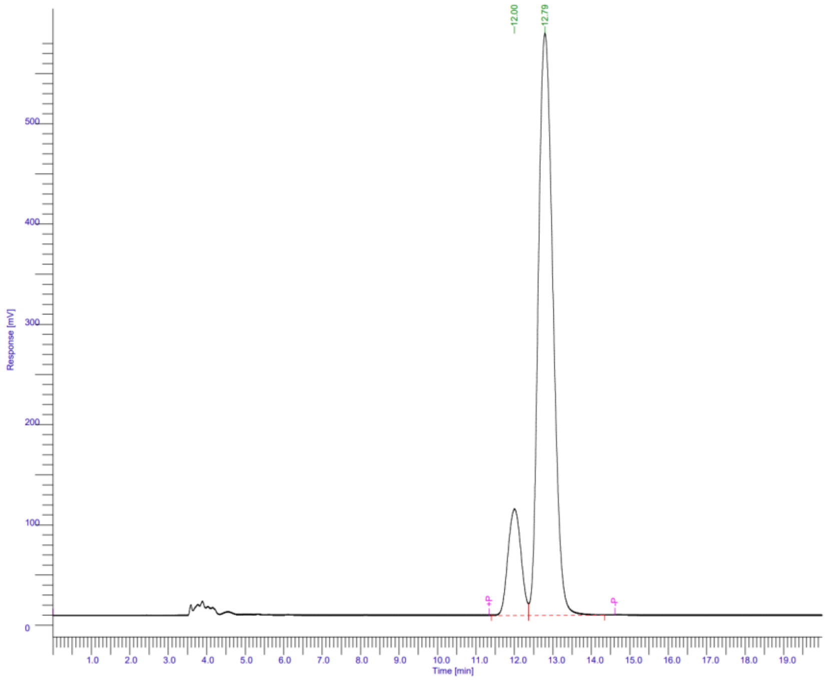

Figure S36. HPLC chromatogram of enantiomeric enriched **11** (Table 2, Entry 6)

5.3 A^3 adduct (15)

Kromasil® AmyCoat 5 µm, Cellulose-1 column (250 × 4.6 mm ID, a mixture of hexane:ethanol = 85:15 as the eluent with a flow rate of 0.8 mL·min^{-1}, UV detector α = 254 nm); UV detector 254 nm, 5 mL or 10 mL injection, 20 °C. Retention time for (*S*)-**15**: 8.5 min, for (*R*)-**15**: 9.7 min.

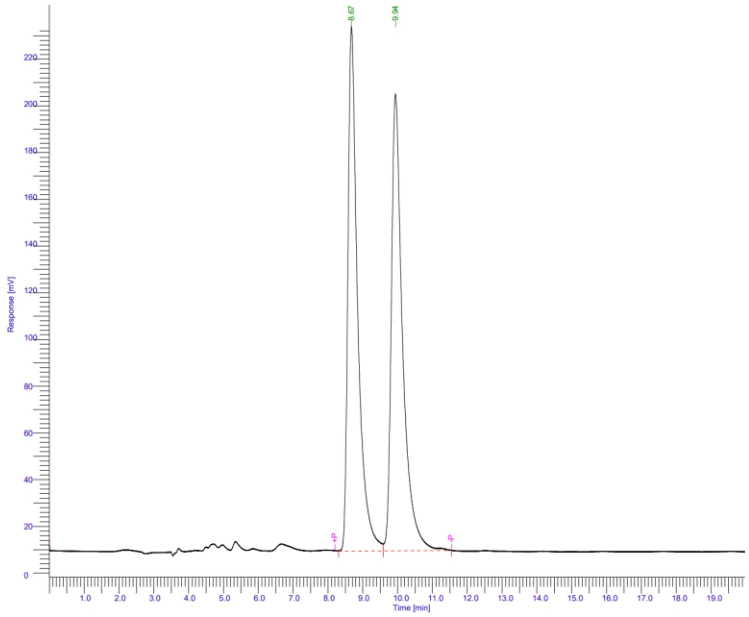

Peak #	Time [min]	Area [µV·s]	Height [µV]	Area [%]	Norm. Area [%]	BL	Area/Height [s]
1	8.673	4368972.29	224217.31	49.85	49.85	BV	19.4854
2	9.938	4394426.64	195365.11	50.15	50.15	*VB	22.4934
		8763398.92	419582.42	100.00	100.00		

Figure S37. HPLC chromatogram of racemic **15**

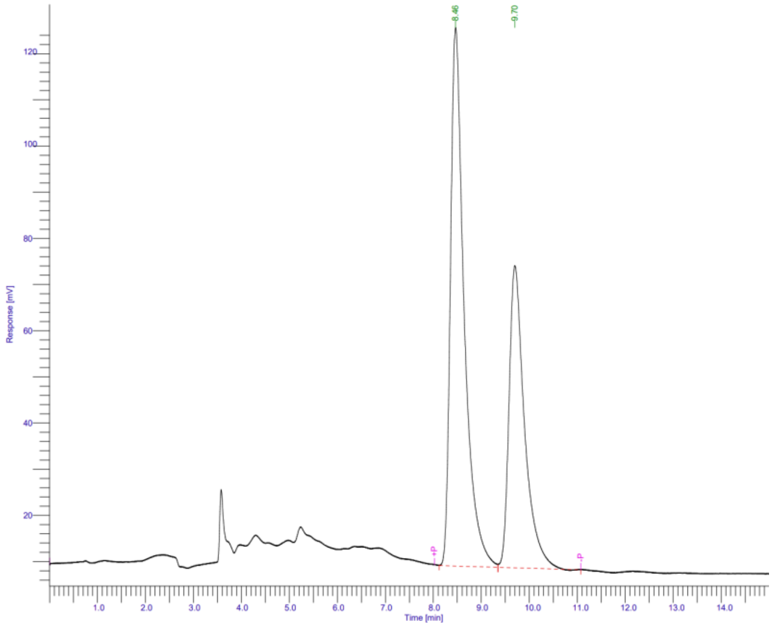

Peak #	Time [min]	Area [µV·s]	Height [µV]	Area [%]	Norm. Area [%]	BL	Area/Height [s]
1	8.464	2315880.54	116605.51	61.27	61.27	BV	19.8608
2	9.700	1463655.56	65426.47	38.73	38.73	*VB	22.3710
		3779536.11	182031.98	100.00	100.00		

Figure S38. HPLC chromatogram of enantiomeric enriched **15** (Table 3, Entry 3)

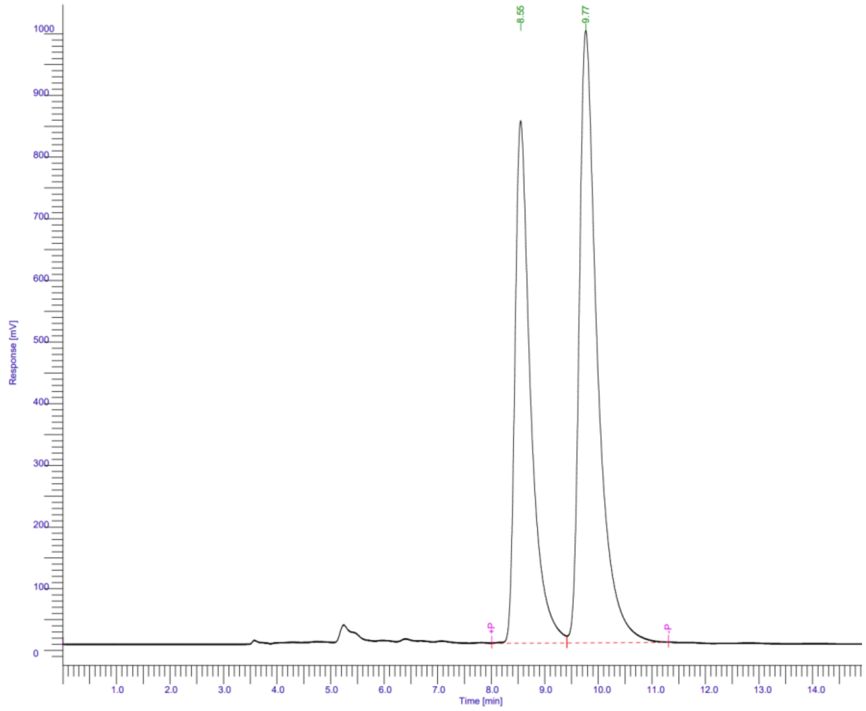

Peak #	Time [min]	Area [µV·s]	Height [µV]	Area [%]	Norm. Area [%]	BL	Area/Height [s]
1	8.549	17255528.79	846786.72	42.41	42.41	BV	20.3777
2	9.765	23432726.87	992686.49	57.59	57.59	*VB	23.6054
		40688255.66	1.84e+06	100.00	100.00		

Figure S39. HPLC chromatogram of enantiomeric enriched **15** (Table 3, Entry 12)

6 Quantum chemical computations

6.1. Complex formation with two ligands

6.1.1. S-Ni-S complex

First, we optimized the structure of Ni(II) complex with two thiosquaramide ligands, coordinating through the sulfur atoms (S-Ni-S complex). Several spin multiplicities were considered in the computations and their relative energies were compared to the most stable configuration are presented in Table S5 below. The computations show a triplet ground state, although singlet state only by 35 kJ·mol^{-1} higher in energy. As the higher spin states are considerably less stable, only the singlet and triplet states are discussed below.

Table S5. Relative energies of the different spin states of the Ni(TSQ)$_2$ complex. Method: B3LYP+D3(0)/def2-SVP.

**Spin multiplicity**	**Relative energy (kJ·mol^{-1})**
**1**	**35**
**3**	**0**
**5**	148
**7**	301

Singlet state:

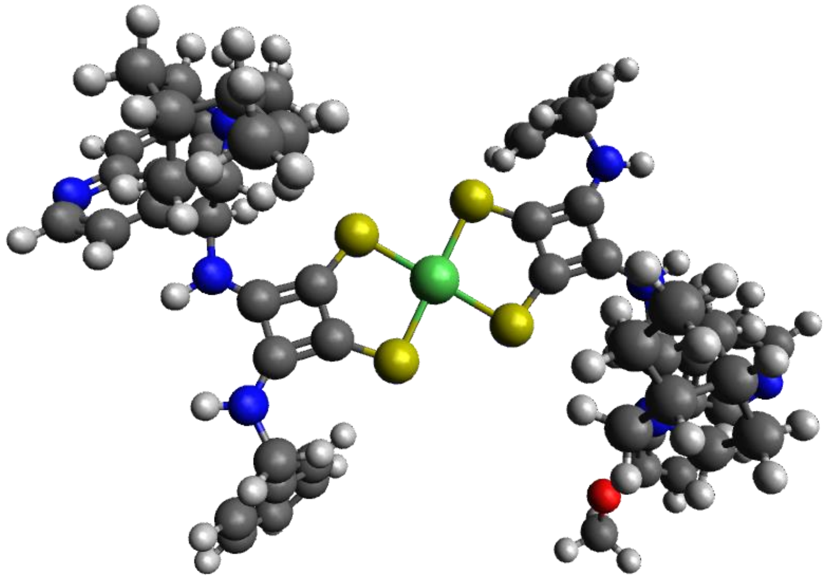

Figure S34. Optimized structure of S-Ni-S complex in singlet state.

In case of singlet state the original tetrahedral geometry of the ligands around the Ni(II) ion relaxes to square planar structure because of the Jahn-Teller distortion (Figure S34). The Ni-S Wiberg indices of 0.43 show no covalent bonds between nickel and sulfur atoms, while to Ni natural charge (+0.77) is significantly lower than the formal charge of +2.

The energy decomposition analysis shows high charge transfer energy (−175 kJ·mol^{-1}), and the analysis of the Complementary Occupied Virtual Pairs shows that the donation sulfur *p* electrons to a formally unoccupied *d* orbital of Ni(II) ion is responsible for the Ni-S interaction (Figure S35).

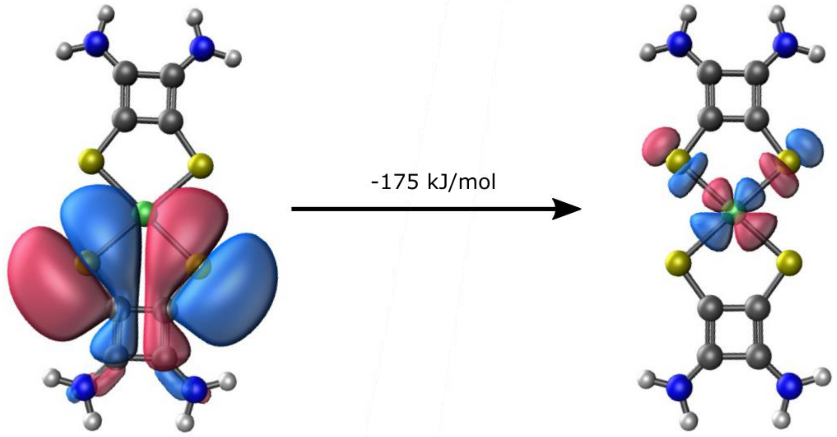

Figure S35. The donation of p orbitals on sulfur to a formally unoccupied d orbital of Ni(II) ion.

Triplet state:

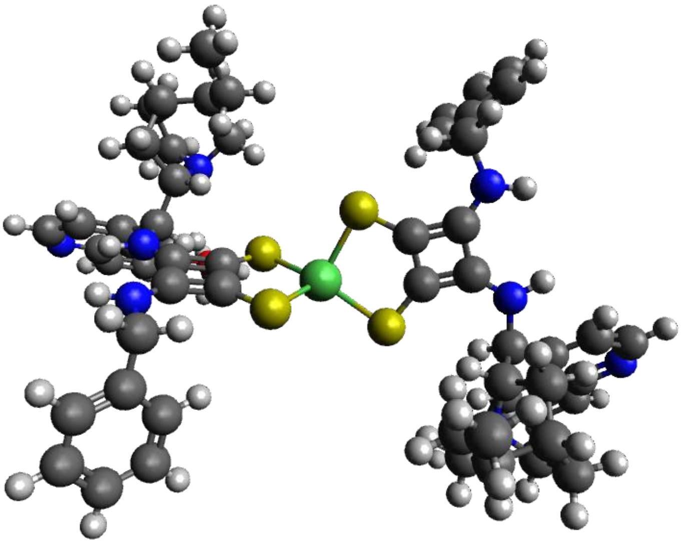

Figure S36. Optimized structure of S-Ni-S complex in triplet state. (Green: nickel, yellow: sulfur, blue: nitrogen, black: carbon, grey: hydrogen)

Tetrahedral geometry was observed in the case of higher spin states (Figure S36). In line, the NBO analysis shows that all nickel d-orbitals are (semi-)occupied in case of triplet state while there is a formally unoccupied d-orbital in singlet configuration. Furthermore, the Wiberg bond indeces of Ni-S are 0.29 and the natural charge of Ni is 1.07 which indicate a weaker interaction with the ligands.

6.1.2. N-Ni-S and N-Ni-N complexes

We also studied the complex formation with two more possible configurations in which one (N-Ni-S complex) or both (N-Ni-N complex) thiosquaramide ligands coordinate through the amino groups on the other side of the four membered ring.

In case of N-Ni-S complex (Figure S37) the proximity of the protective groups destabilizes the complex, the relative energy is +144 kJ·mol^{-1} comparing to that of the S-Ni-S complex. In the structure the nickel ion interacts with only one nitrogen atom, as it binds to one of the amino groups perpendicularly to the four membered ring plane. This leads to the distortion of the originally planar structure around the nitrogen atom, which shows decreased extend of electron delocalization. This reduced delocalization increases the energy, thus destabilizing the complex in this binding mode. In line, the complex formation with two nitrogen atoms of the ligand is unfavourable.

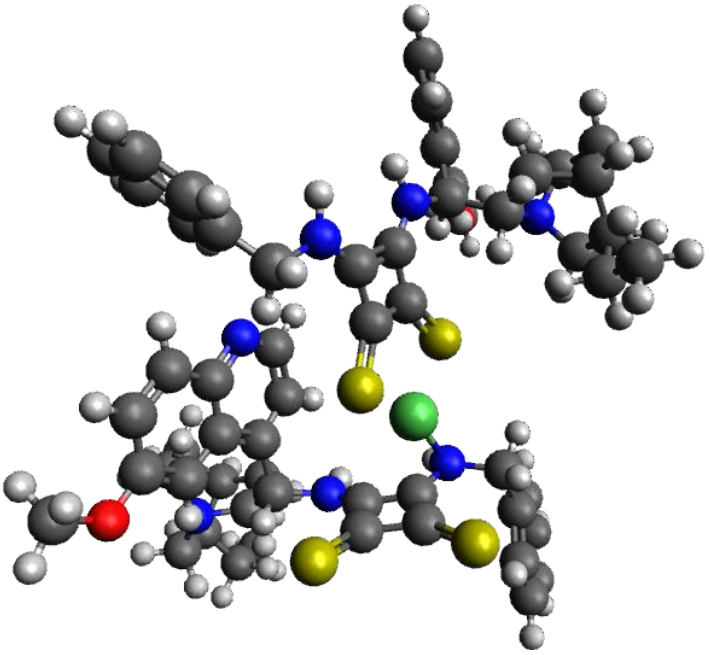

Figure S37. Optimized structure of the N-Ni-S complex (Green: nickel, yellow: sulfur, blue: nitrogen, black: carbon, grey: hydrogen)

In case of N-Ni-N complex (Figure S38) we observed similar structures. The proximity of the protective groups and the decreased delocalization around the nitrogen in the amino groups make this complex even less favorable. Although the complete optimization of the structure was not performed, our computations for preoptimizing the structures of complexes using semiempirical tight-binding method (GFN-xTB) showed significantly high energy comparing to the previous configurations.

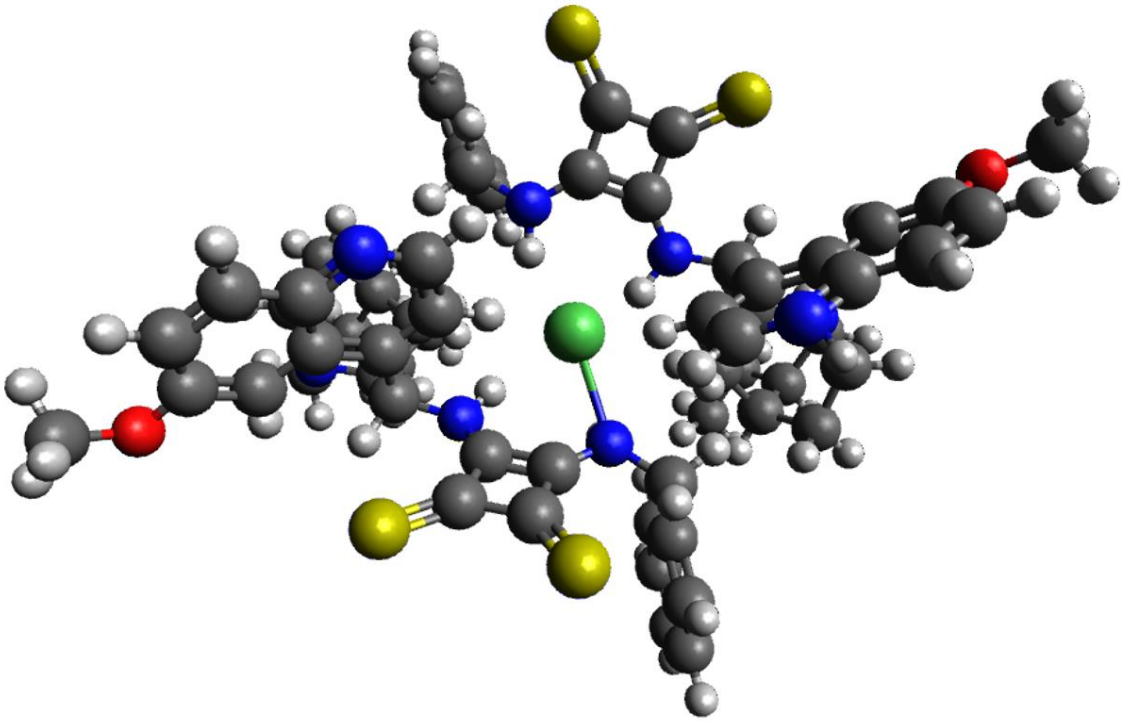

Figure S38. Preoptimized structure of the N-Ni-N complex. (Green: nickel, yellow: sulfur, blue: nitrogen, black: carbon, grey: hydrogen)

6.2. Complex formation with one ligand

We calculated the formation energy of the complex considering four structures with different ligand coordination to the nickel(II) ion as follows:

- S: Ligand binds with the thiosquaramide sulfur atoms
- N: Ligand binds with the thiosquaramide nitrogen atoms
- Lin: Ligand binds with the quinoline nitrogen atom
- Din: Ligand binds with the quinuclidine group nitrogen atom

Apart from the thiosquaramide ligand, an acetate ion, presenting in the experiments is also coordinated to the nickel ion.

The structures were optimized both in singlet and triplet spin states. Higher spin states were also considered, but the results indicate that higher multiplicities are energetically unfavourable comparing to singlet or triplet states. The formation energy was calculated as

$$E_{formation} = E_{complex} - (E_{ligand} + E_{Ni(II)ac})$$

where $E_{complex}$, E_{ligand} and $E_{Ni(II)ac}$ are the total energy of the ligand-Ni(II)-acetate complex, ligand and Ni(II)-acetate, respectively. The smaller the calculated energy is the more favorable the formation of the complex is. The results are shown in Table S6 and the optimized structures of the four complexes with both singlet and triplet states are also summarized in Figure S39-42.

Table S6. Formation energy of Ni(TSQ)Ac complex (TSQ: thiosquaramide, Ac: acetate). Method: B3LYP+D3(0)/def2-SVP.

	**Formation energy (kJ·mol^{-1})**	
	Singlet state	Triplet state
**S**	−511	−502
**N**	−348	−437
**Lin**	−496	−533
**Din**	−593	−579

According to the table, the most stable complex is the one in the Din configuration with a formation energy of −593 kJ·mol^{-1} in its singlet state. Furthermore, complex S (−511 and −502 kJ·mol^{-1}) and the triplet state of Lin (−533 kJ·mol^{-1}) also show significant stabilities. Although Ni(II) ion is located at different parts of the complex in these structures, the sulfur atoms of the ligands always coordinate to the Ni(II), similarly to that observed in the case of the complexes with two ligands..

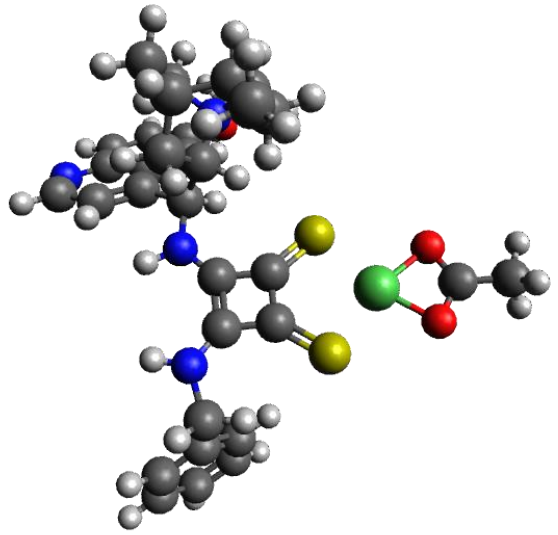

Figure S39. Structure of S-Ni-acetate complex in both singlet and triplet state. (Green: nickel, yellow: sulfur, blue: nitrogen, black: carbon, red: oxygen, grey: hydrogen)

a)

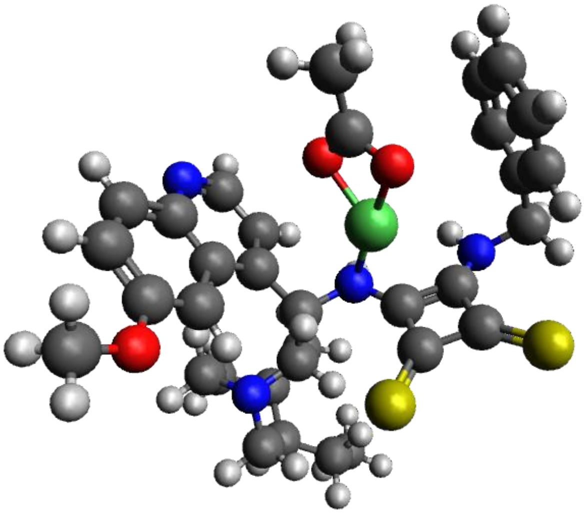

b)

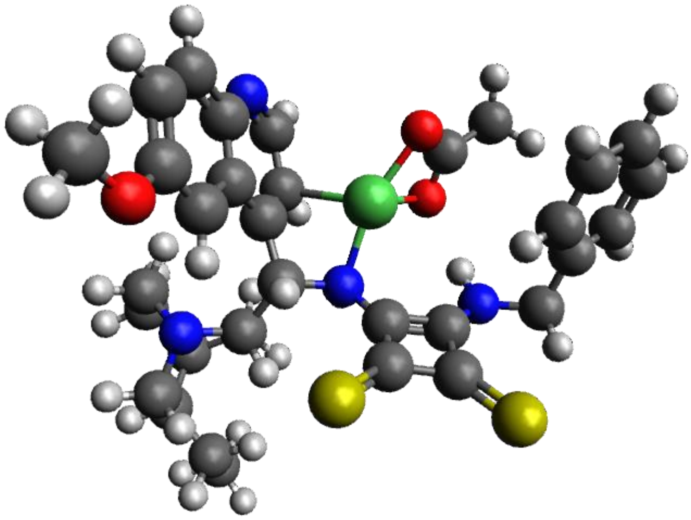

Figure S40. Structure of N-Ni-acetate complex in a) singlet and b) triplet state. (Green: nickel, yellow: sulfur, blue: nitrogen, black: carbon, red: oxygen, grey: hydrogen)

a)

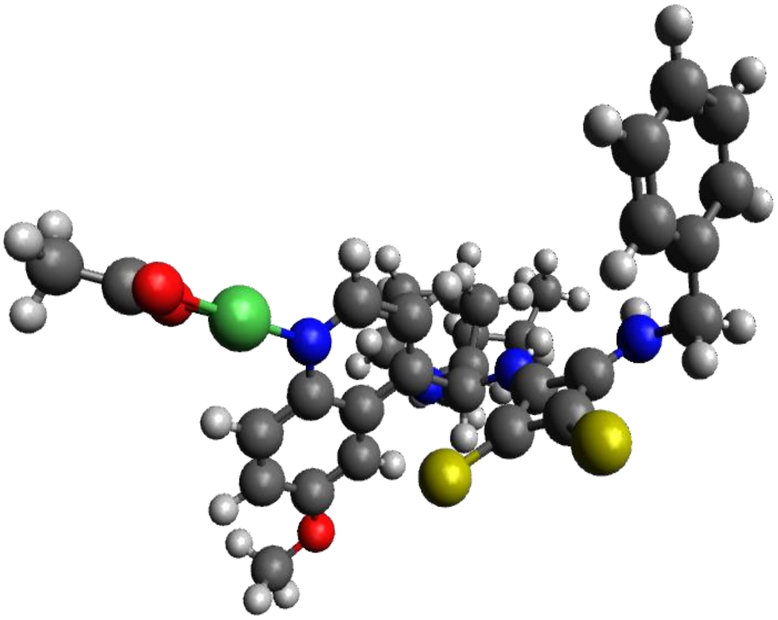

b)

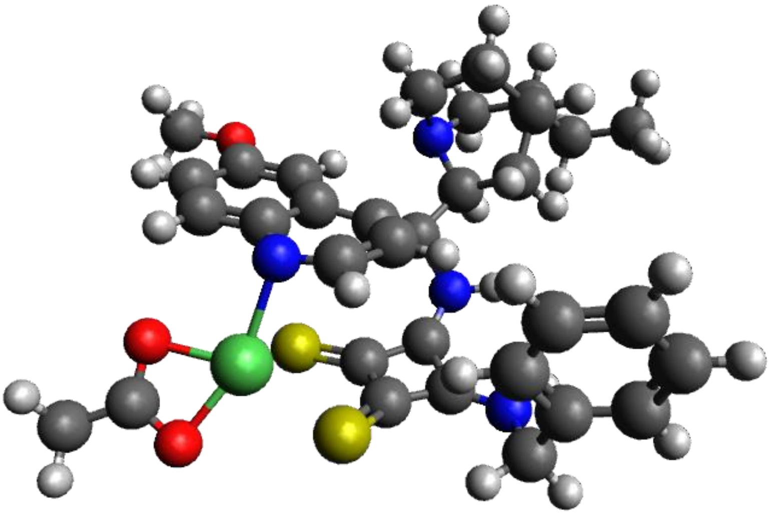

Figure S41. Structure of Lin-Ni-acetate complex in a) singlet and b) triplet state. (Green: nickel, yellow: sulfur, blue: nitrogen, black: carbon, red: oxygen, grey: hydrogen)

a)

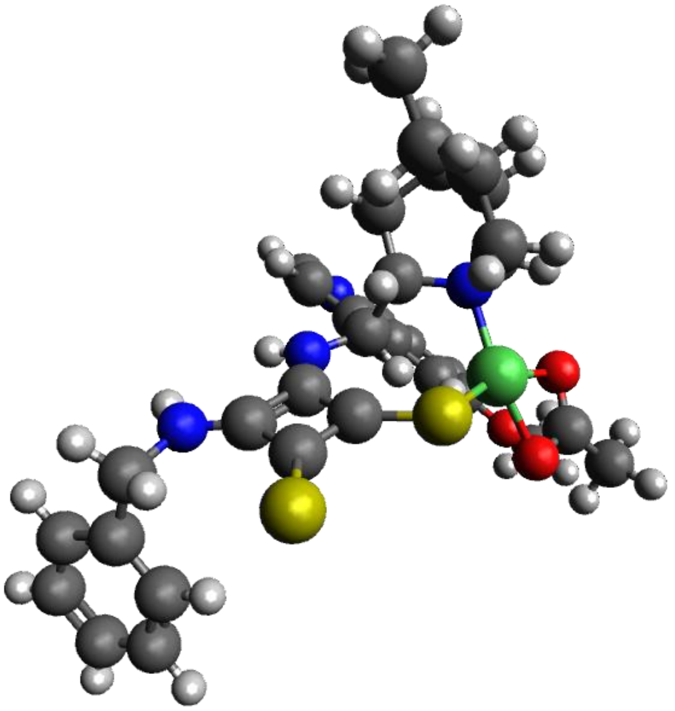

b)

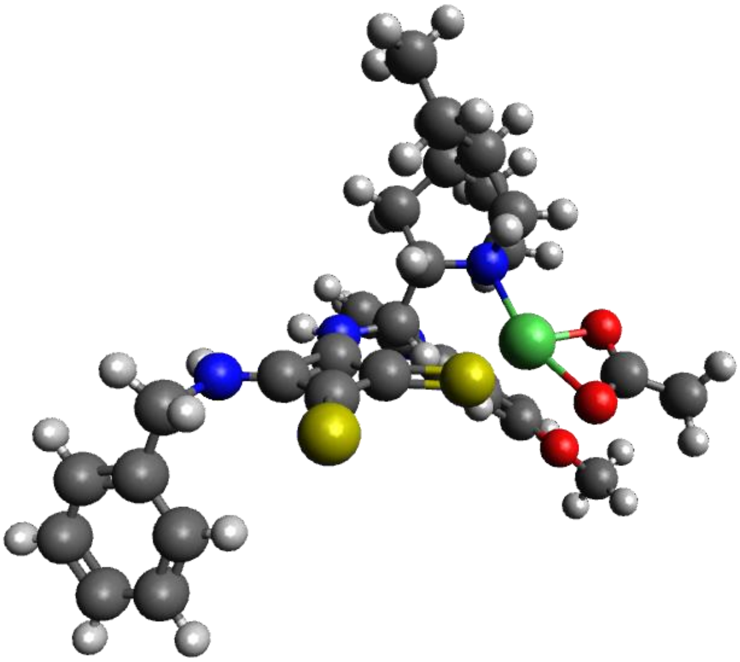

Figure S42. Structure of Din-Ni-acetate complex in a) singlet and b) triplet state. (Green: nickel, yellow: sulfur, blue: nitrogen, black: carbon, red: oxygen, grey: hydrogen)

6.3. Stability of Ni(II) complexes with one or two thiosquaramide ligands

Here we examine the stability of Ni(II) complexes having one or two thiosquaramide ligands. In the computations we considered an acetate ion in the initial (complex with one ligand) and final (complex with two ligands) structure too. The formation energy was calculated with the equation below

$$E_{formation} = E_{Ni(II)L2} - (E_{ligand} + E_{Ni(II)L})$$

where $E_{Ni(II)L2}$ and $E_{Ni(II)L}$ are the total energy of the complex with two and one ligand in S configuration, respectively. The optimized structure of the S-Ni-S complex with acetate ion is shown in Figure S43 and the associated formation energy is −254 kJ·mol^{-1} which suggests that the coordination of two thiosquaramide ligands is more favourable.

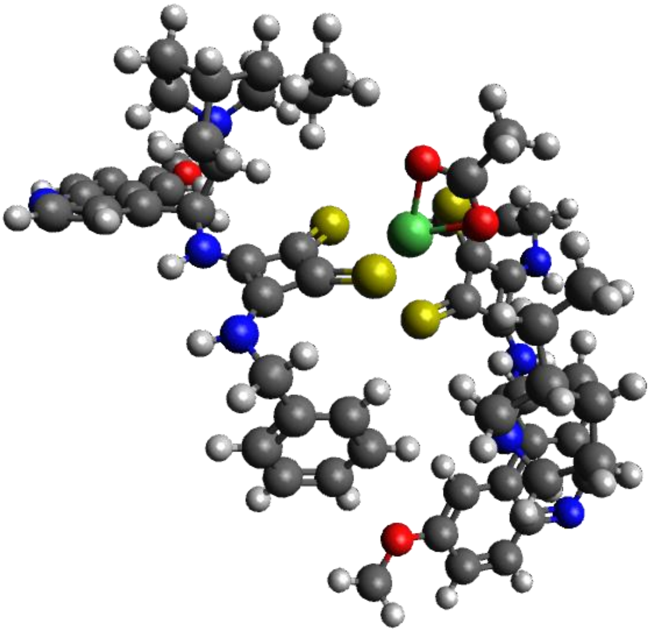

Figure S43. Structure of Ni-L$_2$-acetate complex (L: thiosquaramide). (Green: nickel, yellow: sulfur, blue: nitrogen, black: carbon, red: oxygen, grey: hydrogen)

6.4. Comparison of thiosquaramide (TSQ), thiourea (TU) and squaramide (SQ) ligands

Similar computations were performed considering squaramide and thiourea as ligands. The formation energy was calculated with the equation below,

$$E_{formation} = E_{complex} - (E_{ligand} + E_{Ni(II)ac})$$

where $E_{complex}$, E_{ligand} and $E_{Ni(II)ac}$ are the total energy of the ligand-Ni(II)-acetate complex, ligand and Ni(II)-acetate, respectively. The results are summarized in Table S7 and Figure S44-45.

Table S7. Formation energy of Ni(L)Ac complex (L: thiosquaramide (TSQ), thiourea (TU) or squaramide (SQ); Ac: acetate). Method: B3LYP+D3(0)/def2-SVP.

	Formation energy (kJ·mol^{-1})		
	**TSQ**	**TU**	**SQ**
**a) S or O**	−502	−464	−438
**b) N**	−437	–	−454
**c) Lin**	−533	−507	−526
**d) Din**	−579	−609	−610

a)

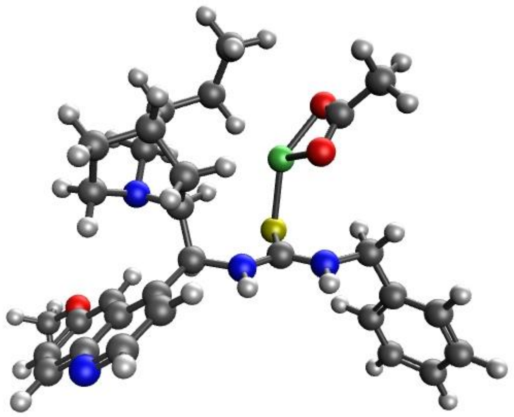

b)

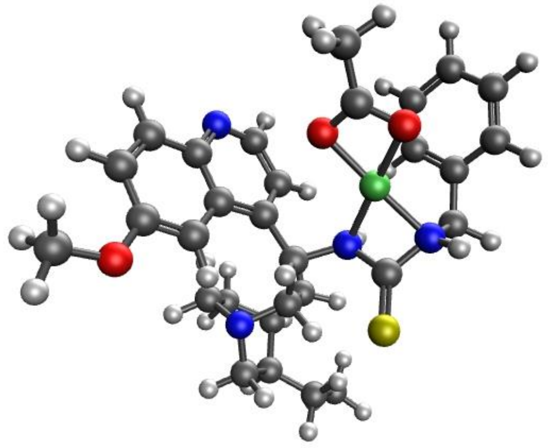

c)

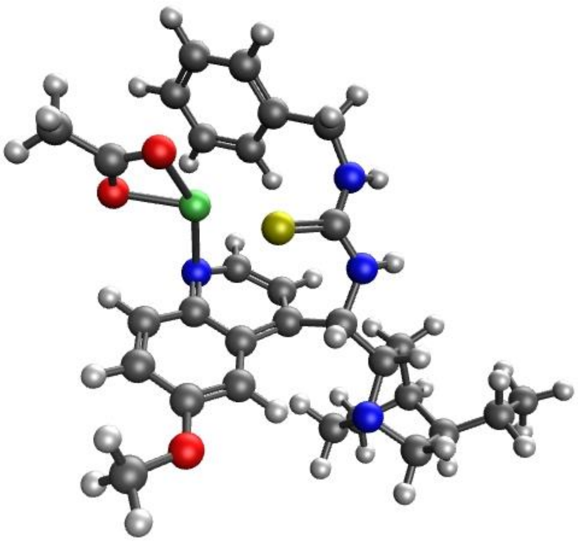

d)

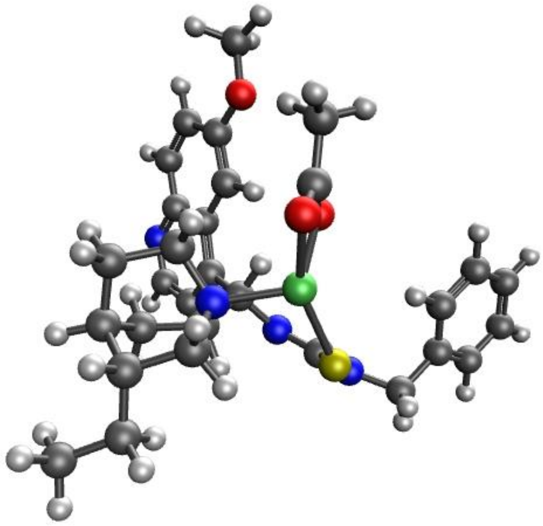

Figure S44. Structures of L-Ni(II)-acetate with thiourea (TU) ligand. (Green: nickel, yellow: sulfur, blue: nitrogen, black: carbon, red: oxygen, grey: hydrogen)

a)

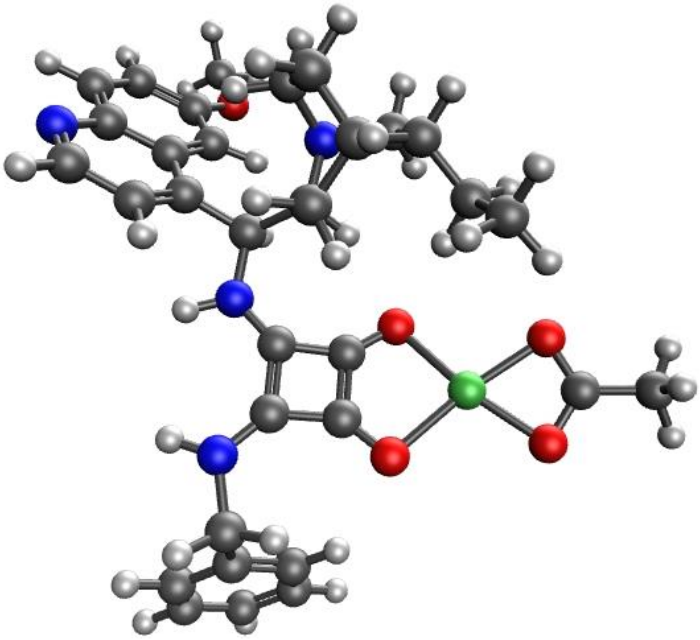

b)

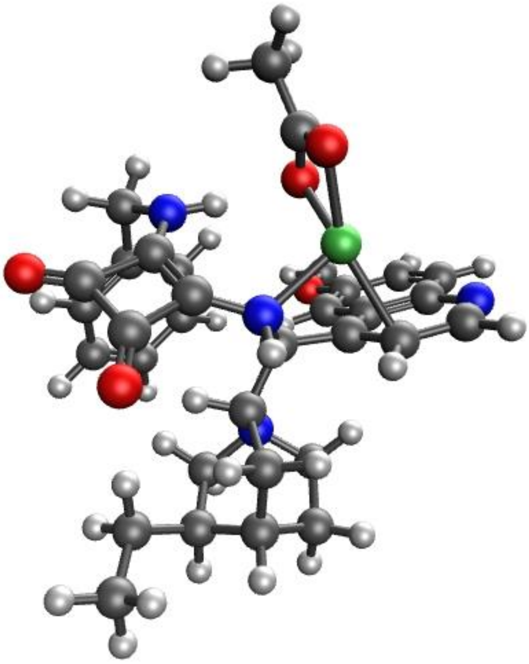

c)

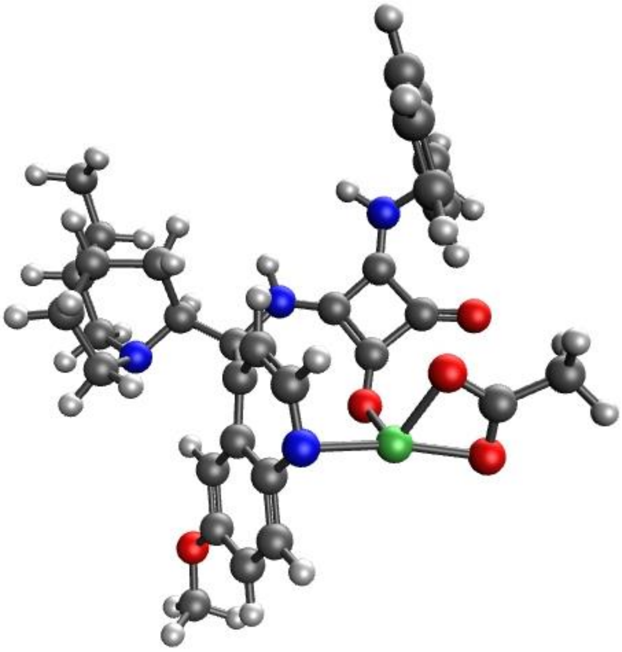

d)

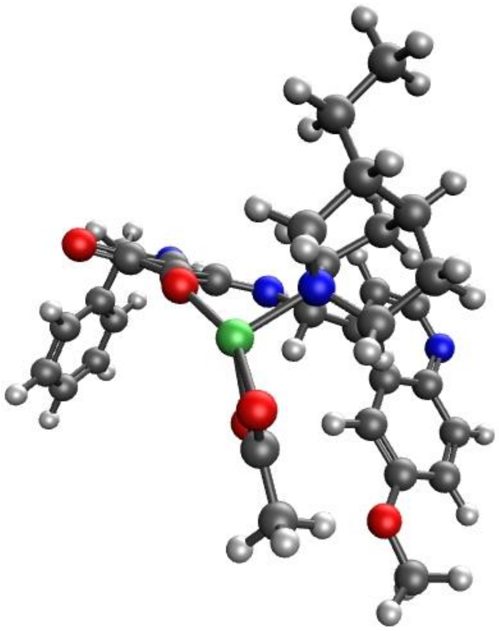

Figure S45. Structures of L-Ni(II)-acetate with squaramide (SQ) ligand. (Green: nickel, yellow: sulfur, blue: nitrogen, black: carbon, red: oxygen, grey: hydrogen)

6.5. Analysis of L-Ni(II)-Acetate complexes

6.5.1. Natural Bond Orbital (NBO) analysis

Table S8. Wiberg bond indexes in the optimized structure of L-Ni(II)-acetate (L: Squaramide, Thiosquaramide, Thiourea).

	Wiberg bond index
**Ni-O**	0.2 – 0.24
**Ni-S**	0.41 – 0.47
**Ni-N**	0.2 – 0.33

Table S9. Natural charges of the different atoms in the optimized structure of L-Ni(II)-acetate (L: Squaramide, Thiosquaramide, Thiourea).

		Natural charges				
		**Ni**	**O/S**	**O/S**	**N(lin)**	**N(din)**
**Squaramide**	**ligand**	–	−0.586	−0.586	−0.469	−0.571
	**o-ni-ac**	1.138	−0.656	−0.658	−0.448	−0.567
	**lin-ni-ac**	1.137	−0.707	−0.551	−0.575	−0.576
	**din-ni-ac**	1.108	−0.686	−0.536	−0.446	−0.580
**Thiosquaramide**	**ligand**	–	−0.070	−0.075	−0.469	−0.567
	**s-ni-ac**	0.942	−0.023	−0.034	−0.448	−0.572
	**lin-ni-ac**	1.031	0.034	−0.112	−0.562	−0.574
	**din-ni-ac**	1.000	−0.056	0.036	−0.446	−0.585
**Thiourea**	**ligand**	–	−0.281	–	−0.471	−0.569
	**s-ni-ac**	1.018	−0.267	–	−0.448	−0.570
	**lin-ni-ac**	1.081	−0.268	–	−0.577	−0.574
	**din-ni-ac**	1.019	−0.230	–	−0.446	−0.580

6.5.2. Energy decomposition analysis (EDA)

Table S10. Energy decomposition analysis on the optimized structure of L-Ni(II)-acetate (L: Squaramide, Thiosquaramide, Thiourea). Method: B3LYP+D3(0)/def2-SVP.

Energies [kJ·mol^{-1}]	L = Squaramide			
	S-Ni	N-Ni	Lin-Ni	Din-Ni
Elec.+Pauli	−123	113	−90	−68
Disp.	−44	−87	−51	−90
Frozen (Elec.+Pauli+Disp.)	−167	26	−141	−158
Polarization	−170	−249	−223	−240
Charge Transfer	−179	−284	−203	−261
Total	−517	−508	−567	−659

Energies [kJ·mol^{-1}]	L = Thiosquaramide			
	S–Ni	N–Ni	Lin–Ni	Din–Ni
Elec.+Pauli	−77	90	−77	−35
Disp.	−39	−85	−62	−95
Frozen (Elec.+Pauli+Disp.)	−116	6	−140	−130
Polarization	−197	−192	−231	−235
Charge Transfer	−247	−219	−249	−296
Total	−560	−406	−619	−659

Energies [kJ·mol^{-1}]	L = Thiourea			
	S-Ni	N-Ni	Lin-Ni	Din-Ni
Elec.+Pauli	−2	35	−64	−75
Disp.	−69	−88	−83	−80
Frozen (Elec.+Pauli+Disp.)	−71	−54	−147	−155
Polarization	−162	−208	−192	−232
Charge Transfer	−194	−249	−228	−288
Total	−427	−510	−567	−675

Table S11. Energy decomposition analysis on the optimized structure of L_2-Ni(II) (L: Squaramide, Thiosquaramide, Thiourea).

Energies [kJ·mol^{-1}]	Ni-L_2 complex	
	SQ	TSQ
Elec.+Pauli	−187	−92
Disp.	−25	−24
Frozen (Elec.+Pauli+Disp.)	−212	−115
Polarization	−209	−214
Charge Transfer	−231	−289
Total	−652	−618

6.6. Complex formation with one ligand in different solvents

The formation energy of the squaramide, thiosquaramide and thiourea complexes was calculated considering different solvents. The computations were performed using the B3LYP-D3(0)/def2-SVP level of theory, in conjunction with the SM12 implicit solvation model. The solvation energies were computed on the gas phase optimized geometry. Six different solvents were considered: dimethyl sulfoxide (DMSO), acetonitrile, methanol, dichloromethane (DCM), tetrahydrofuran (THF) and toluene. The formation energy was calculated with the equation below

$$E_{formation} = (E_{complex} + E_{ac}) - (E_{ligand} + E_{Ni(II)(ac)2})$$

where $E_{complex}$, E_{ligand} and $E_{Ni(II)ac}$ are the total energy of the ligand-Ni(II)-acetate complex, ligand and Ni(II)-acetate, respectively.

Table S12. Formation energy of L-Ni(II)-acetate (L: Squaramide, Thiosquaramide, Thiourea) in six different solvents. Method: B3LYP+D3(0)/def2-SVP + SM12 implicit solvation

Formation energy [kJ·mol^{-1}]		DMSO	Acetonitrile	Methanol	DCM	THF	Toluene	Gas phase
SQ	O	136	121	71	158	179	300	524
	N	154	148	112	184	202	306	513
	Lin	118	116	78	147	162	267	478
	Din	6	2	−40	34	49	157	367
TSQ	S	67	65	24	98	114	224	442
	N	240	230	191	267	284	392	605
	Lin	110	109	67	141	154	258	457
	Din	3	2	−42	34	48	155	361
TU	S	115	124	68	144	165	299	561
	N	235	230	194	263	281	394	567
	Lin	81	79	36	113	127	238	495
	Din	−21	−22	−63	11	26	135	374

6.7. Data of optimized structures

6.7.1. Basic structures

System: Acetate ion
Total energy [Ha]: -228.334877632749
Number of imaginary frequencies: 0
Multiplicity: 1
Charge: -1

C 1.3613708745 0.0192455017 -0.0001345723
C -0.2192896711 0.0054388422 -0.0016823754
H 1.7691407536 1.0439482866 -0.0396962218
H 1.7408100449 -0.5647165244 -0.8589644712
H 1.7347849057 -0.4889480055 0.9084387248
O -0.7228099745 -1.1399307826 0.0042041647
O -0.7727332992 1.1262549836 -0.0041894413

System: Ni - Acetate
Total energy [Ha]: -1736.17670033745
Number of imaginary frequencies: 0
Multiplicity: 1
Charge: +1

C -2.5042663212 -0.0072192141 0.0068021747
C -1.0346876387 -0.0049527070 -0.0043546193
H -2.8842715107 -1.0363492590 -0.0334107154
H -2.8706505043 0.5813868662 -0.8507317584
H -2.8512375625 0.4986052728 0.9239944866
O -0.2999489609 1.0539003999 -0.0067272541
O -0.2939111711 -1.0572980168 -0.0046293070
Ni 1.1901279839 0.0029987113 -0.0023329655

System: Ni - Acetate
Total energy [Ha]: -1736.19331980866
Number of imaginary frequencies: 0
Multiplicity: 3
Charge: +1

C -2.4859093815 -0.0068461952 0.0030882010
C -1.0079392034 -0.0065809194 -0.0032537143
H -2.8715173958 -1.0326475110 -0.0390309798
H -2.8467179113 0.5857646613 -0.8531604582
H -2.8334833306 0.5010479636 0.9181532147
O -0.3062474684 1.0739974830 -0.0030173710

O -0.2985872517 -1.0799124325 -0.0027043694
Ni 1.2733492332 0.0045328524 -0.0012082347

System: Ni-(Acetate)$_2$
Total energy [Ha]: -1964.87474205548
Number of imaginary frequencies: 0
Multiplicity: 1
Charge: 0

C -3.7370567078 0.0150485043 0.2509152863
C -2.2426808945 0.0139436194 0.1958467714
H -4.1216426998 -1.0118603454 0.2267274847
H -4.1324567552 0.5948584179 -0.5968049145
H -4.0620229144 0.5206766485 1.1734541875
O -1.5672059796 1.0928602405 0.1705729490
O -1.5644281602 -1.0626900418 0.1793722397
Ni 0.0171815870 0.0154216583 0.1441352239
C 2.2770418294 0.0194375559 0.0913893720
O 1.5978403731 1.0953244602 0.1053118205
O 1.6025120750 -1.0602506359 0.1169252677
C 3.7718599209 0.0175657013 0.0494906041
H 4.1553908385 1.0446041093 0.0152155305
H 4.1083864770 -0.5471104853 -0.8333942613
H 4.1575992052 -0.5045335424 0.9385825241

System: Ni-(Acetate)$_2$
Total energy [Ha]: -1964.873827916044
Number of imaginary frequencies: 0
Multiplicity: 3
Charge: 0

C -3.8465998070 -0.0052001450 0.0107694365
C -2.3462678310 -0.0091112965 -0.0040343410
H -4.2389462335 -1.0284770206 -0.0265145092
H -4.2167611367 0.5806282745 -0.8441015134
H -4.1938909893 0.5012165448 0.9246208983
O -1.6931048660 1.0829050230 -0.0050246926
O -1.6860888817 -1.0956931192 -0.0077722793
Ni -0.0000042629 -0.0008276679 -0.0007235354
C 2.3462645512 0.0088680733 0.0022356903
O 1.6853665080 1.0950032471 0.0039911297
O 1.6938074682 -1.0835860399 0.0028615205
C 3.8466799770 0.0061366763 -0.0014980976
H 4.2376485114 1.0304479075 -0.0203030998
H 4.2042618757 -0.5567597121 -0.8771091726

H 4.2081696311 -0.5235562264 0.8932359068

6.7.2. Structures of ligands

System: Squaramide
Total energy [Ha]: -1645.30106945235
Number of imaginary frequencies: 0
Multiplicity: 1
Charge: 0

C -1.6284354377 0.8822383334 -0.1436906056
C -2.9903882652 1.0116254386 -0.4686590284
C -3.2267177000 2.0200910922 0.5939274865
C -1.7351050486 1.9042720371 0.9455391155
O -0.9790597767 2.4129859497 1.7434913602
O -4.1972138451 2.6203618123 1.0017276100
N -0.6703278924 0.0964716306 -0.6757561117
N -3.7933843582 0.4658414240 -1.3973172733
C 0.7069425829 -0.0845662981 -0.1874151154
H -0.9950329980 -0.6463039983 -1.2857036593
C 1.4090855819 -1.0692805108 -1.1068151042
C 2.3267470187 -2.0466847225 -0.5864493631
C 2.8850462760 -2.9884980401 -1.5210165113
N 2.6188398444 -2.9642907103 -2.8567477845
C 1.8117324933 -2.0326299404 -3.3040653200
C 1.1792127148 -1.0721616226 -2.4711696805
C 1.3961020908 1.2987650849 0.0018181358
H 0.6697941071 -0.5326345785 0.8173661720
N 2.7656862810 1.2024472895 0.5342602992
C 1.3404056169 2.2673977531 -1.2051827046
H 0.8180557344 1.7551595990 0.8134517218
C 2.5999163738 3.1566014987 -1.1281126197
C 3.7783926601 1.0400465017 -0.5241013598
C 3.8130439285 2.2757247108 -1.4710498594
H 3.5641075603 0.1264558244 -1.0882384344
H 4.7512982243 0.8790813009 -0.0337572348
H 3.7712949414 1.9608801072 -2.5269807322
H 4.7438799634 2.8531364072 -1.3447535839
C 2.8002162335 3.7060558874 0.3089606439
H 2.5239982657 3.9873109156 -1.8456484462
C 3.0399585318 2.4653366818 1.2403963443
H 0.4187828324 2.8681811655 -1.1710784037
H 1.3377277262 1.7310975159 -2.1684417760
H 2.3917933764 2.5194147258 2.1292447640
H 4.0806739194 2.4346011715 1.5995460152

C -5.2549185074 0.6117033299 -1.3378669374
H -3.4415217109 -0.3228790832 -1.9312194686
C -5.9145459613 -0.4586263005 -0.4922859338
H -5.6391379939 0.5836252642 -2.3692111200
H -5.4612606110 1.6076738116 -0.9229928714
C -6.0020610216 -0.2961896823 0.8998144740
C -6.5484128680 -1.3080499322 1.6932141424
C -7.0123778581 -2.4909169108 1.1066031866
C -6.9340025314 -2.6567582029 -0.2793823079
C -6.3883032232 -1.6434589309 -1.0735397292
H -6.3366577205 -1.7729826155 -2.1595755069
H -5.6446715990 0.6334910981 1.3517360741
H -6.6166394195 -1.1707435329 2.7755471205
H -7.4405988590 -3.2806967040 1.7292527352
H -7.3034096798 -3.5747924115 -0.7439001043
C 1.6632403887 4.6072156089 0.8202923216
H 3.7199418578 4.3186486392 0.2878423163
C 1.3636548123 5.8158972998 -0.0668257273
H 1.9429322865 4.9578691179 1.8300166455
H 0.7447578380 4.0154932355 0.9672401084
H 2.2719319174 6.4144377122 -0.2551794749
H 0.6188572236 6.4772055214 0.4036639258
H 0.9588268682 5.5146705492 -1.0466119828
H 1.6191027921 -2.0145301764 -4.3844058482
H 0.5059950550 -0.3420054430 -2.9252851322
C 3.7710780857 -3.9843298899 -1.0339204753
C 4.1226325897 -4.0470790908 0.2976954248
C 3.6024358732 -3.0900298213 1.2126977513
C 2.7216868676 -2.1123331652 0.7704199782
H 2.3940869064 -1.3622477924 1.4883552773
H 4.1720357290 -4.6952862519 -1.7589373374
H 4.8067647188 -4.8262095669 0.6341866414
O 3.9217562076 -3.0604372951 2.5295635405
C 4.8350893381 -3.9992478348 3.0535765902
H 4.9439097995 -3.7645192631 4.1210617391
H 5.8266658835 -3.9264228248 2.5705088229
H 4.4659179541 -5.0365724612 2.9544334209

System: Squaramide
Total energy [Ha]: -1645.20792470300
Number of imaginary frequencies: 0
Multiplicity: 3
Charge: 0

C -1.5707456219 0.8127053321 -0.1701042002

C -2.9547334647 1.0128707617 -0.6752718886
C -3.2203698530 1.8724071698 0.5680917923
C -1.7391478018 1.6500287602 0.9984184879
O -1.0113190921 2.1895639424 1.8458067223
O -4.1872375046 2.4207570408 1.0194848584
N -0.6107838693 0.0733254518 -0.7229164105
N -3.7573834562 -0.0177209520 -1.1096614688
C 0.7882375064 -0.0620939180 -0.2851550575
H -0.8944362086 -0.5237621316 -1.4945449102
C 1.5192225987 -0.9545669672 -1.2716308959
C 2.4896252190 -1.9153054170 -0.8223954474
C 3.0704734928 -2.7772810476 -1.8181849109
N 2.7767083608 -2.6913140192 -3.1453167622
C 1.9225149158 -1.7726462454 -3.5267724564
C 1.2661574889 -0.8882641346 -2.6302263397
C 1.3996215654 1.3462186076 -0.0184370787
H 0.7810738915 -0.5640356998 0.6942803973
N 2.7621453936 1.2904889284 0.5338998255
C 1.3120102571 2.3665789854 -1.1780938297
H 0.7830409574 1.7298569771 0.8030363271
C 2.5182340430 3.3182498330 -1.0266766593
C 3.7996377615 1.2378021579 -0.5102012137
C 3.7839039641 2.5230182521 -1.3912923390
H 3.6440891516 0.3447508758 -1.1251852450
H 4.7711227604 1.1023304229 -0.0095569670
H 3.7772749506 2.2634581679 -2.4627857037
H 4.6796264566 3.1415841930 -1.2155289546
C 2.6608895595 3.7999776662 0.4412406824
H 2.4093727824 4.1807427451 -1.7011166999
C 2.9530757855 2.5267721682 1.3124201918
H 0.3569632674 2.9113073226 -1.1346373336
H 1.3621511537 1.8818025059 -2.1672856889
H 2.2838735645 2.4989195253 2.1862983550
H 3.9867717096 2.5329234973 1.6926344844
C -5.1803060736 0.1855590101 -1.3777133805
H -3.4970961328 -0.9787432918 -0.8770428642
C -6.0557387250 -0.6356932022 -0.4551558090
H -5.3925171231 -0.0769439707 -2.4302399207
H -5.3803609089 1.2604930557 -1.2596134643
C -6.3412543517 -0.1859209852 0.8453533481
C -7.1006767411 -0.9755727039 1.7115471141
C -7.5823431581 -2.2215816004 1.2933748376
C -7.3051806200 -2.6744292166 0.0005566324
C -6.5465071864 -1.8833455904 -0.8678296178
H -6.3375093322 -2.2372321071 -1.8823163459

H -5.9655441887 0.7871490298 1.1708101880
H -7.3206392028 -0.6142811461 2.7194693785
H -8.1773676121 -2.8363159106 1.9737984697
H -7.6835244954 -3.6435664985 -0.3351219116
C 1.4638169627 4.6060347703 0.9750348229
H 3.5442499049 4.4633514674 0.4724732280
C 1.0998242102 5.8351380105 0.1421482409
H 1.7095681930 4.9247412060 2.0040326922
H 0.5814231237 3.9528581589 1.0831856368
H 1.9719238751 6.4949363253 -0.0086942500
H 0.3133281356 6.4276530359 0.6359338409
H 0.7199638956 5.5574718375 -0.8545514990
H 1.7081394557 -1.7029987745 -4.6007477531
H 0.5575879825 -0.1632265859 -3.0378902548
C 4.0099745974 -3.7565144987 -1.4030355242
C 4.3912536740 -3.8748923457 -0.0835045983
C 3.8484559140 -2.9935784390 0.8925558013
C 2.9143558916 -2.0356017609 0.5214762442
H 2.5711189913 -1.3378800471 1.2841341637
H 4.4277044087 -4.4074776198 -2.1733939649
H 5.1171014254 -4.6382619936 0.1965725216
O 4.1964305952 -3.0183098124 2.2014124301
C 5.1544690931 -3.9472356977 2.6597490583
H 5.2772595161 -3.7615040068 3.7352822233
H 6.1323012402 -3.8136131720 2.1619255057
H 4.8209861475 -4.9913751077 2.5159606828

System: Thiourea
Total energy [Ha]: -1778.85345182523
Number of imaginary frequencies: 0
Multiplicity: 1
Charge: 0

C -2.0863124294 -0.6613862098 -0.2020375453
S -1.9548963295 -0.6733028252 -1.8818618636
N -1.0483135735 -0.3406506196 0.6217004607
N -3.2331476752 -0.9823349213 0.4678496803
C 0.3096535023 -0.0492027733 0.1727318304
H -1.1669677515 -0.5392576696 1.6085556241
C 0.9767732728 0.9705127334 1.0764410137
C 1.8644389830 1.9593854693 0.5242905060
C 2.4257518470 2.9264137043 1.4301826536
N 2.1802499278 2.9254645633 2.7703434791
C 1.3888416492 1.9946699487 3.2457951756
C 0.7640997416 1.0051240859 2.4415257724

C 1.0570514010 -1.3961133601 -0.0511395421
H 0.1953904075 0.3947437677 -0.8234782212
N 2.4044427809 -1.2706109388 -0.6268033864
C 1.0712245218 -2.3618171768 1.1627450491
H 0.4510691770 -1.8615873005 -0.8412422969
C 2.3453194599 -3.2255854511 1.0432986974
C 3.4381678846 -1.0737852293 0.4047166431
C 3.5532293601 -2.3170907582 1.3335514296
H 3.1924845269 -0.1794894636 0.9874222018
H 4.3901003119 -0.8600103381 -0.1060867489
H 3.5588150784 -2.0146557024 2.3938134565
H 4.4896184194 -2.8714512689 1.1542958924
C 2.4951999045 -3.7708946834 -0.4021454575
H 2.3152181943 -4.0560317466 1.7646293773
C 2.6940932159 -2.5251737215 -1.3362496549
H 0.1616535603 -2.9821441394 1.1746664748
H 1.0990958660 -1.8140811620 2.1209941199
H 2.0360857473 -2.5912747824 -2.2179166481
H 3.7282976426 -2.4744017916 -1.7116719674
C -4.5234743528 -1.2556234516 -0.1517785167
H -3.2747488207 -0.7335168647 1.4521892312
C -5.4699473623 -0.0744673957 -0.0752219351
H -4.9720667315 -2.1345517014 0.3404206780
H -4.3148284936 -1.5213219447 -1.1983461564
C -5.1772321610 1.1072035051 -0.7785383363
C -6.0289042510 2.2096779842 -0.6900193821
C -7.1833864048 2.1489305365 0.1007703077
C -7.4812622531 0.9784653358 0.8023853119
C -6.6259538782 -0.1259818476 0.7145064379
H -6.8614059434 -1.0409914623 1.2669972118
H -4.2766578425 1.1473036999 -1.3974897448
H -5.7939487555 3.1220516942 -1.2446693400
H -7.8491116365 3.0133852395 0.1677983273
H -8.3806044400 0.9229277958 1.4215280158
C 1.3428763950 -4.6748239223 -0.8722004478
H 3.4157138146 -4.3818965739 -0.4209355827
C 1.0967680155 -5.9003180086 0.0086448824
H 1.5716457246 -5.0049983591 -1.9012847528
H 0.4123105961 -4.0867924716 -0.9565198585
H 2.0177867693 -6.4951168280 0.1336795223
H 0.3306362084 -6.5595731347 -0.4297208662
H 0.7490557407 -5.6167896198 1.0151121602
H 1.2050924238 1.9992793781 4.3279126523
H 0.1075555812 0.2861198424 2.9375269747
C 3.2868215829 3.9264298932 0.9083931360

C 3.6069062113 3.9738327493 -0.4319821147
C 3.0763595061 2.9981502250 -1.3207150501
C 2.2217482870 2.0142105009 -0.8437338556
H 1.8775641805 1.2577004542 -1.5470316216
H 3.6917473646 4.6556127276 1.6128514143
H 4.2711510325 4.7577315680 -0.7962200955
O 3.3605885313 2.9569112737 -2.6464528902
C 4.2291279029 3.9146638621 -3.2085071922
H 4.3077133213 3.6741928796 -4.2774876854
H 5.2388212128 3.8732444712 -2.7598996544
H 3.8362550029 4.9429373384 -3.1046021401

System: Thiourea
Total energy [Ha]: -1778.76313694719
Number of imaginary frequencies: 0
Multiplicity: 3
Charge: 0

C -2.1180454136 -0.8224072910 -0.0549241355
S -1.9811109912 -1.0554775769 -1.7212477057
N -1.0634578380 -0.4764003495 0.7299657625
N -3.2908663520 -0.9788661653 0.6285558045
C 0.2849006216 -0.1714210269 0.2463412010
H -1.2323037021 -0.3421225468 1.7199955077
C 0.9457819356 0.8554006440 1.1298903015
C 1.7578286339 1.8861680787 0.5729365588
C 2.3258713505 2.8739630537 1.4815516844
N 2.1511091532 2.8384414081 2.8033552563
C 1.4019641379 1.8112451876 3.3196809217
C 0.8076678338 0.8371919685 2.5583566374
C 1.0660606647 -1.4973051331 0.0063586367
H 0.1502774416 0.2587490565 -0.7552520871
N 2.4024476179 -1.2957954612 -0.5769719234
C 1.1385793589 -2.4825229266 1.1990398920
H 0.4744846423 -1.9764259412 -0.7854359887
C 2.4388410907 -3.2996632900 1.0305555995
C 3.4366971932 -1.0883466039 0.4522186521
C 3.6186987990 -2.3585141256 1.3313572578
H 3.1503667663 -0.2318949864 1.0729029272
H 4.3727325968 -0.8105986224 -0.0572166311
H 3.6351260137 -2.0931776385 2.4011899875
H 4.5717105802 -2.8684534762 1.1124761245
C 2.5877291345 -3.7942549790 -0.4336911342
H 2.4483473992 -4.1530636047 1.7249607611
C 2.7376225472 -2.5131992466 -1.3301406294

H 0.2476456320 -3.1286170819 1.2175407674
H 1.1722370130 -1.9558428982 2.1676939075
H 2.0787296022 -2.5761311807 -2.2112894835
H 3.7683909746 -2.4121684639 -1.7054121780
C -4.5965978102 -1.2038912598 0.0236163560
H -3.3135650165 -0.6553934876 1.5913449058
C -5.4335566353 0.0577587171 -0.0531258684
H -5.1275123873 -1.9768678427 0.6032448493
H -4.4081870735 -1.6027500353 -0.9838771058
C -4.9969718887 1.1407635353 -0.8362973805
C -5.7484669307 2.3151535381 -0.8973477091
C -6.9466888498 2.4255275569 -0.1803069751
C -7.3888305137 1.3532468410 0.5980087550
C -6.6328633253 0.1768579940 0.6613780273
H -6.9817275365 -0.6602583795 1.2742106758
H -4.0668247497 1.0451529302 -1.4030726805
H -5.4013459071 3.1496164292 -1.5124844759
H -7.5336920411 3.3463272739 -0.2306693567
H -8.3228592991 1.4313038033 1.1607969615
C 1.4599581139 -4.7190587819 -0.9214913917
H 3.5272104372 -4.3739037754 -0.4832866256
C 1.2819367364 -5.9922574986 -0.0933281351
H 1.6792311486 -4.9947573835 -1.9687106298
H 0.5061048247 -4.1652853636 -0.9611790792
H 2.2263984002 -6.5584461398 -0.0225717044
H 0.5268394870 -6.6572339720 -0.5420923804
H 0.9535226548 -5.7695179658 0.9346575508
H 1.3001311331 1.8023794929 4.4102628588
H 0.2424078750 0.0477759590 3.0603286910
C 3.1210595895 3.9322853820 0.9255122748
C 3.4037817222 4.0395130516 -0.4609078674
C 2.9002802536 3.0757933325 -1.3086534673
C 2.0769848187 1.9852507270 -0.7921606428
H 1.7504571834 1.2374044967 -1.5129598037
H 3.5120226591 4.6677486252 1.6306053655
H 4.0123782275 4.8674441477 -0.8229139689
O 3.0797142735 3.0104298500 -2.6374553480
C 3.8483969508 4.0093427649 -3.2806488776
H 3.8608791202 3.7563102045 -4.3485300599
H 4.8829048211 4.0272709524 -2.8944752401
H 3.3984658000 5.0086551065 -3.1444874998

System: Thiosquaramide
Total energy [Ha]: -2291.12163111085
Number of imaginary frequencies: 0

Multiplicity: 1
Charge: 0

C 1.4745976037 -0.3962904362 0.2591461616
C 2.8574205437 -0.6848387106 0.1074824794
C 2.9626707366 -1.1126700535 1.5040043900
C 1.5004415930 -0.7990145649 1.6669243928
S 0.4203913855 -0.8776058555 2.8994285078
S 4.1457298698 -1.6814580321 2.4882312136
N 0.5317224144 0.0760207480 -0.5640812799
N 3.7036399290 -0.6158525083 -0.9287734030
C -0.8851299272 0.1765241115 -0.1875365494
H 0.7710164580 0.1900969348 -1.5418134692
C -1.5704744796 1.2976628391 -0.9408619867
C -2.5656974961 2.1008462740 -0.2839387303
C -3.1737219952 3.1566117323 -1.0511072072
N -2.8593012223 3.4088841722 -2.3524306063
C -1.9583875384 2.6482551268 -2.9257316566
C -1.2886140889 1.5861536722 -2.2623058171
C -1.5365728808 -1.2348477453 -0.2782771242
H -0.8885786407 0.4217345533 0.8805169684
N -2.9340155102 -1.2761489009 0.1734219552
C -1.3712794493 -1.9814325985 -1.6264107281
H -0.9793744873 -1.7959660769 0.4854940479
C -2.5887607586 -2.9216809568 -1.7702803540
C -3.8888774548 -0.9731125780 -0.9058733216
C -3.8227888667 -2.0412337948 -2.0365158334
H -3.6747918121 0.0252929805 -1.3023385710
H -4.8933411155 -0.9191177826 -0.4583100305
H -3.7417816420 -1.5601679211 -3.0253563656
H -4.7320548322 -2.6645544385 -2.0577656725
C -2.8335847308 -3.7049432067 -0.4524202616
H -2.4351838625 -3.6193416056 -2.6069355246
C -3.1918679137 -2.6431478198 0.6481070399
H -0.4221442609 -2.5395965027 -1.6444736278
H -1.3531054583 -1.2866397738 -2.4844382840
H -2.6010456043 -2.8136067723 1.5625672776
H -4.2533015938 -2.7115641643 0.9335327941
C 5.1602479606 -0.7701733886 -0.8127765503
H 3.3790037174 -0.1279492074 -1.7584624783
C 5.8639220365 0.5646590794 -0.6814218703
H 5.5184562879 -1.3088639070 -1.7038709533
H 5.3461313798 -1.3987151445 0.0700498765
C 5.8221676251 1.2600060057 0.5382978194
C 6.4234103053 2.5148676730 0.6535918688

C 7.0727797131 3.0896081571 -0.4455831337
C 7.1230335509 2.4014936213 -1.6608281435
C 6.5193291351 1.1446518409 -1.7762610277
H 6.5641100320 0.6050093504 -2.7277465090
H 5.3257131905 0.8019282286 1.3985665749
H 6.3901006196 3.0460137738 1.6083165663
H 7.5436141839 4.0716467768 -0.3520247285
H 7.6341658399 2.8421573390 -2.5208325145
C -1.6758639569 -4.6200956068 -0.0204755816
H -3.7125999782 -4.3515778436 -0.6256491340
C -1.2950417559 -5.6923713733 -1.0415699738
H -1.9672434117 -5.1065164817 0.9272324679
H -0.7886129354 -4.0124171731 0.2290329461
H -2.1687986583 -6.3070181665 -1.3175469311
H -0.5239449836 -6.3693747131 -0.6407915797
H -0.8955363638 -5.2524105783 -1.9697266067
H -1.7207516982 2.8588983044 -3.9763340002
H -0.5547963655 1.0106470763 -2.8329470374
C -4.1595970487 3.9668869312 -0.4303127577
C -4.5515770636 3.7501096093 0.8738226994
C -3.9645750786 2.6928968365 1.6231404817
C -2.9905408135 1.8882295657 1.0483268041
H -2.6043955307 1.0626637450 1.6437240371
H -4.6016650207 4.7648401039 -1.0298611979
H -5.3157570813 4.3882716356 1.3177492447
O -4.3068432778 2.3986501557 2.9007880072
C -5.3059596473 3.1493316572 3.5538917616
H -5.4097070455 2.7202261496 4.5596186257
H -6.2801793598 3.0812763032 3.0354229155
H -5.0278755469 4.2150005199 3.6501195911

System: Thiosquaramide
Total energy [Ha]: -2291.05185055501
Number of imaginary frequencies: 0
Multiplicity: 3
Charge: 0

C -1.4450499905 0.5319012471 0.2555845414
C -2.9374318101 0.7472351957 0.0945201398
C -3.0344271945 1.2396301521 1.4141714683
C -1.4842981698 1.0166342267 1.5770854950
S -0.3950347432 1.2076502558 2.8322882255
S -4.2429837292 1.8081792384 2.4101665730
N -0.5300127333 0.0059057514 -0.5696082371
N -3.7532220495 0.5506302903 -0.9536619494

C 0.8961462171 -0.0774280887 -0.2375061754
H -0.8457637836 -0.4378440873 -1.4237661272
C 1.5728653376 -1.1382892115 -1.0785081207
C 2.5808041042 -1.9864986495 -0.5034107534
C 3.1459868743 -3.0058677764 -1.3479627107
N 2.7927162087 -3.1739449486 -2.6528614454
C 1.8952603910 -2.3613975837 -3.1561028494
C 1.2581255281 -1.3339073037 -2.4106867908
C 1.5164120627 1.3522196585 -0.2732546389
H 0.9487027502 -0.3723828475 0.8206475434
N 2.9180400880 1.3879887318 0.1652661607
C 1.3214553786 2.1627035705 -1.5770289985
H 0.9632279088 1.8662017165 0.5236883429
C 2.5207176186 3.1305739741 -1.6811535294
C 3.8647247655 1.1617206565 -0.9399392124
C 3.7659753895 2.2893828419 -2.0094846751
H 3.6632889317 0.1816856097 -1.3867115748
H 4.8756935075 1.1028321568 -0.5075690350
H 3.6823688890 1.8616464872 -3.0223961582
H 4.6643506127 2.9285484782 -2.0053440086
C 2.7708486394 3.8446986939 -0.3254228969
H 2.3454916975 3.8708351605 -2.4759127637
C 3.1561114820 2.7316342267 0.7142251044
H 0.3618036867 2.7015182573 -1.5517170855
H 1.3029763830 1.5174101500 -2.4721007598
H 2.5662493091 2.8400158361 1.6382830527
H 4.2181678509 2.8032021176 0.9968747164
C -5.2087749796 0.7246336433 -0.8933808272
H -3.4083124108 -0.0296608304 -1.7132388315
C -5.9228864924 -0.5968644726 -0.7000639932
H -5.5371551413 1.2081533719 -1.8271323354
H -5.4124500455 1.4084191442 -0.0569647449
C -5.8989615514 -1.2268062943 0.5560833598
C -6.5089683966 -2.4704548769 0.7298511384
C -7.1503735120 -3.0986346269 -0.3445984870
C -7.1828097318 -2.4757359761 -1.5950828843
C -6.5691935733 -1.2306562118 -1.7701996044
H -6.5992578629 -0.7427875072 -2.7496321798
H -5.4094382266 -0.7270359884 1.3963763373
H -6.4895187417 -2.9508265912 1.7115208725
H -7.6292431060 -4.0711473785 -0.2040892689
H -7.6876253875 -2.9578663150 -2.4363448425
C 1.6046485290 4.7192285115 0.1644830443
H 3.6393615467 4.5116318828 -0.4736261081
C 1.2046109757 5.8412631353 -0.7935118887

H 1.8957417668 5.1553566950 1.1364864811
H 0.7266130191 4.0871224938 0.3836590587
H 2.0695979749 6.4793502265 -1.0428213449
H 0.4301957621 6.4864392329 -0.3492639506
H 0.8007815297 5.4485039450 -1.7406101403
H 1.6313630128 -2.4996041595 -4.2123938727
H 0.5235358705 -0.7054175058 -2.9217826944
C 4.1313918750 -3.8699825064 -0.8031906518
C 4.5663011003 -3.7349568225 0.4984137164
C 4.0281748276 -2.7066817328 1.3217018643
C 3.0548564759 -1.8522084004 0.8223287577
H 2.7108751356 -1.0448446388 1.4671025605
H 4.5390849228 -4.6410144154 -1.4596518956
H 5.3281744699 -4.4128507324 0.8834650296
O 4.4165322180 -2.4891997758 2.6010017340
C 5.4112880070 -3.3014993948 3.1843075430
H 5.5498455062 -2.9345031334 4.2101733956
H 6.3743851914 -3.2266724453 2.6465650836
H 5.1073092325 -4.3635463049 3.2252894301

6.7.3. Structures of complexes with one ligand

System: Acetate-Ni-L
Ligand: Squaramide
Configuration: O
Total energy [Ha]: -3381.64140505247
Number of imaginary frequencies: 2 (-17.73 and -7.43 cm-1)
Multiplicity: 1
Charge: +1

Ni	3.199768	1.964280	0.499026
C	4.143783	3.994162	0.649005
O	3.016995	3.787549	0.080059
O	4.692556	2.933714	1.101875
C	4.735102	5.347436	0.804399
H	4.377192	6.010375	0.005755
H	5.831428	5.282661	0.811302
H	4.407056	5.758037	1.774112
C	0.670594	-1.094003	0.505011
C	1.866426	-1.743391	1.050886
C	2.590571	-0.484785	0.925423
C	1.474268	0.100435	0.245665
O	1.479688	1.270397	-0.198368
O	3.655203	0.130217	1.166075
N	-0.572756	-1.499389	0.365609

N	2.138064	-2.936625	1.539134
C	-1.767727	-0.728628	-0.083961
H	-0.782682	-2.456149	0.641372
C	-2.993380	-1.493969	0.385963
C	-4.138184	-1.641975	-0.467491
C	-5.220014	-2.451483	0.033951
N	-5.209905	-3.031945	1.263271
C	-4.164771	-2.846313	2.032882
C	-3.029901	-2.091741	1.634670
C	-1.650233	0.771454	0.324539
H	-1.757653	-0.739761	-1.182570
N	-2.773882	1.570728	-0.177574
C	-1.379235	1.072312	1.824106
H	-0.788364	1.139017	-0.247006
C	-2.084882	2.406108	2.156207
C	-3.944183	1.547727	0.723660
C	-3.600715	2.149827	2.117093
H	-4.306085	0.519448	0.819485
H	-4.748434	2.109542	0.226156
H	-3.892517	1.459085	2.924877
H	-4.142758	3.092696	2.290228
C	-1.755292	3.489163	1.096097
H	-1.785716	2.748150	3.157778
C	-2.302483	2.964324	-0.278600
H	-0.298090	1.142916	2.023018
H	-1.778145	0.284824	2.483516
H	-1.518544	3.014798	-1.051895
H	-3.142743	3.579009	-0.633518
C	3.510241	-3.327325	1.953567
H	1.456794	-3.682520	1.421663
C	4.352029	-3.777789	0.782426
H	3.399341	-4.121427	2.705043
H	3.955452	-2.453234	2.450222
C	5.101214	-2.839039	0.055630
C	5.834200	-3.242709	-1.063485
C	5.823231	-4.582990	-1.463790
C	5.082963	-5.523087	-0.739496
C	4.350126	-5.121543	0.380412
H	3.782663	-5.862838	0.951838
H	5.120597	-1.792498	0.374060
H	6.424780	-2.510408	-1.619197
H	6.400895	-4.897551	-2.336160
H	5.083230	-6.572768	-1.042540
C	-0.275794	3.897029	1.014328
H	-2.325381	4.389985	1.383042

C	0.315759	4.449880	2.311111
H	-0.178432	4.658149	0.220640
H	0.336213	3.050707	0.668366
H	-0.274173	5.297999	2.696091
H	1.345223	4.804660	2.144541
H	0.353897	3.688153	3.107621
H	-4.183840	-3.308674	3.027333
H	-2.196484	-1.992270	2.334860
C	-6.363115	-2.640828	-0.787249
C	-6.461590	-2.050150	-2.027515
C	-5.406998	-1.221269	-2.507853
C	-4.269878	-1.024969	-1.732420
H	-3.520522	-0.329352	-2.108365
H	-7.166449	-3.265234	-0.392346
H	-7.356065	-2.212743	-2.628711
O	-5.437901	-0.586922	-3.696729
C	-6.566914	-0.711711	-4.543757
H	-6.354054	-0.102549	-5.431692
H	-7.484347	-0.331687	-4.060836
H	-6.728857	-1.757816	-4.858052

System: Ac-Ni-L
Ligand: Squaramide
Configuration: O
Total energy [Ha]: -3381.64477811914
Number of imaginary frequencies: 1 (-32.54 cm-1)
Multiplicity: 3
Charge: +1

Ni	3.419632	1.806688	-0.526561
C	4.551766	3.801931	-0.878287
O	3.278226	3.726468	-0.957097
O	5.164685	2.715970	-0.605192
C	5.274447	5.093533	-1.061257
H	4.696180	5.765240	-1.708502
H	6.279788	4.913744	-1.464068
H	5.381795	5.570902	-0.072929
C	0.683618	-1.047038	0.361961
C	1.893845	-1.697764	0.862990
C	2.684503	-0.551912	0.424792
C	1.516060	0.015441	-0.204485
O	1.492619	1.083060	-0.851852
O	3.824806	-0.043075	0.439355
N	-0.592938	-1.357963	0.436800
N	2.150502	-2.806177	1.527354

C	-1.767522	-0.544829	0.013390
H	-0.836640	-2.241544	0.878299
C	-3.002342	-1.167439	0.640269
C	-4.224949	-1.268657	-0.105589
C	-5.324240	-1.943859	0.536274
N	-5.258406	-2.439420	1.800552
C	-4.140755	-2.293869	2.470445
C	-2.984622	-1.669921	1.930529
C	-1.507947	0.970302	0.287996
H	-1.850937	-0.642510	-1.077890
N	-2.617016	1.818775	-0.159136
C	-1.062159	1.349449	1.724072
H	-0.689476	1.229540	-0.393840
C	-1.598554	2.772283	1.999269
C	-3.673232	1.967202	0.861005
C	-3.127778	2.668538	2.138806
H	-4.083097	0.981242	1.101279
H	-4.494699	2.538018	0.403171
H	-3.390133	2.096056	3.043309
H	-3.560494	3.674077	2.262331
C	-1.285030	3.719994	0.812231
H	-1.162780	3.165991	2.929012
C	-2.057252	3.155980	-0.433947
H	0.035938	1.313350	1.817021
H	-1.471720	0.659702	2.479406
H	-1.389845	3.088991	-1.308633
H	-2.890815	3.815053	-0.717669
C	3.535133	-3.239956	1.842531
H	1.410084	-3.493262	1.644595
C	4.161752	-4.011712	0.704877
H	3.476727	-3.843284	2.759396
H	4.110846	-2.332046	2.071909
C	4.866464	-3.330299	-0.300069
C	5.397245	-4.033296	-1.384652
C	5.227443	-5.419130	-1.472987
C	4.532230	-6.103577	-0.470729
C	4.002063	-5.402185	0.615259
H	3.471829	-5.944351	1.404497
H	5.010148	-2.248217	-0.224351
H	5.953691	-3.499456	-2.158757
H	5.646818	-5.968651	-2.319075
H	4.410256	-7.187666	-0.530533
C	0.208917	3.929903	0.515485
H	-1.707994	4.704597	1.076668
C	1.054356	4.423895	1.688559

H	0.291196	4.650740	-0.316151
H	0.644148	3.000134	0.118425
H	0.647502	5.353344	2.120305
H	2.083293	4.632018	1.354969
H	1.109876	3.682458	2.503243
H	-4.114169	-2.683517	3.495477
H	-2.094531	-1.593839	2.561085
C	-6.543616	-2.086896	-0.177405
C	-6.696815	-1.574510	-1.446674
C	-5.622056	-0.874022	-2.065947
C	-4.411433	-0.728439	-1.398064
H	-3.641269	-0.130184	-1.882758
H	-7.359258	-2.610471	0.324479
H	-7.648617	-1.699460	-1.962452
O	-5.699889	-0.315716	-3.290606
C	-6.900063	-0.402776	-4.038608
H	-6.714436	0.128062	-4.981251
H	-7.744024	0.080878	-3.515781
H	-7.166399	-1.450398	-4.264331

System: Acetate-Ni-L
Ligand: Squaramide
Configuration: N
Total energy [Ha]: -3381.64544123799
Number of imaginary frequencies: 1 (-6.28 cm-1)
Multiplicity: 1
Charge: +1

Ni	0.7704815344	-0.6605686806	1.1567134593
C	2.8041086362	-1.6756729290	1.2310448091
O	1.7109123715	-2.2895806235	1.4553943642
O	2.6657291359	-0.4245033911	0.9809045618
C	4.1367275168	-2.3288731627	1.2656360425
H	4.0342051526	-3.4153052312	1.1503365834
H	4.6062769700	-2.1145418124	2.2399616311
H	4.7792875475	-1.8931522601	0.4872072002
C	0.7302937466	1.7663821015	-0.3441944185
C	2.0106247318	2.3225486741	-0.3018258785
C	1.8588212386	2.8304220991	-1.7374637974
C	0.4413901948	2.1721026277	-1.7340018548
O	-0.4696911418	2.0238453910	-2.5071890616
O	2.5726793126	3.4260506989	-2.4877067022
N	-0.0216801771	1.0161554197	0.5978334442
N	2.9992574799	2.4019394449	0.5670626142
C	-1.2567328052	0.3332218338	0.0442469953

H	-0.2653599468	1.5985142615	1.4090926666
C	-1.2921736078	-0.9788777509	0.8142456028
C	-1.5099055187	-2.2384823394	0.1430732077
C	-1.4355130075	-3.4261115618	0.9554237173
N	-1.2401622715	-3.3977678547	2.3022034230
C	-1.0998399708	-2.2488954209	2.9080656760
C	-1.0972112317	-0.9984229714	2.2132599211
C	-2.4881629267	1.2547654272	-0.0094777655
H	-1.0148922991	0.1041089303	-0.9976830086
N	-3.6515257680	0.5361858313	-0.5421489272
C	-2.8483798133	2.0383170722	1.2794896878
H	-2.2148608345	1.9826443866	-0.7859403165
C	-4.3864161249	2.1971804470	1.2823454949
C	-4.3891231741	-0.1798693040	0.5158185252
C	-4.9895650150	0.8085582969	1.5575261836
H	-3.7147729763	-0.8961647661	1.0015979204
H	-5.1703788717	-0.7811756121	0.0280321344
H	-4.7594501190	0.4854892069	2.5860810220
H	-6.0869880258	0.8544421377	1.4781469377
C	-4.8903059859	2.6749732810	-0.1048518452
H	-4.6896926345	2.9063679427	2.0653689471
C	-4.5520573903	1.5431950577	-1.1357832431
H	-2.3435900507	3.0178576677	1.2998931208
H	-2.5528632686	1.5007042653	2.1981680754
H	-4.0672443658	1.9598790352	-2.0329672821
H	-5.4612749669	1.0246713381	-1.4741383730
C	4.3503757257	2.8798824987	0.2095899426
H	2.9572967525	1.7683559804	1.3634581164
C	5.2637161810	1.7220983797	-0.1326069318
H	4.7414934705	3.4506957009	1.0642966066
H	4.2419581382	3.5666282345	-0.6402444240
C	5.1799745015	1.1155177476	-1.3959629878
C	5.9765199631	0.0102036750	-1.7021446876
C	6.8670666786	-0.4990472658	-0.7483634420
C	6.9577996020	0.1022047055	0.5108768564
C	6.1557199641	1.2065857925	0.8173701751
H	6.2358765780	1.6796574528	1.8007137865
H	4.5007893036	1.5264115480	-2.1481872075
H	5.9160033411	-0.4463625720	-2.6931326000
H	7.5002632608	-1.3555520844	-0.9931310243
H	7.6616516489	-0.2827555223	1.2530799168
C	-4.3603546944	4.0479628748	-0.5517704482
H	-5.9872046874	2.7642827282	-0.0225502016
C	-4.6753862173	5.1956504318	0.4074190094
H	-4.7994251882	4.2673793507	-1.5404435680

H	-3.2704516539	3.9955451738	-0.7283375600
H	-5.7559430060	5.2500546396	0.6207468112
H	-4.3720106050	6.1643303956	-0.0189919146
H	-4.1537250972	5.0853664329	1.3719924034
H	-0.9709511958	-2.2567681330	3.9961171086
H	-1.0990416471	-0.0804648262	2.8079334722
C	-1.6109663860	-4.6842821049	0.3286011001
C	-1.8703330184	-4.7790605683	-1.0232069992
C	-1.9801057260	-3.6013656213	-1.8153880159
C	-1.7977693523	-2.3469037950	-1.2274381901
H	-1.9430834465	-1.4684852067	-1.8556677954
H	-1.5432344491	-5.5743973403	0.9562596793
H	-2.0038383510	-5.7618130464	-1.4749320857
O	-2.2586687540	-3.6035751140	-3.1246066112
C	-2.4880776829	-4.8247689635	-3.8150324773
H	-2.7032512821	-4.5508843619	-4.8551962184
H	-3.3532126666	-5.3668993842	-3.3966829049
H	-1.5977349959	-5.4760672138	-3.7894293196

System: Acetate-Ni-L
Ligand: Squaramide
Configuration: N
Total energy [Ha]: -3381.65076646203
Number of imaginary frequencies: 0
Multiplicity: 3
Charge: +1

Ni	0.263263	-0.729694	3.083049
C	2.545948	-0.380485	3.539247
O	2.148435	-0.780408	2.382774
O	1.644791	-0.218297	4.418211
C	3.982699	-0.081926	3.801468
H	4.614431	-0.872822	3.372790
H	4.165652	0.032143	4.876665
H	4.233153	0.860626	3.286564
C	0.122510	1.974976	1.703669
C	1.358344	2.256987	1.130362
C	0.976582	3.731464	0.964046
C	-0.410859	3.344531	1.594888
O	-1.484806	3.846450	1.796177
O	1.501078	4.671415	0.446466
N	-0.569600	0.771684	1.958088
N	2.429417	1.566338	0.764003
C	-0.758261	-0.068977	0.705139
H	-1.488788	1.034544	2.335503

C	-0.928150	-1.509230	1.152723
C	-0.267998	-2.572538	0.448927
C	-0.301203	-3.879262	1.059131
N	-0.957817	-4.144272	2.219954
C	-1.604136	-3.176707	2.821705
C	-1.612659	-1.829339	2.339105
C	-1.790062	0.599117	-0.229311
H	0.203450	-0.016610	0.182922
N	-1.902984	-0.092626	-1.513546
C	-3.182324	0.901872	0.382756
H	-1.314134	1.563288	-0.463478
C	-4.193712	0.831554	-0.785328
C	-2.889202	-1.188775	-1.456421
C	-4.323365	-0.644542	-1.201565
H	-2.594454	-1.893564	-0.667922
H	-2.824812	-1.746648	-2.402591
H	-4.830781	-1.225605	-0.414847
H	-4.945056	-0.723211	-2.106854
C	-3.663456	1.630983	-2.005291
H	-5.168327	1.223278	-0.462131
C	-2.364224	0.904231	-2.498991
H	-3.185349	1.890777	0.867361
H	-3.472146	0.158128	1.147325
H	-1.549633	1.624962	-2.668239
H	-2.529286	0.381920	-3.453203
C	3.265119	2.007859	-0.372974
H	2.437603	0.569103	0.994207
C	2.541663	1.723420	-1.674960
H	4.218913	1.465100	-0.309784
H	3.468912	3.080903	-0.258163
C	1.869064	2.749027	-2.355079
C	1.112166	2.459944	-3.496100
C	1.008618	1.144249	-3.956614
C	1.668301	0.114273	-3.277176
C	2.434091	0.404498	-2.145382
H	2.946781	-0.409496	-1.622264
H	1.934357	3.776311	-1.986934
H	0.600427	3.268062	-4.024626
H	0.412533	0.919754	-4.844468
H	1.585252	-0.919542	-3.617388
C	-3.435130	3.129129	-1.740498
H	-4.432541	1.554115	-2.793048
C	-4.682169	3.892827	-1.295871
H	-3.042783	3.576384	-2.671074
H	-2.635555	3.270349	-0.991364

H	-5.507216	3.764755	-2.016328
H	-4.475987	4.970596	-1.210793
H	-5.044008	3.554393	-0.311686
H	-2.153243	-3.423491	3.737747
H	-2.265807	-1.102066	2.835933
C	0.382367	-4.942104	0.414313
C	1.057661	-4.740534	-0.770030
C	1.072779	-3.451074	-1.378635
C	0.407457	-2.387189	-0.774047
H	0.377124	-1.431230	-1.297237
H	0.349147	-5.924935	0.887531
H	1.571231	-5.579525	-1.239104
O	1.709438	-3.181654	-2.531405
C	2.420008	-4.201330	-3.224284
H	2.848432	-3.722573	-4.113525
H	1.748491	-5.017090	-3.541006
H	3.236942	-4.613148	-2.607970

System: Acetate-Ni-L
Ligand: Squaramide
Configuration: Lin
Total energy [Ha]: -3381.65875834470
Number of imaginary frequencies: 1 (-42.84 cm-1)
Multiplicity: 1
Charge: +1

Ni	2.071419	-2.739301	0.968350
C	2.760382	-4.769451	1.645852
O	1.767613	-4.173008	2.176026
O	3.323304	-4.118265	0.697007
C	3.244438	-6.101523	2.098233
H	2.506559	-6.568635	2.762307
H	4.195093	-5.968370	2.639803
H	3.447717	-6.737768	1.224585
C	-1.432130	-0.376414	0.827698
C	-2.547643	-1.260137	0.936140
C	-1.656727	-2.407619	1.306128
C	-0.514453	-1.423539	1.278347
O	0.710537	-1.471142	1.499773
O	-1.811061	-3.589616	1.486452
N	-1.353925	0.899802	0.400048
N	-3.848372	-1.157974	0.746008
C	-0.144348	1.742595	0.306176
H	-2.230029	1.369976	0.202199
C	1.012581	0.912576	-0.226378

C	2.270335	0.925417	0.464940
C	3.044467	-0.277679	0.398793
N	2.616084	-1.356242	-0.331010
C	1.629835	-1.196211	-1.206524
C	0.810068	-0.053143	-1.201220
C	-0.522897	3.057012	-0.424443
H	0.137920	2.050357	1.323746
N	0.562286	4.042707	-0.365820
C	-1.078798	2.926895	-1.868915
H	-1.322981	3.477954	0.208223
C	-0.630505	4.189318	-2.638843
C	1.541620	3.852522	-1.454859
C	0.890245	4.087319	-2.847340
H	1.959639	2.841106	-1.386197
H	2.378407	4.543353	-1.273513
H	1.127952	3.260491	-3.536083
H	1.267638	5.010391	-3.314829
C	-0.920781	5.468140	-1.812984
H	-1.141520	4.239384	-3.610877
C	-0.040979	5.380193	-0.515853
H	-2.176226	2.827870	-1.864731
H	-0.681071	2.037454	-2.382879
H	-0.641412	5.599165	0.382390
H	0.776418	6.116005	-0.537342
C	-4.763940	-2.320861	0.791564
H	-4.232650	-0.289400	0.382378
C	-5.004664	-2.902247	-0.583545
H	-5.702665	-1.979489	1.251585
H	-4.303435	-3.063178	1.456655
C	-4.107629	-3.847644	-1.106014
C	-4.296320	-4.356582	-2.393057
C	-5.378193	-3.924311	-3.168482
C	-6.276487	-2.985697	-2.651607
C	-6.090110	-2.477182	-1.362599
H	-6.804121	-1.754077	-0.955530
H	-3.272732	-4.193612	-0.489908
H	-3.603149	-5.102804	-2.789421
H	-5.527861	-4.328263	-4.172663
H	-7.129374	-2.655657	-3.249573
C	-2.406314	5.711481	-1.501174
H	-0.573446	6.322539	-2.418979
C	-3.312616	5.798005	-2.729993
H	-2.480575	6.650798	-0.925677
H	-2.782702	4.926063	-0.819812
H	-2.949559	6.559468	-3.440116

H	-4.340879	6.071955	-2.446908
H	-3.364691	4.840382	-3.272473
H	1.365769	-2.065997	-1.815604
H	-0.104063	-0.066257	-1.795720
C	4.206897	-0.398821	1.194671
C	4.635683	0.654245	1.977154
C	3.893598	1.870144	2.012823
C	2.715186	1.986934	1.277297
H	2.169164	2.931904	1.307862
H	4.765904	-1.335906	1.150608
H	5.549605	0.546372	2.560544
O	4.254749	2.944088	2.732201
C	5.441942	2.925949	3.510007
H	5.511233	3.912469	3.985138
H	6.335994	2.765657	2.882692
H	5.401372	2.151468	4.295639

System: Acetate-Ni-L
Ligand: Squaramide
Configuration: Lin
Total energy [Ha]: -3381.67794043894
Number of imaginary frequencies: 0
Multiplicity: 3
Charge: +1

Ni	-2.265546	-2.375047	-0.158068
C	-1.452507	-4.541931	-0.529328
O	-0.861472	-3.740842	0.278027
O	-2.523277	-4.119495	-1.069480
C	-0.880130	-5.880714	-0.849978
H	-0.342400	-6.287272	0.016759
H	-0.158912	-5.725808	-1.668184
H	-1.666549	-6.565642	-1.191120
C	1.046545	-0.343673	-1.542068
C	1.950592	-1.423967	-1.764394
C	0.828563	-2.362153	-2.073035
C	-0.098277	-1.245205	-1.677667
O	-1.318459	-1.158288	-1.435703
O	0.740033	-3.523444	-2.389840
N	1.252530	0.927145	-1.154630
N	3.254401	-1.604858	-1.646846
C	0.216915	1.872636	-0.695469
H	2.206433	1.270095	-1.170052
C	-0.705528	1.170172	0.291496
C	-2.129717	1.299706	0.156319

C	-2.923683	0.209908	0.643971
N	-2.352454	-0.879201	1.261126
C	-1.070208	-0.809650	1.615446
C	-0.214623	0.211960	1.163217
C	0.922156	3.183819	-0.258842
H	-0.403891	2.147009	-1.560787
N	-0.034270	4.241483	0.083476
C	2.016652	3.048665	0.835683
H	1.412683	3.529682	-1.183302
C	2.009124	4.368362	1.640457
C	-0.472939	4.158283	1.491132
C	0.712747	4.397609	2.468489
H	-0.926930	3.175636	1.665257
H	-1.275072	4.897463	1.633355
H	0.736863	3.623622	3.252953
H	0.618606	5.367916	2.980998
C	1.998669	5.590013	0.685246
H	2.884041	4.410184	2.304404
C	0.648378	5.534304	-0.111875
H	3.007759	2.864650	0.390290
H	1.810327	2.210602	1.520901
H	0.823943	5.682041	-1.189929
H	-0.039786	6.330116	0.209346
C	3.825335	-2.965290	-1.487972
H	3.816480	-0.843682	-1.271842
C	3.793493	-3.383965	-0.032138
H	4.850522	-2.947426	-1.883032
H	3.222673	-3.638845	-2.112078
C	2.580692	-3.790332	0.551984
C	2.521208	-4.092353	1.913852
C	3.672198	-4.001746	2.705759
C	4.883634	-3.609903	2.128766
C	4.942659	-3.298572	0.765815
H	5.894606	-2.994317	0.319956
H	1.674432	-3.882327	-0.049516
H	1.569747	-4.408576	2.348772
H	3.627278	-4.244938	3.770189
H	5.787452	-3.548502	2.739806
C	3.220498	5.698447	-0.241406
H	1.996038	6.491412	1.321979
C	4.568548	5.764487	0.476640
H	3.092450	6.605215	-0.858138
H	3.225993	4.859739	-0.962791
H	4.588040	6.583762	1.214315
H	5.388635	5.941480	-0.236482

H	4.798708	4.830875	1.015219
H	-0.658904	-1.680636	2.134676
H	0.856893	0.099349	1.332220
C	-4.315520	0.206711	0.394144
C	-4.919210	1.261632	-0.261199
C	-4.140907	2.367411	-0.707019
C	-2.760350	2.368807	-0.508133
H	-2.184524	3.235878	-0.836969
H	-4.907298	-0.635670	0.760021
H	-5.996770	1.242255	-0.420869
O	-4.655582	3.439757	-1.326492
C	-6.050849	3.529210	-1.576179
H	-6.209356	4.487753	-2.085715
H	-6.631140	3.520250	-0.637400
H	-6.398830	2.711959	-2.231394

System: Acetate-Ni-L
Ligand: Squaramide
Configuration: Din
Total energy [Ha]: -3381.70117793521
Number of imaginary frequencies: 0
Multiplicity: 1
Charge: +1

Ni	0.966295	-0.854903	-1.661342
C	1.831015	0.884619	-2.842877
O	2.492333	0.232763	-1.955254
O	0.656274	0.452336	-3.047502
C	2.407252	2.082479	-3.508621
H	3.389341	1.828963	-3.934868
H	2.572554	2.841744	-2.726654
H	1.731112	2.460974	-4.284460
C	-2.115244	-0.761154	0.076391
C	-3.506250	-0.816189	-0.227210
C	-3.234555	-1.576143	-1.497541
C	-1.768557	-1.401374	-1.177752
O	-0.711260	-1.713009	-1.768600
O	-3.911416	-2.060482	-2.365102
N	-1.398581	-0.286247	1.111653
N	-4.617327	-0.399101	0.345743
C	0.074427	-0.278040	1.127191
H	-1.861448	0.286984	1.807969
C	0.540484	0.553953	2.305226
C	1.445538	1.653563	2.134085
C	1.786056	2.399979	3.319379

N	1.307479	2.098123	4.553680
C	0.482603	1.084262	4.674821
C	0.065901	0.288217	3.577741
C	0.621484	-1.753451	1.016319
H	0.401792	0.238268	0.213274
N	1.575608	-1.902249	-0.144627
C	1.249225	-2.400098	2.271815
H	-0.241028	-2.360678	0.724461
C	2.367266	-3.358564	1.825853
C	2.960648	-1.496127	0.266113
C	3.525482	-2.492726	1.304386
H	2.897047	-0.474410	0.658258
H	3.570846	-1.442721	-0.642221
H	4.000213	-1.942047	2.130496
H	4.302153	-3.128713	0.852586
C	1.886219	-4.268174	0.673481
H	2.703962	-3.964745	2.678053
C	1.604505	-3.347256	-0.548832
H	0.468146	-2.916396	2.848766
H	1.683316	-1.642362	2.937998
H	0.639781	-3.583504	-1.016485
H	2.378284	-3.435456	-1.324403
C	-5.942040	-0.488529	-0.315871
H	-4.556075	0.208269	1.159348
C	-6.244352	0.747697	-1.133038
H	-6.688931	-0.637929	0.476861
H	-5.924251	-1.386258	-0.947491
C	-5.807687	0.830794	-2.464784
C	-6.034697	1.993429	-3.205450
C	-6.696355	3.080283	-2.623521
C	-7.138174	3.001035	-1.299201
C	-6.913509	1.837712	-0.557513
H	-7.273453	1.774411	0.474405
H	-5.301373	-0.023530	-2.922534
H	-5.701975	2.047908	-4.244942
H	-6.876565	3.986924	-3.206415
H	-7.666373	3.843151	-0.845441
C	0.682534	-5.164545	1.010468
H	2.726757	-4.933080	0.413431
C	0.911240	-6.111228	2.188201
H	0.440754	-5.755321	0.109849
H	-0.212221	-4.543482	1.199573
H	1.811723	-6.728204	2.033130
H	0.058499	-6.795573	2.314335
H	1.039083	-5.568594	3.138276

H	0.110091	0.857318	5.680865
H	-0.619703	-0.544603	3.755438
C	2.656605	3.516632	3.194005
C	3.169937	3.897928	1.974574
C	2.837504	3.159308	0.803171
C	1.998667	2.056154	0.892916
H	1.806957	1.510075	-0.028591
H	2.897067	4.067155	4.105303
H	3.825984	4.766163	1.918411
O	3.292473	3.471957	-0.432232
C	4.188527	4.559403	-0.606358
H	4.425585	4.593151	-1.677604
H	5.122346	4.411820	-0.037423
H	3.727896	5.517900	-0.311412

System: Acetate-Ni-L
Ligand: Squaramide
Configuration: Din
Total energy [Ha]: -3381.70993099345
Number of imaginary frequencies: 0
Multiplicity: 3
Charge: +1

Ni	-0.740226	0.959768	-2.245885
C	-1.843548	-0.957502	-3.072674
O	-2.202245	0.214445	-3.412536
O	-0.857113	-1.050048	-2.262447
C	-2.548039	-2.180690	-3.557091
H	-3.328216	-1.920793	-4.282105
H	-2.975973	-2.697132	-2.683212
H	-1.814418	-2.866453	-4.007194
C	2.035212	0.903785	0.207148
C	3.456383	1.039159	0.204722
C	3.427263	1.705793	-1.142739
C	1.936398	1.479657	-1.118141
O	1.009468	1.709506	-1.927297
O	4.252873	2.166397	-1.887042
N	1.168331	0.410504	1.106800
N	4.431724	0.729301	1.032908
C	-0.284712	0.240460	0.895277
H	1.549822	-0.095502	1.898156
C	-0.820669	-0.556304	2.076080
C	-1.656525	-1.709052	1.908381
C	-2.144394	-2.333258	3.114730
N	-1.824467	-1.902769	4.361879

C	-1.028969	-0.865615	4.479257
C	-0.509211	-0.159719	3.366020
C	-0.970364	1.645513	0.645850
H	-0.422153	-0.354370	-0.016354
N	-1.747627	1.675245	-0.643716
C	-1.868946	2.209736	1.777463
H	-0.143399	2.346174	0.475019
C	-3.067438	2.945692	1.155046
C	-3.087851	1.036899	-0.466684
C	-3.963091	1.886829	0.485318
H	-2.918803	0.024359	-0.080030
H	-3.529812	0.930950	-1.465467
H	-4.428564	1.242069	1.245806
H	-4.779691	2.376069	-0.067517
C	-2.598728	3.946614	0.079894
H	-3.631235	3.469084	1.939650
C	-1.945775	3.104454	-1.054123
H	-1.277660	2.866754	2.431504
H	-2.255967	1.403551	2.412326
H	-0.961131	3.515125	-1.330531
H	-2.571006	3.086746	-1.959483
C	5.864605	0.865744	0.674600
H	4.220199	0.182310	1.864184
C	6.407233	-0.401401	0.053462
H	6.404912	1.125510	1.596202
H	5.940550	1.710866	-0.021956
C	6.280892	-0.607451	-1.329411
C	6.728143	-1.799499	-1.904229
C	7.300976	-2.793755	-1.103467
C	7.432982	-2.591739	0.273894
C	6.987761	-1.398491	0.850269
H	7.105572	-1.238237	1.926725
H	5.841262	0.176234	-1.952952
H	6.637088	-1.949885	-2.982759
H	7.654222	-3.723901	-1.555151
H	7.891409	-3.361421	0.899461
C	-1.660483	5.054851	0.584479
H	-3.498419	4.436547	-0.328347
C	-2.255573	5.927023	1.689693
H	-1.394155	5.689457	-0.278944
H	-0.706759	4.614719	0.928344
H	-3.224160	6.353204	1.380581
H	-1.586673	6.766193	1.934119
H	-2.422899	5.359947	2.619252
H	-0.778047	-0.536724	5.494853

H	0.107873	0.725309	3.544121
C	-2.995650	-3.465601	3.001730
C	-3.354283	-3.976755	1.775256
C	-2.855457	-3.375140	0.585346
C	-2.019057	-2.268234	0.656641
H	-1.628176	-1.878199	-0.283130
H	-3.352979	-3.915908	3.929455
H	-4.009601	-4.845942	1.727138
O	-3.153211	-3.827365	-0.655524
C	-3.932080	-5.002188	-0.822385
H	-3.994275	-5.177846	-1.904227
H	-4.952852	-4.876683	-0.421807
H	-3.456526	-5.875500	-0.344182

System: Acetate-Ni-L
Ligand: Thiourea
Configuration: S
Total energy [Ha]: -3515.17971592324
Number of imaginary frequencies: 1 (-10.93 cm-1)
Multiplicity: 1
Charge: +1

Ni	1.4515330552	2.1170227343	-1.1660032315
C	2.7184693377	3.9762821476	-0.9958717761
O	1.4560589967	4.0370323355	-1.1154712906
O	3.1769535669	2.7728844112	-0.9766262354
C	3.5981670842	5.1632126478	-0.8562346069
H	3.0694110490	6.0637364545	-1.1937566581
H	4.5280935182	5.0121274823	-1.4225252800
H	3.8643780754	5.2786528621	0.2077122613
C	1.6111368884	-0.5703526371	0.1782339162
S	1.4086347352	-0.0255663699	-1.4943034531
N	0.5514750181	-0.9968657463	0.8645382488
N	2.8210175324	-0.5949568438	0.7333116767
C	-0.8559522020	-0.7119876063	0.5058480903
H	0.7119859213	-1.3966915173	1.7864550879
C	-1.7775489593	-1.7737073439	1.0686118887
C	-2.8675205051	-2.2675100429	0.274901172
C	-3.6749932154	-3.3108818966	0.8525747088
N	-3.4585391219	-3.8174752288	2.0949192906
C	-2.4707157705	-3.3281957382	2.8047325344
C	-1.6049066035	-2.3061068910	2.3328973049
C	-1.1371498606	0.7729217299	0.8727122159
H	-0.9042137683	-0.7594078868	-0.5862624113
N	-2.2633137264	1.3554001508	0.1409059901

C	-1.1910679532	1.1347935114	2.3753894765
H	-0.2564477394	1.2875107945	0.4532027212
C	-2.1060478744	2.3725693025	2.4971355238
C	-3.5461701119	1.1624885538	0.8468584554
C	-3.5482198628	1.9160332856	2.2085087185
H	-3.7115363297	0.0884741514	0.9872937114
H	-4.3497351317	1.5143274077	0.1830286693
H	-3.9128879358	1.2633088481	3.0172898953
H	-4.2154691379	2.7920478662	2.1791954577
C	-1.7141456624	3.4367170233	1.4394781291
H	-2.0409358971	2.7959617237	3.5095889124
C	-1.9978411162	2.7972854552	0.0342033343
H	-0.1809258725	1.3314989165	2.7697724519
H	-1.6178561811	0.3125804101	2.9711454677
H	-1.1301138608	2.9466185425	-0.6383790013
H	-2.8589470866	3.2713167270	-0.4592151276
C	4.1153466735	-0.3208695236	0.0797111233
H	2.8910056641	-0.9703969016	1.6780523962
C	4.8921272840	-1.5903669148	-0.1817010958
H	4.6737232074	0.3575613531	0.7414239728
H	3.9035786541	0.2319756008	-0.8440744820
C	4.4412011154	-2.5005591562	-1.1521758385
C	5.1423050753	-3.6841579304	-1.3860812065
C	6.3015309514	-3.9693575852	-0.6539365032
C	6.7561430277	-3.0678264319	0.3114794812
C	6.0513823350	-1.8827789871	0.5482893905
H	6.4131274024	-1.1769530512	1.3020831044
H	3.5407943230	-2.2751419945	-1.7310991820
H	4.7890832254	-4.3850085782	-2.1463926311
H	6.8513859326	-4.8950558916	-0.8402101141
H	7.6624516440	-3.2849832955	0.8817640557
C	-0.2768078830	3.9679247646	1.5639493217
H	-2.3902569083	4.2976258327	1.5781234567
C	0.0741746694	4.5716017128	2.9225324720
H	-0.1236133612	4.7198746438	0.7734025721
H	0.4399549897	3.1584885468	1.3222533910
H	-0.6294004347	5.3763557405	3.1924736267
H	1.0860978613	5.0072156996	2.9152491839
H	0.0442170495	3.8231078909	3.7300034806
H	-2.3185687761	-3.7426078811	3.8085634878
H	-0.8107267727	-1.9606977964	3.0023918658
C	-4.7564595843	-3.8335573214	0.0933307563
C	-5.0533520153	-3.3512265682	-1.1621387552
C	-4.2697874288	-2.3018023810	-1.7231736830
C	-3.1985247660	-1.7773069832	-1.0103910685

H -2.6634301362 -0.9411169836 -1.4612407515
H -5.3482990160 -4.6295374284 0.5487515070
H -5.8933377552 -3.7711383844 -1.7151098947
O -4.5052697667 -1.7548564949 -2.9333539752
C -5.5845901967 -2.2160229174 -3.7268913807
H -5.5661537665 -1.6197180463 -4.6482709133
H -6.5557987762 -2.0664452334 -3.2229878141
H -5.4729558369 -3.2827874807 -3.9892496165

System: Acetate-Ni-L
Ligand: Thiourea
Configuration: S
Total energy [Ha]: -3515.20694586586
Number of imaginary frequencies: 0
Multiplicity: 3
Charge: +1

Ni -2.1209586638 1.6515329552 1.1153621295
C -3.8520258861 2.5522728354 -0.1917910277
O -3.6684111901 2.8745807195 1.0274989434
O -3.0429697733 1.6966389999 -0.6851342995
C -4.9319485605 3.1784901941 -1.0106534312
H -5.7476276925 3.5287867464 -0.3654360380
H -5.2970598013 2.4747845961 -1.7703037790
H -4.5019502062 4.0495751710 -1.5328543870
C -1.3808430493 -0.9136130522 -0.0948887660
S -1.4564717076 -0.4455725679 1.6133975814
N -0.2220265760 -0.8846549402 -0.7565151814
N -2.4713019697 -1.3395219687 -0.7258190332
C 1.0955071076 -0.4078799959 -0.2771475150
H -0.2305517916 -1.2295249897 -1.7135821399
C 2.1947895449 -0.9673943334 -1.1588708693
C 3.4410934569 -1.3840482181 -0.5807412150
C 4.4218324441 -1.9375878047 -1.4798503336
N 4.2259452656 -2.0476579064 -2.8203492772
C 3.0907647575 -1.6253465569 -3.3219324288
C 2.0468674075 -1.0774240775 -2.5300184865
C 1.0544051941 1.1365435127 -0.1344233115
H 1.2228387776 -0.7997699342 0.7390952355
N 2.2195569759 1.6996269823 0.5510337879
C 0.6804160017 1.9704475718 -1.3812982313
H 0.2463834143 1.2687576511 0.5903209034
C 1.2473641748 3.3897594623 -1.1281790984
C 3.2703874081 2.1184584331 -0.3973022773
C 2.7782732463 3.3018004796 -1.2796107021

H 3.5471751944 1.2536848344 -1.0121456275
H 4.1654681253 2.3870236293 0.1830727030
H 3.0494792615 3.1432635419 -2.3352405884
H 3.2413029460 4.2530870969 -0.9721312928
C 0.9422539198 3.8441234730 0.3273039366
H 0.8298978871 4.1064475367 -1.8501235914
C 1.7389477413 2.8824433732 1.2780368298
H -0.4103878140 1.9729804195 -1.5400141345
H 1.1326966610 1.5611491590 -2.2982171832
H 1.1080630954 2.5497688136 2.1211025626
H 2.6053114072 3.3972853041 1.7186739044
C -3.8431825542 -1.4393638152 -0.2070301309
H -2.3576574817 -1.6555291137 -1.6871951735
C -4.2802630021 -2.8763644679 -0.0393523234
H -4.4949004992 -0.8999266574 -0.9102408099
H -3.8670330892 -0.8973858345 0.7462077845
C -3.6954086613 -3.6701157570 0.9609599776
C -4.0742091640 -5.0045005685 1.1112137933
C -5.0420295321 -5.5580596309 0.2637813242
C -5.6287503366 -4.7735307110 -0.7322644119
C -5.2464812442 -3.4362766708 -0.8848152818
H -5.7100456314 -2.8237189513 -1.6639408475
H -2.9435377107 -3.2354654310 1.6259800126
H -3.6188956455 -5.6153308500 1.8943659462
H -5.3400875095 -6.6023097338 0.3839492746
H -6.3869436465 -5.2013431363 -1.3923569498
C -0.5585637443 3.9316711447 0.6545708530
H 1.3365052189 4.8672449987 0.4486658242
C -1.3403176868 5.0155145018 -0.0805266685
H -0.6938812031 4.0409900267 1.7441933461
H -1.0022424976 2.9487902324 0.3441797633
H -0.9023978174 6.0050550666 0.1239924130
H -2.3879739429 5.0343551231 0.2527123595
H -1.3231515384 4.8591320208 -1.1698889477
H 2.9573768161 -1.7092804463 -4.4073030174
H 1.1401627520 -0.7421000546 -3.0434916772
C 5.6606341206 -2.3802470858 -0.9444896924
C 5.9434623066 -2.2696663025 0.3988972888
C 4.9860367083 -1.6941050250 1.2827217206
C 3.7600772124 -1.2615408401 0.7912947914
H 3.0835419582 -0.7780771360 1.4944721466
H 6.3844591442 -2.8051255710 -1.6422283544
H 6.9058970851 -2.6177913890 0.7734334812
O 5.1936424296 -1.5260168923 2.6047670509
C 6.4197604864 -1.9278475301 3.1900600337

H 6.3466142980 -1.6825586862 4.2574278190
H 7.2776672168 -1.3848501159 2.7556552402
H 6.5872401064 -3.0141071884 3.0833403832

System: Acetate-Ni-L
Ligand: Thiourea
Configuration: N
Total energy [Ha]: -3515.17753908909
Number of imaginary frequencies: 0
Multiplicity: 1
Charge: +1

Ni	-1.7023537662	-1.5321436296	0.2530593770
C	-2.0357153460	-2.8618408993	-1.5350954120
O	-2.6958992810	-2.9797079132	-0.4452070443
O	-1.1498065706	-1.9438545444	-1.5083611962
C	-2.2638019801	-3.7247164614	-2.7220154246
H	-3.2946271394	-4.1021534065	-2.7262899137
H	-1.5748104850	-4.5840145115	-2.6654655635
H	-2.0348026452	-3.1641196844	-3.6384912912
C	-0.9592194083	-0.7751235572	2.2881084080
S	-0.0125592945	-1.3200217068	3.4815433437
N	-0.5918100215	-0.1014768345	1.0716419869
N	-2.3331111698	-1.1471925097	2.0789083684
C	0.8437096756	0.1034199736	0.6936164237
H	-1.1178184807	0.7697999140	0.9371125979
C	0.9035904649	0.1802995591	-0.8203025128
C	1.8496181752	-0.6187583572	-1.542182452
C	1.7136087056	-0.6537139467	-2.9753008024
N	0.7805969925	0.0654215945	-3.6520771681
C	-0.0242475641	0.8403752259	-2.9656557205
C	-0.0024226311	0.9256584597	-1.5505534307
C	1.4505368346	1.2577820485	1.5283492807
H	1.3522507551	-0.8089860267	1.0228266032
N	2.9046816458	1.3360384349	1.3650310298
C	0.7889544466	2.6531880990	1.3808246321
H	1.3028862118	0.9321106820	2.5662832364
C	1.9106873157	3.6972350517	1.5889801531
C	3.2936700853	2.1461379702	0.1952306108
C	2.8374655183	3.6252415789	0.3614298119
H	2.8626549260	1.6965282793	-0.7065949187
H	4.3855406724	2.0684888312	0.0859040528
H	2.3070902300	3.9764172153	-0.5383558046
H	3.6987534973	4.2969121263	0.5048808748
C	2.7448001165	3.3550213782	2.8511071819

H	1.4770689932	4.7036476550	1.6765699886
C	3.4447442073	1.9780940795	2.5754109889
H	-0.0213988278	2.7837597876	2.1183350906
H	0.3519790067	2.8061021184	0.3810306352
H	3.3106225104	1.2937261387	3.4290638644
H	4.5284074973	2.1031917208	2.4350500081
C	-3.4284075305	-0.0948396110	2.0268047307
H	-2.5891288351	-1.9274651596	2.6891620383
C	-4.2369893262	-0.2527643262	0.7642148366
H	-2.9515195182	0.8949587157	2.0811373434
H	-4.0360770085	-0.2126049337	2.9350130372
C	-5.2967901690	-1.1671315912	0.7098066836
C	-5.9415456725	-1.4250977012	-0.5020820579
C	-5.5271351192	-0.7759711532	-1.6697173852
C	-4.4781199532	0.1481658877	-1.6223920705
C	-3.8415806707	0.4124879077	-0.4079278848
H	-3.0332930923	1.1483990246	-0.3764160431
H	-5.6134689490	-1.6902502725	1.6163957345
H	-6.7672432970	-2.1393054909	-0.5363545615
H	-6.0311399715	-0.9812455471	-2.6170882352
H	-4.1612502169	0.6691935677	-2.5285329567
C	1.9522773142	3.3582241529	4.1683545928
H	3.5196425351	4.1363515790	2.9375719356
C	1.2484240484	4.6769361174	4.4904377746
H	2.6552801868	3.1141940573	4.9840125151
H	1.2144046667	2.5342871296	4.1704362323
H	1.9575684222	5.5210210687	4.4744980149
H	0.7903362461	4.6481196241	5.4914669752
H	0.4468273894	4.9071782084	3.7697826792
H	-0.7551694636	1.4323046569	-3.5303703502
H	-0.7463979487	1.5630939535	-1.0666827473
C	2.6039762915	-1.4715075577	-3.7205123079
C	3.6001492908	-2.1969923589	-3.1047849445
C	3.7603187559	-2.1284754869	-1.6905046613
C	2.8934113972	-1.3508757442	-0.9311552058
H	3.0902126257	-1.2782927733	0.1387382606
H	2.4774934362	-1.4915580208	-4.8045484350
H	4.2705511747	-2.8071700925	-3.7097877603
O	4.7297771579	-2.7775838458	-1.0151280596
C	5.6901863669	-3.5518853572	-1.7113603107
H	6.3763274041	-3.9473829348	-0.9513288536
H	6.2655237296	-2.9412491987	-2.4294024697
H	5.2244921582	-4.3981566517	-2.2467581722

System: Acetate-Ni-L

Ligand: Thiourea
Configuration: N
Total energy [Ha]:
Number of imaginary frequencies:
Multiplicity: 3
Charge: +1

Could not converge but it is the least stable configuration.

System: Acetate-Ni-L
Ligand: Thiourea
Configuration: Lin
Total energy [Ha]: -3515.20463652888
Number of imaginary frequencies: 2 (-38.21 and -18.84 cm-1)
Multiplicity: 1
Charge: +1

Ni	2.6392450919	0.1045212280	0.1766441270
C	4.8493152243	-0.5702348605	0.2531064525
O	4.3520910021	-0.0032395798	-0.7721114068
O	4.0332548125	-0.7095661192	1.2337569649
C	6.2482740004	-1.0700251767	0.3091735666
H	6.8708606993	-0.5376744788	-0.4213589622
H	6.2326073435	-2.1418765823	0.0538252514
H	6.6470918877	-0.9638430571	1.3271378240
C	-0.0524359498	-1.3221161202	1.4254030180
S	0.9478831329	0.0613418880	1.7762941200
N	-1.3572873699	-1.1601606094	1.1216452824
N	0.4166422440	-2.5749332548	1.4997742042
C	-1.9777024262	0.0970214636	0.6367706270
H	-1.9344887756	-1.9958788796	1.0635205612
C	-1.0784865016	0.6605395847	-0.4603816869
C	-0.5803886154	2.0010312387	-0.340402037
C	0.7802857306	2.2031743844	-0.7480367304
N	1.5112321134	1.2003536747	-1.3323221818
C	0.8634669725	0.1197388793	-1.7649043753
C	-0.4526025619	-0.1852922203	-1.3620303214
C	-3.4768259780	-0.1545700592	0.3578132506
H	-1.9510189279	0.8195830577	1.4619639884
N	-4.1625897949	1.1006536123	0.0252382569
C	-3.8286083940	-1.2705294418	-0.6665777305
H	-3.8772456427	-0.4581962660	1.3389798034
C	-5.1131536688	-0.8201676876	-1.3984466056
C	-4.0361693616	1.4337190329	-1.4072647791
C	-4.7339902954	0.3677764260	-2.2998937300

H	-2.9717988292	1.5199880941	-1.6604975566
H	-4.4683051542	2.4344049001	-1.5547676042
H	-4.0653178786	0.0359798539	-3.1108571751
H	-5.6369841536	0.7765952733	-2.7801299187
C	-6.1833129491	-0.3355279565	-0.3874604132
H	-5.5097684348	-1.6457872799	-2.0056443784
C	-5.5936607696	0.9256185360	0.3351199949
H	-3.9764369398	-2.2412869026	-0.1652746192
H	-3.0313807767	-1.4093576114	-1.4146649760
H	-5.7096114057	0.8409009502	1.4278382070
H	-6.1177708969	1.8438572488	0.0317392428
C	1.7708879637	-2.9838226512	1.8609032205
H	-0.1982877690	-3.3262687507	1.1936818475
C	2.6146790485	-3.4101011151	0.6747916506
H	1.6965504436	-3.8024250445	2.5943111111
H	2.2553205419	-2.1326383122	2.3631943497
C	2.3095830612	-3.0072728630	-0.6308240654
C	3.1619907851	-3.3218965201	-1.6940828824
C	4.3253012451	-4.0589728013	-1.4633319559
C	4.6273341285	-4.4823285733	-0.1631060434
C	3.7790306432	-4.1589142262	0.8982822740
H	4.0303391084	-4.4834621102	1.9120362138
H	1.4028608938	-2.4323167810	-0.8203093297
H	2.9151801730	-2.9916919884	-2.7062599661
H	4.9906115327	-4.3103820937	-2.2924158142
H	5.5292514051	-5.0703799282	0.0246439543
C	-6.6679010342	-1.4022727693	0.6094074559
H	-7.0600238420	-0.0268915623	-0.9820503535
C	-7.2535978784	-2.6601719290	-0.0320265269
H	-7.4305285988	-0.9325894079	1.2547667703
H	-5.8456898580	-1.6821623496	1.2938470882
H	-8.0583038188	-2.4064685964	-0.7419083849
H	-7.6815698248	-3.3316496276	0.7284329856
H	-6.4935604618	-3.2341853377	-0.5865463223
H	1.4554463527	-0.6067276904	-2.3304588234
H	-0.8230674473	-1.1962796106	-1.5392676600
C	1.4063813845	3.4350492145	-0.4508797251
C	0.7053172495	4.4533403206	0.1638019906
C	-0.6581586620	4.2664330095	0.5336790086
C	-1.2780675917	3.0389450633	0.3016975833
H	-2.3195307798	2.9064450539	0.6024936167
H	2.4490262805	3.5669097999	-0.7465005814
H	1.2038431215	5.4030756997	0.3557910150
O	-1.4089949561	5.2125527776	1.1175893378
C	-0.8646714359	6.4926490911	1.4013007789

H -1.6717815214 7.0717428616 1.8670116174
H -0.5374557445 7.0068428996 0.4811278093
H -0.0171649008 6.4262144099 2.10530459826

System: Acetate-Ni-L
Ligand: Thiourea
Configuration: Lin
Total energy [Ha]: -3515.22331825367
Number of imaginary frequencies: 0
Multiplicity: 3
Charge: +1

Ni 2.6619095835 -0.3103831362 0.3612870729
C 4.6420523810 -1.5826380239 0.0104189646
O 4.0765346907 -1.5640826870 1.1485546984
O 4.1200917907 -0.8874729050 -0.9180687533
C 5.9021144851 -2.3590455206 -0.2220530200
H 5.9924043501 -3.1751217151 0.5060944535
H 6.7574362289 -1.6763494242 -0.0873794658
H 5.9335753617 -2.7386711927 -1.2521653164
C 0.3168056520 1.7344545431 -1.2550334290
S 1.0955530401 0.2249309166 -1.5174697287
N -1.0130464308 1.8257237348 -1.0301244829
N 1.0263133479 2.8818943273 -1.2972818224
C -1.8424957342 0.6957592251 -0.5544321929
H -1.4306055488 2.7510498087 -0.9718869546
C -1.1484520160 0.1144446192 0.6768060956
C -0.7743036879 -1.2693541715 0.6935189470
C 0.4786255469 -1.5741714784 1.3262803356
N 1.2092826894 -0.6200992812 1.9896547546
C 0.6300243778 0.5451370406 2.2400245133
C -0.5564189902 0.9559232749 1.6044118015
C -3.3193745669 1.1387095729 -0.4744313135
H -1.8067770425 -0.0780765041 -1.3312075602
N -4.1915969802 -0.0060077788 -0.1795709057
C -3.6468357673 2.3443670260 0.4541339623
H -3.5615884531 1.4306907468 -1.5092055622
C -5.0274420226 2.0715699107 1.0894735613
C -4.2551460885 -0.2989830305 1.2651643768
C -4.8673566898 0.8897758944 2.0607993001
H -3.2463721683 -0.5334164486 1.6284364992
H -4.8456748851 -1.2189997626 1.3909849766
H -4.2168381831 1.1754252277 2.9036505040
H -5.8458338153 0.6221856148 2.4891037474
C -6.0571185719 1.6732818290 0.0025567444

H -5.3726016161 2.9629308131 1.6318564276
C -5.5488399481 0.3366885291 -0.6428697250
H -3.6555689326 3.2893745566 -0.1135440041
H -2.9054139425 2.4575109770 1.2606203244
H -5.5341601933 0.4150617336 -1.7420662195
H -6.2116894766 -0.5049561208 -0.3936069269
C 2.4225720583 2.9789995479 -1.7183034990
H 0.5493155606 3.7540884172 -1.0871673288
C 3.4482824428 2.4568300010 -0.7237133963
H 2.6168040840 4.0431972282 -1.9260806626
H 2.5450805239 2.4319469408 -2.6666633662
C 3.0960549192 2.1144011571 0.5976105225
C 4.0552671056 1.5906428610 1.4822041866
C 5.3846364654 1.4349167148 1.0628432298
C 5.7335013223 1.7862565857 -0.2391293497
C 4.7707807353 2.2833837209 -1.1294199164
H 5.0600951502 2.5192507436 -2.1569897198
H 2.0857198142 2.3244101241 0.9490292090
H 3.7696851643 1.3283745740 2.5033593854
H 6.1271946830 1.0264170314 1.7503984228
H 6.7631259265 1.6591899351 -0.5811726257
C -6.3340104015 2.7601121412 -1.0488895408
H -7.0096576907 1.4759958732 0.5244886703
C -6.8553739722 4.0806916365 -0.4811902415
H -7.0696171249 2.3569268077 -1.7668822143
H -5.4214267511 2.9468405137 -1.6453017200
H -7.7619695829 3.9218616091 0.1260249066
H -7.1135584603 4.7848838494 -1.2871492781
H -6.1104461592 4.5763114000 0.1623482408
H 1.1975571102 1.2545783657 2.8513828835
H -0.8347087186 2.0085299934 1.6693045909
C 1.0347865194 -2.8681357418 1.1597422687
C 0.3489125724 -3.8423592438 0.4617143990
C -0.9214781766 -3.5561963300 -0.1175548212
C -1.4582683020 -2.2727046482 -0.0141774842
H -2.4253435494 -2.0654370487 -0.4770968884
H 2.0082724212 -3.0671757882 1.6123300851
H 0.7871067530 -4.8355147338 0.3678928435
O -1.6551429882 -4.4551510909 -0.7890904126
C -1.1947440641 -5.7882748856 -0.9520231048
H -1.9741454011 -6.3135377250 -1.5179430655
H -1.0555440303 -6.2904455849 0.0208820268
H -0.2505616049 -5.8239305405 -1.5225399168

System: Acetate-Ni-L

Ligand: Thiourea
Configuration: Din
Total energy [Ha]: -3515.25076227006
Number of imaginary frequencies: 1 (-13.74 cm-1)
Multiplicity: 1
Charge: +1

Ni	-0.1188258004	-1.2592648436	1.5836666727
C	-0.7089034002	-0.4934201854	3.7514926150
O	-1.5871448390	-0.8665145947	2.8972349937
O	0.5007689205	-0.5600582541	3.3611018710
C	-1.0828310519	-0.0406769115	5.1221502723
H	-2.1004419863	0.3714070691	5.1209243992
H	-0.3550120045	0.6952319439	5.4894813098
H	-1.0590373096	-0.9123284035	5.7969615060
C	2.3770524733	-0.7670583962	-0.5710508487
S	1.9393239526	-1.7202940193	0.8040564665
N	1.4881946249	-0.1223279572	-1.3525514673
N	3.6684415564	-0.6800093829	-0.9205390207
C	0.0333056575	-0.0751345665	-1.1663617145
H	1.8558983628	0.5315910294	-2.0384164597
C	-0.5395827173	0.9872365482	-2.0818283912
C	-1.3460453999	2.0506000146	-1.558413960
C	-1.8537980836	3.0034579997	-2.5145045113
N	-1.5938696237	2.9326962031	-3.8449730275
C	-0.8424127181	1.9515121815	-4.2882722055
C	-0.2947396419	0.9534012764	-3.4428313327
C	-0.5840309657	-1.5147338169	-1.2718870795
H	-0.1407576942	0.2590293574	-0.1336565976
N	-1.1923469770	-1.8976598362	0.0556653278
C	-1.5992432020	-1.8117089797	-2.3994388636
H	0.2675165907	-2.1944368854	-1.3847454296
C	-2.6466008714	-2.8066008661	-1.8675256804
C	-2.5889499399	-1.3437073364	0.1539489879
C	-3.5211692760	-2.0495725500	-0.8560436959
H	-2.5235110654	-0.2655466838	-0.0354991907
H	-2.9199115544	-1.4636898934	1.1906215206
H	-4.1510302262	-1.3042652589	-1.3645145723
H	-4.1984740783	-2.7478057990	-0.3408251935
C	-1.9729441279	-3.9838752844	-1.1274492182
H	-3.2621795826	-3.1824078457	-2.6959634093
C	-1.2620087074	-3.3935212806	0.1262370134
H	-1.0677875999	-2.2057026562	-3.2775087392
H	-2.1128029162	-0.8977828191	-2.7272057507
H	-0.2320707529	-3.7677814788	0.2180339579

H	-1.7881845972	-3.6373900170	1.0597427293
C	4.8385071516	-1.0695308204	-0.1132685030
H	3.8870277566	-0.0896714195	-1.7197073180
C	5.4512688413	0.1229357904	0.5871003465
H	5.5613222830	-1.5406377893	-0.7958911878
H	4.5142213432	-1.8355821573	0.6022079345
C	4.8317860675	0.6699201376	1.7238590756
C	5.3715797301	1.8002591236	2.3403135251
C	6.5319279786	2.3947040864	1.8296265265
C	7.1537141942	1.8535628769	0.7016086260
C	6.6131523069	0.7220454402	0.0824705097
H	7.1072292607	0.2962165569	-0.7962259976
H	3.9282828910	0.2036045388	2.1270303028
H	4.8899065967	2.2163254471	3.2286576559
H	6.9544175581	3.2768781323	2.3167941180
H	8.0634579512	2.3096082474	0.3038563063
C	-1.0190672672	-4.8323354711	-1.9847239529
H	-2.7796982590	-4.6499371289	-0.7788366860
C	-1.6733215088	-5.4672244587	-3.2115291147
H	-0.6031211108	-5.6254146719	-1.3396127892
H	-0.1516709277	-4.2233902496	-2.2959559426
H	-2.5610930375	-6.0563257181	-2.9281172653
H	-0.9741683277	-6.1455248830	-3.7236583842
H	-1.9961216855	-4.7131155551	-3.9470079318
H	-0.6471921852	1.9158249549	-5.3665833902
H	0.2982366552	0.1475466370	-3.8848209556
C	-2.6613729967	4.0751080932	-2.044943350
C	-2.9641245073	4.2191400290	-0.7097543412
C	-2.4617534930	3.2807624895	0.2376593460
C	-1.6664482743	2.2241776526	-0.1891566134
H	-1.3069882469	1.5467932485	0.5860353757
H	-3.0316470644	4.7837556205	-2.7880578230
H	-3.5850420575	5.0550138609	-0.3878568726
O	-2.7080649884	3.3444377871	1.5610943154
C	-3.5109651953	4.3888636060	2.0876400844
H	-3.5692585196	4.2158162707	3.1698378122
H	-4.5311171891	4.3709027490	1.6663707348
H	-3.0597145111	5.3799783929	1.90718804017

System: Acetate-Ni-L
Ligand: Thiourea
Configuration: Din
Total energy [Ha]: -3515.26222043816
Number of imaginary frequencies: 1 (-8.98 cm-1)
Multiplicity: 3

Charge: +1

Ni 0.1708718406 1.0466000038 -1.9544948008
C 0.2496687626 -1.0580758926 -3.0421007754
O -0.2880808881 -0.0490673781 -3.5963714570
O 0.7735689895 -0.8776929805 -1.8888419232
C 0.2493433114 -2.4161474788 -3.6653083453
H -0.2680110214 -2.4040739613 -4.6317194230
H -0.2294989997 -3.1199237602 -2.9650813873
H 1.2897357797 -2.7523111061 -3.7946349658
C 1.6937915753 1.8126019716 0.8607459441
S 1.4469107539 2.5039562089 -0.7117957082
N 0.7989791212 1.0441384366 1.5101920161
N 2.8195647772 2.1179367525 1.5212417987
C -0.3857613100 0.3360026509 0.9801385983
H 1.0965765547 0.6853040670 2.4140892620
C -0.8639044725 -0.6262510269 2.0540794417
C -1.0811065838 -2.0128399593 1.7622054640
C -1.6352851548 -2.8172351338 2.8225163390
N -1.9147442221 -2.3291978341 4.0585176095
C -1.6661551309 -1.0635864272 4.3034272948
C -1.1479071992 -0.1719827053 3.3295227904
C -1.4851734421 1.3257650760 0.4440398153
H -0.0402489661 -0.2633764631 0.1335063791
N -1.6290942108 1.1859710692 -1.0491615591
C -2.8946567140 1.2640845314 1.0833399627
H -1.0831344242 2.3347798502 0.5894150161
C -3.9472871388 1.5373195053 -0.0081001033
C -2.5160559301 0.0310478354 -1.3927603065
C -3.9662209739 0.3102746105 -0.9344165497
H -2.0960387493 -0.8594963529 -0.9087192383
H -2.4390321742 -0.1226812122 -2.4759814156
H -4.3686036211 -0.5655525454 -0.4033765786
H -4.6227248077 0.4929605354 -1.7990632377
C -3.5707468308 2.7795140906 -0.8466914761
H -4.9355888643 1.6752926368 0.4515900625
C -2.2403664343 2.4399775401 -1.5842702992
H -2.9564627793 1.9879916548 1.9086101701
H -3.0945064112 0.2767035259 1.5198432245
H -1.5039443018 3.2504142861 -1.4629696301
H -2.3939869918 2.2841174371 -2.6623205999
C 4.0290020995 2.7472047403 0.9698482859
H 2.9554015739 1.6750691705 2.4275100083
C 5.0827958131 1.7222053312 0.6008255216
H 4.4124390114 3.4501798326 1.7239174184

H 3.7276825686 3.3386199240 0.0951353325
C 4.7893892743 0.7050141716 -0.3241763204
C 5.7635443488 -0.2312410912 -0.6732649373
C 7.0421163825 -0.1616786932 -0.1068510738
C 7.3401251621 0.8464678565 0.8126136261
C 6.3621533516 1.7820964823 1.1677762854
H 6.6024202885 2.5701978541 1.8874931509
H 3.7976354994 0.6478411132 -0.7810467653
H 5.5268532838 -1.0165570227 -1.3953519819
H 7.8048594800 -0.8932032677 -0.3842815701
H 8.3358619313 0.9062520283 1.2583754578
C -3.4725822622 4.0935541633 -0.0543095173
H -4.3595274198 2.9110434618 -1.6060269505
C -4.7651406437 4.5010971918 0.6518469608
H -3.1755276399 4.8903600246 -0.7587657215
H -2.6493210359 4.0325684224 0.6803692571
H -5.6096260467 4.5417662884 -0.0556877073
H -4.6665479070 5.4977842652 1.1081799798
H -5.0385726979 3.7997126116 1.4564113810
H -1.8924608293 -0.6931306098 5.3103637108
H -1.0197174069 0.8822947829 3.5916005706
C -1.8949665887 -4.1917277337 2.5695631063
C -1.6214300615 -4.7647244448 1.3473989810
C -1.0528540547 -3.9752657136 0.3077110162
C -0.7841274993 -2.6299383162 0.5211666713
H -0.2953431882 -2.0907128592 -0.2896407784
H -2.3183066070 -4.7784382980 3.3868138240
H -1.8344400171 -5.8219791173 1.1914499254
O -0.7418961442 -4.4611935208 -0.9173790108
C -0.9047377197 -5.8435932352 -1.1962887602
H -0.5571782576 -5.9925546212 -2.2266807163
H -1.9620727266 -6.1527361703 -1.1269882418
H -0.2973286958 -6.4679601222 -0.5187025363

System: Acetate-Ni-L
Ligand: Thiosquaramide
Configuration: S
Total energy [Ha]: -4027.49300289053
Number of imaginary frequencies: 0
Multiplicity: 1
Charge: +1

Ni 3.2996634546 2.2247250016 -1.0584513922
C 4.3610605646 3.9255968706 -2.1013167212
O 3.0905682522 3.8191262572 -2.0888748407

O 4.9850637418 2.9955285771 -1.4868911289
C 5.0702687910 5.0515771592 -2.7687704795
H 4.3558698560 5.6962910796 -3.2954188745
H 5.8171588851 4.6476355192 -3.4688382675
H 5.6145559790 5.6327072414 -2.0077058843
C 0.6788444813 -0.7829721335 0.7513013316
C 1.9808827080 -1.3194452501 1.1424561680
C 2.6308654933 -0.2067753935 0.4791161770
C 1.3471435604 0.3180271302 0.0765631940
S 1.0951250470 1.6952914627 -0.8339256042
S 4.1197003268 0.4631295457 0.1236930016
N -0.5617208787 -1.1674704579 0.9348530007
N 2.3824940165 -2.3763955494 1.8148072490
C -1.7583425931 -0.4568818237 0.4136229646
H -0.7343938394 -2.0289534784 1.4460445399
C -2.9126315216 -1.4399995432 0.3618906215
C -3.7957868087 -1.4662901417 -0.7688492122
C -4.7893898038 -2.5100613264 -0.7918266475
N -4.9472145746 -3.4115166756 0.2130432114
C -4.1622440618 -3.3235107731 1.2593785998
C -3.1280030850 -2.3577286469 1.3750691771
C -1.9513323691 0.8677718974 1.2074816482
H -1.5112815746 -0.1518734154 -0.6113368732
N -2.9774684498 1.7232383815 0.6032655143
C -2.1407601083 0.7553228762 2.7397536088
H -1.0077345798 1.4047068856 1.0341506712
C -3.0065669033 1.9628213093 3.1636766777
C -4.3294727546 1.4207851879 1.1135498029
C -4.4330052520 1.7145861293 2.6387065522
H -4.5684222334 0.3736117014 0.8959628402
H -5.0439536331 2.0243955372 0.5342019365
H -4.8959212316 0.8671572878 3.1697873059
H -5.0615019732 2.5974149910 2.8360793434
C -2.4771496570 3.2699153603 2.5161506061
H -3.0176109846 2.0548705025 4.2592897641
C -2.6483596771 3.1130351224 0.9649036925
H -1.1667014603 0.7561993626 3.2543007364
H -2.6598519260 -0.1732598895 3.0262953938
H -1.7251768548 3.4050498686 0.4377740404
H -3.4517250840 3.7608032566 0.5840991044
C 3.7996954998 -2.7837775801 1.9553368005
H 1.6868813469 -3.0626013110 2.0979956270
C 4.1684609856 -3.8555918833 0.9552875894
H 3.9374714134 -3.1337981417 2.9885681864
H 4.4096431082 -1.8792778197 1.8238373299

C 4.4299351490 -3.5056005519 -0.3792530442
C 4.7257179030 -4.4936388953 -1.3196250563
C 4.7640312611 -5.8389744614 -0.9342993453
C 4.5093701037 -6.1930507799 0.3936199625
C 4.2108209925 -5.2040221676 1.3360489324
H 4.0227886543 -5.4854300989 2.3767291131
H 4.4149779256 -2.4535701791 -0.6786900537
H 4.9370640089 -4.2141586937 -2.3544822593
H 5.0010233903 -6.6112445260 -1.6698808682
H 4.5487165206 -7.2411875381 0.6992606480
C -1.0390727256 3.6501382495 2.9072983182
H -3.1330429804 4.0858284014 2.8657882698
C -0.8121961828 3.8383250672 4.4071909524
H -0.7865641877 4.5863700349 2.3792069729
H -0.3289638290 2.8979788335 2.5157427894
H -1.5278775873 4.5635778184 4.8285924419
H 0.2020016298 4.2153679387 4.6125373141
H -0.9315670451 2.8956206769 4.9652030529
H -4.3209388927 -4.0443604616 2.0705096719
H -2.5198299554 -2.3671477573 2.2849339025
C -5.6585092529 -2.5949318479 -1.9116876990
C -5.5894138057 -1.6865488594 -2.9444425170
C -4.6393149728 -0.6257865328 -2.8977311529
C -3.7588692265 -0.5280434148 -1.8260273954
H -3.0957764438 0.3365864133 -1.8003076953
H -6.3928830159 -3.4024390890 -1.9159019147
H -6.2763945810 -1.7802244899 -3.7852285067
O -4.5375701645 0.3274520292 -3.8452936614
C -5.4199992429 0.3290212286 -4.9539741876
H -5.1497620929 1.2027893337 -5.5608831340
H -6.4736820060 0.4244634997 -4.6377801846
H -5.3067809845 -0.5814769997 -5.5684055333

System: Acetate-Ni-L
Ligand: Thiosquaramide
Configuration: S
Total energy [Ha]: -4027.48964349814
Number of imaginary frequencies: 2 (-40.17 and -4.56 cm-1)
Multiplicity: 3
Charge: +1

Ni 3.2716698940 2.3101864560 -1.2295766176
C 4.2953263776 4.1415133969 -2.3065327496
O 3.0296746225 4.0033261835 -2.3059641064
O 4.9796821241 3.2479911747 -1.7039467723

C	4.9634429343	5.3012690376	-2.9709539794
H	4.2339428211	5.9164467711	-3.5109910913
H	5.7422501925	4.9295204256	-3.6535759009
H	5.4704970158	5.9051431287	-2.2016819699
C	0.7269372481	-0.7592374516	0.7266740579
C	2.0454883539	-1.2596193364	1.1087240409
C	2.6667986608	-0.1591302506	0.3966986064
C	1.3562767807	0.3332007664	0.0038008520
S	0.9831295644	1.6650775055	-0.9285117339
S	4.1667342801	0.4742162051	0.0279237959
N	-0.5025962419	-1.1562216910	0.9449276376
N	2.4702538585	-2.2874098888	1.8085584977
C	-1.7149225141	-0.4705331542	0.4263133826
H	-0.6547953471	-1.9975240261	1.4948837069
C	-2.8355292279	-1.4876650716	0.3247407070
C	-3.6905608371	-1.5132189446	-0.8276785753
C	-4.6522276022	-2.5843036779	-0.8985917592
N	-4.8041813956	-3.5155813313	0.0796036348
C	-4.0449845704	-3.4314256982	1.1450704194
C	-3.0445121689	-2.4374391287	1.3089680894
C	-1.9559695689	0.8228378880	1.2582445894
H	-1.4580425027	-0.1265981962	-0.5831807841
N	-2.9821606271	1.6789488868	0.6559016153
C	-2.1857200321	0.6498133389	2.7800271779
H	-1.0175473873	1.3812892713	1.1275558030
C	-3.1157747817	1.8016461779	3.2203510381
C	-4.3445225030	1.3213477489	1.0966615399
C	-4.5101738332	1.5198247208	2.6317378067
H	-4.5541157622	0.2861305611	0.8051393634
H	-5.0489102312	1.9469338177	0.5285720292
H	-4.9480160124	0.6224292046	3.0979974571
H	-5.1875309726	2.3587193769	2.8569878467
C	-2.6173681129	3.1571686254	2.6533764351
H	-3.1723415854	1.8422278773	4.3176763708
C	-2.7032759133	3.0590238272	1.0901067180
H	-1.2283762507	0.6730560665	3.3247200140
H	-2.6725089971	-0.3091363993	3.0192060032
H	-1.7613358135	3.3921276845	0.6245858936
H	-3.5002683184	3.7048991120	0.6930763867
C	3.8915895259	-2.6862820394	1.9439644562
H	1.7842239915	-2.9663141411	2.1310524698
C	4.2321823202	-3.8167654372	1.0005726393
H	4.0482801941	-2.9757398322	2.9931415514
H	4.4986653825	-1.7930625690	1.7451285335
C	4.5363830818	-3.5404231915	-0.3419703465

C	4.7997726861	-4.5845601121	-1.2305087599
C	4.7590090991	-5.9110855530	-0.7852889448
C	4.4596579985	-6.1919761903	0.5509935804
C	4.1962075719	-5.1471930345	1.4419349904
H	3.9745419098	-5.3696219392	2.4902586987
H	4.5799332433	-2.5033536360	-0.6877451001
H	5.0461675910	-4.3642779968	-2.2718814285
H	4.9693203370	-6.7269671250	-1.4808422638
H	4.4381453645	-7.2260968318	0.9027300613
C	-1.2200928527	3.5835347949	3.1352595820
H	-3.3278544433	3.9270334938	3.0007465272
C	-1.0844294191	3.7316874936	4.6507222125
H	-0.9778124775	4.5460393283	2.6514607614
H	-0.4593540933	2.8722246684	2.7626012520
H	-1.8470883905	4.4200801125	5.0509455744
H	-0.0968842810	4.1344675116	4.9253872569
H	-1.2036932924	2.7687539636	5.1729490773
H	-4.1985917354	-4.1778920942	1.9336382116
H	-2.4563361039	-2.4529255046	2.2319230630
C	-5.4955073703	-2.6645337414	-2.0383978040
C	-5.4312264375	-1.7284920240	-3.0464574078
C	-4.5120274557	-0.6435589638	-2.9535958646
C	-3.6580072590	-0.5480083839	-1.8605163264
H	-3.0199900257	0.3337344631	-1.8000085481
H	-6.2065048577	-3.4917015236	-2.0782616046
H	-6.0983261213	-1.8190333316	-3.9034402820
O	-4.4164122106	0.3350610555	-3.8753009467
C	-5.2739254871	0.3403695722	-5.0035110882
H	-5.0140082197	1.2356816999	-5.5829227983
H	-6.3364811725	0.4001886111	-4.7091336760
H	-5.1224348801	-0.5514574278	-5.6367220713

System: Acetate-Ni-L
Ligand: Thiosquaramide
Configuration: N
Total energy [Ha]: -4027.43118806732
Number of imaginary frequencies: 2 (-13.65 and -11.09 cm-1)
Multiplicity: 1
Charge: +1

Ni	1.495746	0.608010	-0.107804
C	2.296603	2.778425	-0.223742
O	2.669468	1.935903	0.664546
O	1.448888	2.316442	-1.061839
C	2.813465	4.169773	-0.293278

H	3.325953	4.432951	0.640222
H	1.984477	4.861193	-0.501676
H	3.524746	4.236373	-1.132228
C	0.972624	-1.509700	0.277722
C	2.320583	-1.968441	0.220956
C	2.275196	-2.309745	1.683255
C	0.820483	-1.846807	1.710300
S	-0.335390	-1.800544	2.840977
S	3.295400	-2.932621	2.764067
N	0.239204	-0.794056	-0.677379
N	3.215414	-2.101639	-0.737234
C	-1.183763	-0.404860	-0.364180
H	0.336453	-1.143110	-1.636100
C	-1.487783	0.869911	-1.125064
C	-2.086766	1.978238	-0.439578
C	-2.178293	3.218430	-1.165146
N	-1.773562	3.356666	-2.454844
C	-1.282564	2.307938	-3.069959
C	-1.115712	1.045844	-2.443466
C	-2.105676	-1.635622	-0.509951
H	-1.173690	-0.174027	0.706181
N	-3.449711	-1.357852	0.001402
C	-2.157803	-2.311173	-1.905738
H	-1.674334	-2.357063	0.201475
C	-3.586512	-2.878130	-2.069256
C	-4.316049	-0.730303	-1.015972
C	-4.542764	-1.683479	-2.226195
H	-3.862547	0.214416	-1.336479
H	-5.264123	-0.466359	-0.524657
H	-4.353063	-1.160692	-3.177805
H	-5.582917	-2.043801	-2.262124
C	-4.005397	-3.671671	-0.803884
H	-3.637353	-3.521268	-2.959368
C	-4.045391	-2.650421	0.385613
H	-1.398052	-3.105799	-1.988469
H	-1.972448	-1.591178	-2.719725
H	-3.499215	-3.043434	1.258511
H	-5.077883	-2.461361	0.714643
C	4.681455	-2.112326	-0.499800
H	2.941544	-1.768144	-1.659975
C	5.172518	-0.686589	-0.372272
H	5.140284	-2.633982	-1.351378
H	4.868422	-2.697223	0.409566
C	5.401425	-0.120790	0.890105
C	5.776480	1.220058	1.001295

C	5.917233	2.007503	-0.144879
C	5.684926	1.452626	-1.407972
C	5.314703	0.110066	-1.520257
H	5.158742	-0.326863	-2.512304
H	5.290885	-0.733180	1.788416
H	5.958139	1.651610	1.987989
H	6.215842	3.054724	-0.054007
H	5.809502	2.059668	-2.308082
C	-3.131895	-4.898946	-0.495987
H	-5.029015	-4.041412	-0.987467
C	-3.088349	-5.946225	-1.608777
H	-3.519576	-5.363329	0.427583
H	-2.103419	-4.577370	-0.247972
H	-4.104806	-6.265452	-1.892502
H	-2.535739	-6.843488	-1.289335
H	-2.597329	-5.563979	-2.518196
H	-0.974871	2.435628	-4.114674
H	-0.647096	0.245602	-3.022419
C	-2.727327	4.350142	-0.505407
C	-3.189581	4.273291	0.790130
C	-3.132007	3.035699	1.494166
C	-2.588994	1.912411	0.880639
H	-2.626857	0.972127	1.431345
H	-2.780209	5.282537	-1.070613
H	-3.611299	5.160388	1.262383
O	-3.588369	2.868377	2.751419
C	-4.196927	3.946865	3.439568
H	-4.489130	3.558478	4.423693
H	-5.098576	4.309779	2.915401
H	-3.496836	4.789043	3.581223

System: Acetate-Ni-L
Ligand: Thiosquaramide
Configuration: N
Total energy [Ha]: -4027.46464638087
Number of imaginary frequencies: 1 (-21.69 cm-1)
Multiplicity: 3
Charge: +1

Ni	-0.7679388011	-1.0767382599	-0.9075523967
C	-2.7101271325	-1.8275019190	-1.9972913810
O	-2.0303546510	-2.5776820959	-1.2308750461
O	-2.2786384322	-0.6235207280	-2.1554583346
C	-3.9567157584	-2.2892972903	-2.6669625082
H	-4.0269232408	-3.3829625942	-2.6380683280

H	-3.9894415083	-1.9137680275	-3.6992666156
H	-4.8148336480	-1.8597430384	-2.1250059929
C	-0.8655862237	1.2508757360	0.6327248667
C	-2.2497032480	1.5286590443	0.5925992045
C	-2.2161924045	1.9080883937	2.0400979455
C	-0.7310916975	1.5668652158	2.0435742671
S	0.4422909754	1.5552170196	3.1784542566
S	-3.2801129274	2.4231858525	3.1426133546
N	-0.0104223545	0.7364822669	-0.3637695255
N	-3.1858989233	1.4526748147	-0.3366472728
C	1.3919479821	0.3576445589	0.0250708555
H	-0.0182901933	1.3339537495	-1.1994443516
C	1.6878481964	-0.9383773379	-0.7178675290
C	2.3002561437	-2.0437028751	-0.0441310044
C	2.3424202574	-3.3023775237	-0.7561891917
N	1.8731097192	-3.4615900958	-2.0223613991
C	1.3642687882	-2.4281711986	-2.6433075437
C	1.2291003842	-1.1418175752	-2.0342242358
C	2.3464387950	1.5624704018	-0.0959441680
H	1.3451352927	0.1284896121	1.0958854168
N	3.6950200190	1.2426261606	0.3772895920
C	2.3862224386	2.2778189661	-1.4734646555
H	1.9359065679	2.2633808131	0.6471753245
C	3.8202089102	2.8299144159	-1.6433117908
C	4.5243798558	0.6366398238	-0.6806568041
C	4.7572735395	1.6303019365	-1.8557891974
H	4.0371976761	-0.2813695612	-1.0343301648
H	5.4730821688	0.3207249205	-0.2220036503
H	4.5551225632	1.1486583461	-2.8264761558
H	5.8025122812	1.9756327213	-1.8836337391
C	4.2676568731	3.5753073311	-0.3578302186
H	3.8643968893	3.5031266079	-2.5110019116
C	4.3222730573	2.5123899422	0.7940648113
H	1.6390627164	3.0864889550	-1.5205429877
H	2.1750260010	1.5874188618	-2.3092073786
H	3.8023940983	2.8791972509	1.6930388190
H	5.3593893958	2.2948384502	1.0891809535
C	-4.6274385330	1.5527002106	-0.0726912632
H	-2.9320873181	0.9789260658	-1.2104933550
C	-5.2754172194	0.1818603323	-0.0154561375
H	-5.0826458591	2.1627467553	-0.8667617927
H	-4.7577744673	2.0892355458	0.8788790246
C	-4.7134611239	-0.8367548437	0.7720307551
C	-5.3132842311	-2.0958114842	0.8417346322
C	-6.4930311999	-2.3493797597	0.1324613522

C	-7.0636763567	-1.3397593154	-0.6480788861
C	-6.4528985661	-0.0827205134	-0.7275719531
H	-6.9019079473	0.7001963168	-1.3451661865
H	-3.8030769821	-0.6440535663	1.3467264171
H	-4.8632659014	-2.8790573817	1.4560418323
H	-6.9668617972	-3.3320490038	0.1903286772
H	-7.9854112789	-1.5307779721	-1.2029429353
C	3.4082373247	4.7974923069	0.0054859219
H	5.2909212256	3.9430777020	-0.5472479027
C	3.3529236524	5.8803828231	-1.0725103040
H	3.8149359694	5.2300121362	0.9362337743
H	2.3821026370	4.4763936889	0.2613410002
H	4.3662964597	6.2038664549	-1.3626312563
H	2.8098756578	6.7687237087	-0.7148096481
H	2.8441082042	5.5305332586	-1.9852647462
H	1.0141256521	-2.5719747932	-3.6717706037
H	0.8899862646	-0.3076170506	-2.6564661725
C	2.9117872580	-4.4243535325	-0.1029929690
C	3.4415236752	-4.3186705926	1.1651698632
C	3.4327345005	-3.0667952544	1.8519823526
C	2.8598751165	-1.9503384586	1.2491031572
H	2.9156416769	-0.9980261614	1.7773297761
H	2.9253477626	-5.3709836259	-0.6456861331
H	3.8770500502	-5.2003733717	1.6352454376
O	3.9564245800	-2.8837598139	3.0707755378
C	4.5774660573	-3.9577711292	3.7638420825
H	4.9260560122	-3.5432113057	4.7177734128
H	5.4426276860	-4.3538994054	3.2049119742
H	3.8635928522	-4.7746223612	3.9658798888

System: Acetate-Ni-L
Ligand: Thiosquaramide
Configuration: Lin
Total energy [Ha]: -4027.48752968195
Number of imaginary frequencies: 2 (-30.74 and -11.29 cm-1)
Multiplicity: 1
Charge: +1

Ni	3.4329470969	-0.8963847940	-0.3710149487
C	5.6203654864	-0.7953869780	-0.9783382099
O	5.1883086364	-1.5607841713	-0.0563516526
O	4.7258907334	-0.0970703524	-1.5642357887
C	7.0658476935	-0.6993823935	-1.3251340307
H	7.5890063992	-1.6172158406	-1.0271182486
H	7.5032377546	0.1487856237	-0.7725998789

H	7.1832958988	-0.5036515815	-2.3996830768
C	-0.5539173475	-0.7541114981	2.2212020263
C	-0.1724808044	-2.1367821892	2.1770088583
C	1.1801599033	-1.7199901021	1.8867476262
C	0.8586770579	-0.3244493208	2.2846573412
S	1.6995112023	1.0287933624	2.5974283899
S	2.4914422592	-2.4159381750	1.0939746148
N	-1.6901322569	-0.1253282504	1.9100514865
N	-0.8590739871	-3.2762647562	2.1990968169
C	-1.6691899346	1.1181949862	1.0815747404
H	-2.5024138131	-0.7211717930	1.7642825376
C	-0.6781215401	0.8914642547	-0.0679307822
C	0.3644692447	1.8455649550	-0.3070335961
C	1.6399387910	1.3283752664	-0.7223702074
N	1.8063854119	-0.0174022983	-0.9538779149
C	0.7293639408	-0.7811260502	-1.0804011601
C	-0.5412401148	-0.3572375105	-0.6512742808
C	-3.1241200285	1.5331352644	0.7812921237
H	-1.2409419865	1.9116830865	1.7071102883
N	-3.1841283502	2.8305771962	0.0987735819
C	-4.0124086654	0.4819289444	0.0543791126
H	-3.5552011768	1.7039202134	1.7801842022
C	-4.9847137822	1.2634524737	-0.8586973660
C	-3.0056689889	2.7021894575	-1.3610244847
C	-4.1532853890	1.8695455428	-2.0019545359
H	-2.0297700241	2.2435514059	-1.5645077425
H	-2.9577543149	3.7187134284	-1.7790797737
H	-3.7471251887	1.0716161167	-2.6448849158
H	-4.7952197043	2.4975291995	-2.6394028787
C	-5.6582757976	2.4218949814	-0.0789442053
H	-5.7438201551	0.5812041597	-1.2680463725
C	-4.5171164933	3.4056731106	0.3548327520
H	-4.5645418983	-0.1392174320	0.7796839508
H	-3.4164340650	-0.2035797807	-0.5722245388
H	-4.5953990991	3.6453544059	1.4274969406
H	-4.5827751388	4.3591194276	-0.1897055578
C	-0.4166339246	-4.5304350087	1.5602517531
H	-1.8537691929	-3.2043910663	2.3915018736
C	-0.8126641762	-4.5562838094	0.0958942192
H	-0.8797851484	-5.3601892164	2.1145537753
H	0.6715990513	-4.6113492366	1.6804308268
C	0.1487104826	-4.4208140900	-0.9144291032
C	-0.2388875594	-4.3605466994	-2.2574285889
C	-1.5920326099	-4.4275186012	-2.6014712274
C	-2.5578720952	-4.5757390542	-1.5992038197

C	-2.1686239794	-4.6450067806	-0.2593664953
H	-2.9301697359	-4.7821503142	0.5157537854
H	1.2045485916	-4.3523876967	-0.6444158670
H	0.5204105154	-4.2649086954	-3.0377356551
H	-1.8951181007	-4.3795850808	-3.6500929793
H	-3.6151465431	-4.6513007428	-1.8648974645
C	-6.5342638878	1.9792023306	1.1041545633
H	-6.3198189600	2.9389647796	-0.7952974816
C	-7.6936711724	1.0578945053	0.7234175283
H	-6.9342244880	2.8891321841	1.5850354506
H	-5.9098883248	1.4947053654	1.8771045902
H	-8.3157206934	1.5124462522	-0.0651198373
H	-8.3448306150	0.8575208557	1.5882979855
H	-7.3399982011	0.0853652889	0.3454614987
H	0.8844884363	-1.8252766305	-1.3534647969
H	-1.3191898087	-1.1186079909	-0.5710824323
C	2.7398158664	2.2146996825	-0.8019235975
C	2.5840302833	3.5562776091	-0.5222231357
C	1.3169514311	4.0757173328	-0.1296337615
C	0.2290571416	3.2160785957	-0.0146296340
H	-0.7394874750	3.6222526684	0.2844073382
H	3.7049788466	1.8251425497	-1.1237834389
H	3.4455049800	4.2169828636	-0.6161197008
O	1.0927222994	5.3695460322	0.1460150035
C	2.1508813963	6.3134373722	0.0792047323
H	1.7162012426	7.2807916041	0.3604212803
H	2.5644873609	6.3884929447	-0.9415127733
H	2.9610907382	6.0635463144	0.7858561321

System: Acetate-Ni-L
Ligand: Thiosquaramide
Configuration: Lin
Total energy [Ha]: -4027.50151669786
Number of imaginary frequencies: 1 (-47.49 cm-1)
Multiplicity: 3
Charge: +1

Ni	3.4768394037	-0.6638702527	-0.1330086175
C	5.7667490227	-0.0818046316	0.0865594548
O	5.3169963410	-1.1659814140	0.5658103469
O	4.9470341621	0.6754529159	-0.5304864592
C	7.2009726448	0.3161039086	0.2590735140
H	7.8038136343	-0.5386406596	0.5894192285
H	7.2542422962	1.1117545421	1.0200212554
H	7.5902693417	0.7319581306	-0.6810340613

C	-0.3565508397	-0.9857885019	1.8285758382
C	-0.1715681740	-2.4175234873	1.7258231266
C	1.2022563261	-2.1806588799	1.3145248492
C	1.0790232535	-0.7724019562	1.7091745250
S	2.2150718555	0.4298832988	1.7239766760
S	2.3897682464	-2.9042610642	0.4029460712
N	-1.4311687582	-0.2117229879	1.6655585967
N	-0.9743402988	-3.4596277635	1.8087687012
C	-1.3930211086	1.0837021754	0.9224786002
H	-2.3274392203	-0.6913318357	1.6485490570
C	-0.4845819759	0.9357901387	-0.3019704331
C	0.4841716443	1.9526107229	-0.6001849666
C	1.7043499389	1.5258026855	-1.2327297955
N	1.9221964742	0.2099412508	-1.5667617678
C	0.8791430272	-0.6049039546	-1.5642782247
C	-0.3527461740	-0.2775499510	-0.9558057192
C	-2.8569027508	1.5559803485	0.7466324284
H	-0.9038483585	1.8191726230	1.5757784483
N	-2.9425998088	2.8904624956	0.1461265181
C	-3.8113363691	0.5631578950	0.0216659422
H	-3.2170565228	1.6778766587	1.7808284485
C	-4.8450914912	1.4126581820	-0.7521939388
C	-2.8835200543	2.8431280568	-1.3290752291
C	-4.1081623056	2.0897950083	-1.9198730678
H	-1.9466630634	2.3611408848	-1.6311888399
H	-2.8268072680	3.8802333030	-1.6913920410
H	-3.7891416211	1.3397549865	-2.6616357651
H	-4.7912530011	2.7808364253	-2.4381547526
C	-5.4296125344	2.5236712274	0.1583478461
H	-5.6483829938	0.7674670079	-1.1360080535
C	-4.2395664449	3.4717334619	0.5405069068
H	-4.3170487241	-0.1035916909	0.7408035840
H	-3.2711099138	-0.0774865652	-0.6962019561
H	-4.2250405654	3.6634615812	1.6257195631
H	-4.3326356049	4.4496730210	0.0459732262
C	-0.6552960946	-4.8118720106	1.3038407726
H	-1.9482975519	-3.2929601982	2.0507548043
C	-1.3338219233	-5.0468678721	-0.0285800846
H	-0.9872477246	-5.5410126387	2.0570886589
H	0.4380313256	-4.8802028864	1.2217106458
C	-0.8050716437	-4.4679503071	-1.1942293497
C	-1.4591493490	-4.6247341728	-2.4174972263
C	-2.6484408841	-5.3609289219	-2.4880001481
C	-3.1767549758	-5.9436038104	-1.3330125957
C	-2.5217769961	-5.7859480143	-0.1066785717

H	-2.9352240151	-6.2519488928	0.7929274119
H	0.1342237626	-3.9103172163	-1.1400292007
H	-1.0347993745	-4.1837139177	-3.3230141359
H	-3.1572843161	-5.4874124712	-3.4465895769
H	-4.0980085703	-6.5285043165	-1.3858524624
C	-6.1949225841	2.0125606222	1.3896310303
H	-6.1498884380	3.0909300698	-0.4563064710
C	-7.3832905620	1.1051370823	1.0687601288
H	-6.5495818848	2.8924757129	1.9542286226
H	-5.5033174010	1.4895984483	2.0757984072
H	-8.0807056027	1.5966033437	0.3705759123
H	-7.9479161823	0.8513351584	1.9793445351
H	-7.0643894890	0.1582326106	0.6043572680
H	1.0505067324	-1.6281479924	-1.9093811063
H	-1.0838385212	-1.0779167363	-0.8255804196
C	2.7402744933	2.4724151260	-1.4198941252
C	2.5722962810	3.7884441086	-1.0434221592
C	1.3583187747	4.2160696063	-0.4337939806
C	0.3385391007	3.2965222972	-0.2049657307
H	-0.5935343555	3.6461715576	0.2429609618
H	3.6780425419	2.1317519871	-1.8555305725
H	3.3837938688	4.4952030856	-1.2153756732
O	1.1191907089	5.4806630363	-0.0490401681
C	2.1110980339	6.4817052180	-0.2147261118
H	1.6797651807	7.4092369670	0.1823339999
H	2.3657177452	6.6302184660	-1.2784557975
H	3.0285476873	6.2411619105	0.3502060327

System: Acetate-Ni-L
Ligand: Thiosquaramide
Configuration: Din
Total energy [Ha]: -4027.52408829244
Number of imaginary frequencies: 1 (-80.02 cm-1)
Multiplicity: 1
Charge: +1

Ni	-1.611196	0.921460	-1.447908
C	-2.997193	-0.296015	-2.797703
O	-3.378510	0.158050	-1.667824
O	-1.793390	-0.020142	-3.108321
C	-3.884997	-1.090850	-3.689844
H	-4.841564	-1.291485	-3.193422
H	-3.375998	-2.031992	-3.943019
H	-4.049019	-0.535414	-4.626404
C	2.055919	0.616859	0.101721

C	3.458628	0.789607	-0.156360
C	3.162069	1.512346	-1.413560
C	1.716528	1.297425	-1.118686
S	0.393419	1.762970	-2.038197
S	4.006356	2.229108	-2.606385
N	1.358051	0.067533	1.105597
N	4.558138	0.439701	0.475217
C	-0.111411	0.013996	1.078086
H	1.839140	-0.510564	1.785634
C	-0.599103	-1.047979	2.045264
C	-1.531098	-2.059612	1.636393
C	-1.934461	-3.015932	2.636931
N	-1.460320	-3.006753	3.909230
C	-0.589349	-2.084676	4.245155
C	-0.132071	-1.084963	3.348100
C	-0.675715	1.474850	1.233113
H	-0.379081	-0.300769	0.060261
N	-1.845032	1.727376	0.320241
C	-1.004455	1.968812	2.658238
H	0.109491	2.119796	0.824578
C	-2.218402	2.909832	2.586728
C	-3.117951	1.236778	0.953190
C	-3.439357	2.049787	2.229042
H	-2.991928	0.167103	1.153413
H	-3.908237	1.316097	0.200611
H	-3.684522	1.368426	3.057734
H	-4.319386	2.690996	2.067511
C	-2.023009	3.969918	1.480667
H	-2.374045	3.396941	3.559160
C	-1.971874	3.211205	0.122448
H	-0.122217	2.468099	3.085324
H	-1.255984	1.133133	3.324001
H	-1.119585	3.543379	-0.485217
H	-2.881181	3.365609	-0.475433
C	5.931834	0.589949	-0.057970
H	4.468817	-0.136992	1.308926
C	6.443557	-0.722593	-0.606437
H	6.563518	0.951569	0.766570
H	5.891160	1.366019	-0.833710
C	6.071013	-1.134486	-1.896233
C	6.494894	-2.371013	-2.386919
C	7.290405	-3.206692	-1.594265
C	7.667660	-2.800453	-0.311163
C	7.244761	-1.561724	0.180624
H	7.551800	-1.241000	1.181115

H	5.458067	-0.475996	-2.518652
H	6.210696	-2.681841	-3.395304
H	7.623127	-4.172730	-1.981743
H	8.297882	-3.445404	0.305926
C	-0.799295	4.882060	1.671092
H	-2.918772	4.613266	1.481264
C	-0.791762	5.662089	2.985520
H	-0.769025	5.591480	0.825650
H	0.128276	4.286994	1.584787
H	-1.718650	6.246826	3.106537
H	0.052235	6.367700	3.021409
H	-0.702123	4.998609	3.860587
H	-0.215094	-2.097992	5.275800
H	0.579550	-0.339266	3.714080
C	-2.869496	-4.024165	2.277371
C	-3.387881	-4.107405	1.004262
C	-2.981295	-3.170899	0.011358
C	-2.070696	-2.174611	0.333772
H	-1.804810	-1.478406	-0.456355
H	-3.160219	-4.734242	3.053614
H	-4.103052	-4.894760	0.767817
O	-3.432368	-3.183176	-1.262970
C	-4.357101	-4.176331	-1.677022
H	-4.566374	-3.980856	-2.736355
H	-5.301968	-4.116051	-1.109647
H	-3.936135	-5.191588	-1.578521

System: Acetate-Ni-L
Ligand: Thiosquaramide
Configuration: Din
Total energy [Ha]: -4027.51884393649
Number of imaginary frequencies: 1 (-36.77 cm-1)
Multiplicity: 3
Charge: +1

Ni	-1.8929985227	0.1008366630	-1.5403635108
C	-3.5005501956	-0.7699537307	-3.0567907583
O	-3.8608167318	-0.2321243015	-1.9566791963
O	-2.2632228710	-0.7844977654	-3.3295553360
C	-4.5169866629	-1.3446534579	-3.9947046580
H	-5.2560405007	-1.9276088407	-3.4275825693
H	-4.0316402584	-1.9613955036	-4.7606975568
H	-5.0570446564	-0.5187991709	-4.4850576846
C	1.9343581258	0.4534384374	0.3447866383
C	3.3300944033	0.7230381773	0.1578497361

C	3.1230024580	0.9024476909	-1.2978040125
C	1.6761837617	0.6162731926	-1.0668452341
S	0.4332572399	0.5119079619	-2.1955411580
S	4.0445901520	1.2423691717	-2.5958423981
N	1.1538503221	0.1612390106	1.3971328772
N	4.3698366208	0.7833072066	0.9629392360
C	-0.2753797412	-0.0187614553	1.1517451817
H	1.5368454822	0.0839729125	2.3316515360
C	-0.9423768938	-1.0251993373	2.0512156930
C	-1.7751968478	-2.0325415261	1.4575747808
C	-2.5540148621	-2.8352889894	2.3597352555
N	-2.4580967132	-2.7392796443	3.7110094836
C	-1.6200565693	-1.8681612055	4.2218986717
C	-0.8537905590	-0.9741749119	3.4291324129
C	-0.9624401964	1.3710170543	0.9866547184
H	-0.2983246889	-0.4471125983	0.1515858460
N	-2.1676831084	1.2870562419	0.1044337420
C	-1.2888689081	2.1916195061	2.2503242772
H	-0.2405530608	1.9343223650	0.3808947566
C	-2.5656078751	3.0114995477	1.9714578950
C	-3.4015037320	0.9205274695	0.8734750702
C	-3.7468123069	2.0304993965	1.8959367422
H	-3.2146630081	-0.0385464788	1.3637402671
H	-4.1950982291	0.7494414658	0.1371812055
H	-3.9416593771	1.5871246892	2.8844511391
H	-4.6621139037	2.5665307581	1.6010710194
C	-2.4607962297	3.7430238239	0.6119503325
H	-2.7300997251	3.7358144783	2.7808690312
C	-2.3879565126	2.6448999182	-0.4913713209
H	-0.4369317189	2.8381609477	2.5074916634
H	-1.4749957295	1.5410160813	3.1171613951
H	-1.5598525293	2.8442432322	-1.1897504364
H	-3.3149388523	2.5840733357	-1.0798741774
C	5.7760917205	0.9486432685	0.5310750190
H	4.2354123732	0.5260242639	1.9383359541
C	6.5157935819	-0.3690110855	0.5715824250
H	6.2395325507	1.6868834662	1.2021808999
H	5.7516861444	1.3661063469	-0.4842667618
C	6.3618950148	-1.2869944360	-0.4795812860
C	7.0075120434	-2.5239892814	-0.4311041684
C	7.8089157627	-2.8549783295	0.6681547486
C	7.9674830990	-1.9443011859	1.7165983483
C	7.3218641874	-0.7047568052	1.6683639798
H	7.4581723320	0.0115932250	2.4844260905
H	5.7448977580	-1.0193101739	-1.3422951656

H	6.8929316244	-3.2299201950	-1.2572178580
H	8.3170698959	-3.8216886046	0.7030245441
H	8.6010415012	-2.1956822476	2.5705910898
C	-1.3063166403	4.7529305372	0.5007263625
H	-3.4002112485	4.3041338425	0.4740025388
C	-1.3548909370	5.8802537642	1.5312427528
H	-1.3339063217	5.1864138344	-0.5142668946
H	-0.3364536493	4.2272978701	0.5659303011
H	-2.3218343932	6.4085245413	1.4956430060
H	-0.5640611048	6.6214631285	1.3405770093
H	-1.2167984460	5.5086292587	2.5589746687
H	-1.5445582250	-1.8252376714	5.3147938796
H	-0.2244215664	-0.2341003691	3.9309435250
C	-3.4738732827	-3.7786876484	1.8207150795
C	-3.6186182803	-3.9523138666	0.4639915238
C	-2.8134216691	-3.1973775935	-0.4365034667
C	-1.9093826246	-2.2586805311	0.0578787479
H	-1.2000982285	-1.8515909590	-0.6742700193
H	-4.0614170875	-4.3621800048	2.5319612701
H	-4.3338994309	-4.6815394717	0.0852226199
O	-2.8547104633	-3.3431615396	-1.7704337521
C	-3.6301038401	-4.3754125012	-2.3640938619
H	-3.4341268710	-4.3178353503	-3.4416065410
H	-4.7083714103	-4.2255829970	-2.1848126073
H	-3.3292864527	-5.3691917910	-1.9923640648

6.7.4. Structures of complexes with two ligands

System: Ni-L_2
Ligand: Squaramide
Configuration: O-Ni-O
Total energy [Ha]: -4798.33804984169
Number of imaginary frequencies: -
Multiplicity: 1
Charge: +2

C	3.491179	1.862352	0.313287
C	2.564536	3.008614	0.277820
C	1.447719	2.078671	0.399094
C	2.334283	0.971201	0.355958
O	1.933793	-0.224569	0.391122
O	0.192540	1.941849	0.450322
N	4.786423	1.696329	0.251922
N	2.691680	4.312484	0.214200
C	5.418265	0.343830	0.230356

H	5.401183	2.504695	0.183352
C	6.827001	0.465504	-0.312793
C	7.322586	-0.505043	-1.246654
C	8.631761	-0.265092	-1.797635
N	9.404462	0.794455	-1.444430
C	8.943606	1.637325	-0.551899
C	7.657625	1.515042	0.039101
C	5.202184	-0.332345	1.615418
H	4.823798	-0.248890	-0.477384
N	5.492910	-1.767510	1.577693
C	5.854818	0.344962	2.843669
H	4.111286	-0.285773	1.751704
C	6.138460	-0.780797	3.865886
C	6.913828	-2.060609	1.863429
C	7.298293	-1.624740	3.306245
H	7.539677	-1.555761	1.119019
H	7.065520	-3.139083	1.709393
H	8.229986	-1.037228	3.300951
H	7.476970	-2.495889	3.956342
C	4.903574	-1.707516	4.019096
H	6.418792	-0.349254	4.836912
C	4.670599	-2.394292	2.627588
H	5.191831	1.120461	3.260508
H	6.803827	0.838294	2.581198
H	3.610555	-2.324261	2.330196
H	4.923688	-3.464271	2.661480
C	1.522897	5.225784	0.088638
H	3.614087	4.711453	0.054523
C	1.088246	5.390062	-1.348931
H	1.822276	6.182437	0.538552
H	0.717940	4.800255	0.704950
C	0.146671	4.506593	-1.900605
C	-0.216477	4.621451	-3.244919
C	0.357821	5.615844	-4.044219
C	1.291979	6.501587	-3.497203
C	1.656174	6.389304	-2.152819
H	2.376806	7.093246	-1.725399
H	-0.312999	3.738494	-1.271661
H	-0.959391	3.941158	-3.668338
H	0.069632	5.707927	-5.094037
H	1.731355	7.286581	-4.116860
C	3.636252	-1.016230	4.548329
H	5.178457	-2.482329	4.755185
C	3.786397	-0.364565	5.922951
H	2.833886	-1.774821	4.590084

H	3.286195	-0.263594	3.816674
H	4.164444	-1.087314	6.664324
H	2.821079	0.015241	6.293241
H	4.487673	0.484937	5.905093
H	9.593132	2.472842	-0.265022
H	7.359232	2.271126	0.773079
C	9.148410	-1.187381	-2.747236
C	8.438390	-2.306723	-3.120442
C	7.161579	-2.565215	-2.543178
C	6.620448	-1.673003	-1.624685
H	5.676922	-1.947864	-1.153637
H	10.136485	-0.979582	-3.161510
H	8.867933	-2.995313	-3.847772
O	6.420113	-3.655558	-2.824702
C	6.914821	-4.643064	-3.714598
H	6.149499	-5.428648	-3.758953
H	7.859072	-5.083950	-3.350552
H	7.071164	-4.237148	-4.729251
C	-3.488161	-1.867952	0.299805
C	-2.560502	-3.013035	0.254076
C	-1.444661	-2.083432	0.385159
C	-2.331883	-0.976227	0.350160
O	-1.931978	0.219505	0.393394
O	-0.189552	-1.946272	0.437613
N	-4.783555	-1.703035	0.238713
N	-2.686016	-4.316318	0.176219
C	-5.417604	-0.351486	0.224107
H	-5.396960	-2.511793	0.162968
C	-6.826186	-0.473129	-0.319491
C	-7.323635	0.500783	-1.248836
C	-8.632288	0.260803	-1.801023
N	-9.402949	-0.801857	-1.452729
C	-8.940567	-1.647847	-0.563942
C	-7.654841	-1.525829	0.027674
C	-5.202735	0.318639	1.612335
H	-4.824283	0.245830	-0.480768
N	-5.495031	1.753621	1.580731
C	-5.854941	-0.364748	2.837430
H	-4.111847	0.272835	1.748740
C	-6.139581	0.756360	3.864550
C	-6.916268	2.043907	1.867604
C	-7.300164	1.601726	3.308624
H	-7.541469	1.541265	1.121180
H	-7.069351	3.122794	1.717906
H	-8.231468	1.013631	3.301075

H	-7.479319	2.470019	3.962410
C	-4.905520	1.683505	4.021773
H	-6.419510	0.320380	4.833705
C	-4.673403	2.376749	2.633344
H	-5.191338	-1.141597	3.250731
H	-6.803555	-0.857617	2.572675
H	-3.613291	2.309400	2.335634
H	-4.927811	3.446257	2.672060
C	-1.515830	-5.226151	0.038015
H	-3.607775	-4.714671	0.011226
C	-1.084218	-5.373213	-1.402335
H	-1.812455	-6.188332	0.477843
H	-0.710286	-4.805911	0.657194
C	-0.147214	-4.480191	-1.946421
C	0.212860	-4.578343	-3.292847
C	-0.359739	-5.565765	-4.101937
C	-1.289028	-6.461227	-3.562540
C	-1.650303	-6.365508	-2.216045
H	-2.367232	-7.077013	-1.794925
H	0.311537	-3.717744	-1.309981
H	0.952132	-3.890468	-3.710386
H	-0.073972	-5.644783	-5.153486
H	-1.726991	-7.240894	-4.189879
C	-3.637518	0.991009	4.547850
H	-5.181011	2.454744	4.761384
C	-3.787091	0.332295	5.919163
H	-2.836075	1.750345	4.593578
H	-3.286276	0.242456	3.812576
H	-4.166395	1.050724	6.664080
H	-2.821294	-0.047925	6.287792
H	-4.487047	-0.518198	5.896907
H	-9.588541	-2.485870	-0.280884
H	-7.355024	-2.284599	0.758286
C	-9.150670	1.186413	-2.746446
C	-8.442866	2.308917	-3.114338
C	-7.166635	2.567293	-2.535740
C	-6.623807	1.671917	-1.621333
H	-5.680860	1.946522	-1.148957
H	-10.138293	0.978594	-3.161781
H	-8.873737	3.000045	-3.838468
O	-6.427364	3.660439	-2.812122
C	-6.923744	4.650787	-3.697926
H	-6.159762	5.437852	-3.739000
H	-7.868736	5.088597	-3.332081
H	-7.079388	4.248789	-4.714236

Ni	0.001076	-0.002296	0.440114

System: Ni-L_2
Ligand: Squaramide
Configuration: O-Ni-O
Total energy [Ha]: -4798.37277787706
Number of imaginary frequencies: -
Multiplicity: 3
Charge: +2

C	3.547323	1.693049	0.559108
C	2.654456	2.713173	1.134794
C	1.512680	1.854224	0.856461
C	2.363959	0.915491	0.184644
O	1.956104	-0.121614	-0.397502
O	0.260807	1.754029	0.941907
N	4.847750	1.620147	0.431435
N	2.829921	3.884168	1.699723
C	5.649591	0.514112	-0.173397
H	5.405360	2.399340	0.778862
C	7.113865	0.817876	0.085515
C	8.094784	0.585794	-0.935885
C	9.449208	0.975611	-0.631788
N	9.821214	1.505365	0.562340
C	8.908292	1.667823	1.489296
C	7.539777	1.342526	1.294907
C	5.092416	-0.859710	0.305305
H	5.472739	0.555445	-1.257009
N	5.734605	-1.990272	-0.370034
C	5.026530	-1.083045	1.839199
H	4.065431	-0.879692	-0.078253
C	5.225597	-2.596148	2.076415
C	6.977922	-2.421828	0.302322
C	6.687654	-2.935005	1.741602
H	7.682792	-1.584659	0.319873
H	7.439703	-3.199080	-0.323661
H	7.365025	-2.462474	2.470404
H	6.844422	-4.022262	1.819860
C	4.310621	-3.419098	1.132446
H	5.014892	-2.844150	3.126786
C	4.777614	-3.110592	-0.335196
H	4.061033	-0.737775	2.245922
H	5.816437	-0.530333	2.370931
H	3.915023	-2.858526	-0.974257
H	5.265987	-3.985276	-0.789207

C	1.701445	4.765583	2.097172
H	3.758197	4.302133	1.689353
C	1.290687	5.695871	0.979632
H	2.026710	5.319883	2.988214
H	0.874222	4.105636	2.394365
C	0.486881	5.214961	-0.067187
C	0.127285	6.057343	-1.120369
C	0.566984	7.386477	-1.136332
C	1.365167	7.871001	-0.096210
C	1.727526	7.027375	0.958797
H	2.342892	7.414980	1.776243
H	0.122692	4.184536	-0.046947
H	-0.507359	5.681991	-1.926863
H	0.280502	8.047008	-1.957990
H	1.702464	8.909984	-0.101880
C	2.803325	-3.181109	1.332170
H	4.503234	-4.483484	1.351191
C	2.296868	-3.428179	2.752181
H	2.262591	-3.844577	0.633107
H	2.534144	-2.156917	1.021019
H	2.516039	-4.455515	3.085561
H	1.208398	-3.277838	2.806736
H	2.756576	-2.738151	3.476944
H	9.235289	2.078138	2.452229
H	6.846577	1.507074	2.124942
C	10.450327	0.780676	-1.620804
C	10.154321	0.210143	-2.838317
C	8.821978	-0.207142	-3.125726
C	7.816877	-0.019307	-2.182536
H	6.829913	-0.416444	-2.418363
H	11.467106	1.090412	-1.372819
H	10.948527	0.069695	-3.571176
O	8.456916	-0.798365	-4.278136
C	9.421599	-1.062615	-5.285547
H	8.883562	-1.560505	-6.102174
H	10.218853	-1.732665	-4.919736
H	9.874744	-0.132304	-5.669546
C	-3.662500	-1.569766	0.857440
C	-2.790498	-2.424665	1.681649
C	-1.637204	-1.657024	1.235599
C	-2.464991	-0.905316	0.337683
O	-2.036107	-0.037328	-0.464199
O	-0.387114	-1.537446	1.328266
N	-4.958740	-1.521931	0.685343
N	-2.984927	-3.425738	2.506885

C	-5.732750	-0.584112	-0.183402
H	-5.534453	-2.190545	1.195705
C	-7.206339	-0.825685	0.083946
C	-8.147892	-0.838748	-0.999248
C	-9.511718	-1.165828	-0.664544
N	-9.929064	-1.412066	0.604268
C	-9.053279	-1.345737	1.577840
C	-7.678854	-1.060314	1.364847
C	-5.184611	0.864702	-0.014394
H	-5.516262	-0.872177	-1.221494
N	-5.820363	1.811482	-0.934401
C	-5.139298	1.428757	1.430362
H	-4.152244	0.801841	-0.380249
C	-5.329509	2.957224	1.315035
C	-7.067722	2.384970	-0.389258
C	-6.788030	3.215353	0.895444
H	-7.776399	1.574952	-0.189216
H	-7.520351	2.999960	-1.180764
H	-7.475726	2.927115	1.706502
H	-6.937579	4.292029	0.718325
C	-4.402500	3.541634	0.217687
H	-5.128821	3.436184	2.284027
C	-4.861748	2.911356	-1.145974
H	-4.183746	1.175884	1.919359
H	-5.943127	1.017760	2.061189
H	-3.996400	2.521832	-1.707681
H	-5.346635	3.662217	-1.786861
C	-1.870068	-4.157800	3.162135
H	-3.910815	-3.844892	2.566497
C	-1.411564	-5.344872	2.348302
H	-2.227735	-4.461820	4.155530
H	-1.057468	-3.431318	3.304200
C	-0.572811	-5.149873	1.237968
C	-0.168878	-6.237844	0.462454
C	-0.596716	-7.529878	0.790957
C	-1.427023	-7.730506	1.897281
C	-1.834700	-6.640886	2.673328
H	-2.475808	-6.806276	3.544233
H	-0.216396	-4.145806	0.992567
H	0.490192	-6.081220	-0.394964
H	-0.276531	-8.381907	0.186866
H	-1.754896	-8.738560	2.161220
C	-2.899162	3.347032	0.475045
H	-4.590138	4.628782	0.188837
C	-2.393691	3.924923	1.796191

H	-2.350859	3.818107	-0.361052
H	-2.638476	2.276169	0.419137
H	-2.575689	5.009874	1.858650
H	-1.312511	3.753497	1.902736
H	-2.882785	3.454824	2.663597
H	-9.416913	-1.527344	2.596295
H	-7.015703	-1.026747	2.234262
C	-10.473196	-1.219316	-1.709005
C	-10.132676	-0.938037	-3.012972
C	-8.792860	-0.576093	-3.337969
C	-7.824523	-0.531089	-2.340292
H	-6.831329	-0.184218	-2.623573
H	-11.496844	-1.480828	-1.435143
H	-10.898595	-0.983817	-3.786885
O	-8.385965	-0.254480	-4.579747
C	-9.310912	-0.237602	-5.656095
H	-8.740281	0.055912	-6.546334
H	-10.117276	0.497266	-5.488276
H	-9.755253	-1.233291	-5.827836
Ni	-0.045371	-0.033795	-0.014412

System: Ni-L_2
Ligand: Thiosquaramide
Configuration: S-Ni-S
Total energy [Ha]: -6090.05780642716
Number of imaginary frequencies: 0
Multiplicity: 1
Charge: +2

C	3.9169598212	2.0603674034	0.1871054172
C	3.0295423767	3.2295160081	0.2114607756
C	1.8823341953	2.3387848384	0.1821952444
C	2.7452172654	1.1983948547	0.1429704088
S	2.2533672458	-0.3981654055	0.0759324723
S	0.2084132605	2.2905192265	0.1852502210
N	5.2092956502	1.8655924028	0.2017592701
N	3.2065177265	4.5274237991	0.2509148082
C	5.8308205232	0.5130469597	0.1838802056
H	5.8365749102	2.6670180405	0.2181713007
C	7.2264699540	0.6161888753	-0.3968447039
C	7.6915316076	-0.3691227376	-1.3302308309
C	8.9901569205	-0.1477669688	-1.9132120613
N	9.7801494614	0.9087258837	-1.5900380322
C	9.3472672314	1.7660224528	-0.6972316416
C	8.0743234575	1.6616967102	-0.0759996822

C	5.6492128089	-0.1374686654	1.5871512451
H	5.2137786399	-0.0871588318	-0.4969189958
N	5.9298875195	-1.5747760621	1.5627702312
C	6.3400042065	0.5539544432	2.7859311939
H	4.5639516563	-0.0732139160	1.7536043796
C	6.6386712815	-0.5584992945	3.8186059362
C	7.3564363553	-1.8704954906	1.8160616185
C	7.7777484787	-1.4206855478	3.2441992013
H	7.9646764851	-1.3750184814	1.0511768227
H	7.5010782920	-2.9509646238	1.6692740304
H	8.7151225047	-0.8431897685	3.2105801960
H	7.9619463036	-2.2856009582	3.9010969530
C	5.4008376755	-1.4731754522	4.0160811466
H	6.9447172798	-0.1144147728	4.7762175929
C	5.1331635002	-2.1831434106	2.6429976922
H	5.6934226437	1.3413911873	3.2062718519
H	7.2871967775	1.0338819326	2.4929169611
H	4.0660215051	-2.1180026375	2.3716176350
H	5.3863298861	-3.2527076174	2.6882535193
C	2.1124204798	5.5255000077	0.1497451076
H	4.1555627584	4.8905280181	0.1857006605
C	1.9967057169	6.0704101708	-1.2543923961
H	2.3343877587	6.3199045237	0.8766955709
H	1.1887730703	5.0238569546	0.4701435126
C	1.2954900291	5.3443585356	-2.2304649157
C	1.2256419158	5.8194964957	-3.5412637845
C	1.8575777743	7.0201154218	-3.8862374008
C	2.5544432994	7.7482518387	-2.9174491094
C	2.6248825097	7.2740115603	-1.6040723932
H	3.1603679425	7.8532154498	-0.8456498521
H	0.7903360695	4.4122762395	-1.9588727093
H	0.6705538144	5.2573982334	-4.2959820608
H	1.7990969931	7.3928762920	-4.9115626909
H	3.0387126789	8.6908575913	-3.1823569703
C	4.1490626145	-0.7645645551	4.5595922083
H	5.6870864097	-2.2364654740	4.7599503768
C	4.3382835184	-0.0730162760	5.9094869523
H	3.3487076742	-1.5210313183	4.6469846527
H	3.7765439147	-0.0351355265	3.8160498882
H	4.7417647411	-0.7722787016	6.6600639162
H	3.3828744232	0.3141283999	6.2972712931
H	5.0349510761	0.7782236191	5.8456816550
H	10.0103239806	2.5991187221	-0.4352970679
H	7.8002490489	2.4296439216	0.6552798518
C	9.4771134442	-1.0855679166	-2.8629238017

C	8.7487373682	-2.2024566302	-3.2068391891
C	7.4824475678	-2.4420264859	-2.5990823022
C	6.9700408344	-1.5344502686	-1.6791410304
H	6.0321575923	-1.7940894912	-1.1882588887
H	10.4576038125	-0.8919550603	-3.3013693182
H	9.1562725204	-2.9032578178	-3.9351469054
O	6.7251518852	-3.5281721266	-2.8529550943
C	7.1882489750	-4.5246978241	-3.7495694183
H	6.4124244463	-5.3007626587	-3.7747439709
H	8.1346841326	-4.9757908239	-3.4041188763
H	7.3277138660	-4.1241404501	-4.7687766635
C	-3.9187416844	-2.0535407130	0.2111076224
C	-3.0317655835	-3.2226892801	0.2485960177
C	-1.8841794751	-2.3327597436	0.2080694422
C	-2.7467633598	-1.1926003044	0.1574976556
S	-2.2547887924	0.4032149713	0.0750576744
S	-0.2102164241	-2.2848776333	0.2090209387
N	-5.2109047034	-1.8574932362	0.2243876877
N	-3.2095446168	-4.5198284116	0.3051159600
C	-5.8302987047	-0.5041355169	0.1983920152
H	-5.8391142616	-2.6579336008	0.2493865885
C	-7.2261719479	-0.6071640476	-0.3818377977
C	-7.6884303091	0.3746089232	-1.3203170848
C	-8.9877099494	0.1541234898	-1.9021182936
N	-9.7807732145	-0.8983886395	-1.5735271248
C	-9.3502878417	-1.7523962406	-0.6764263968
C	-8.0770250534	-1.6485745941	-0.0557215900
C	-5.6469368328	0.1532310699	1.5982307512
H	-5.2121292630	0.0910041678	-0.4858132907
N	-5.9280274336	1.5902928252	1.5680784606
C	-6.3360822609	-0.5331762301	2.8008580356
H	-4.5614103258	0.0898216116	1.7634372861
C	-6.6345878586	0.5835067362	3.8289494764
C	-7.3544650739	1.8867217608	1.8213896795
C	-7.7746486447	1.4424556511	3.2516354207
H	-7.9633044223	1.3882089617	1.0589383932
H	-7.4993323989	2.9665953814	1.6705022936
H	-8.7115437483	0.8640094733	3.2208710790
H	-7.9592337849	2.3099042113	3.9050693504
C	-5.3972728616	1.4999642528	4.0215307909
H	-6.9396817946	0.1433606802	4.7886841807
C	-5.1305307400	2.2035245075	2.6449742703
H	-5.6886415094	-1.3182928453	3.2242589620
H	-7.2831839902	-1.0150680117	2.5107661282
H	-4.0635600699	2.1370960342	2.3731606690

H	-5.3836505367	3.2732866103	2.6854130515
C	-2.1162795528	-5.5204352584	0.2214291953
H	-4.1588033489	-4.8831806782	0.2445520911
C	-2.0016371888	-6.0909070837	-1.1725367946
H	-2.3387286829	-6.3014167744	0.9626920350
H	-1.1920387389	-5.0139121570	0.5323468114
C	-1.3012518750	-5.3826541750	-2.1621578468
C	-1.2326797323	-5.8811890853	-3.4642900236
C	-1.8649316806	-7.0878810936	-3.7868212739
C	-2.5606540281	-7.7985473184	-2.8043462415
C	-2.6298502659	-7.3008091034	-1.4996293569
H	-3.1643105619	-7.8662740021	-0.7302068983
H	-0.7961033134	-4.4457554559	-1.9075763525
H	-0.6785022703	-5.3327071413	-4.2296123496
H	-1.8075513406	-7.4791373119	-4.8052995224
H	-3.0450221347	-8.7458324024	-3.0518527668
C	-4.1447196535	0.7950699686	4.5680435008
H	-5.6838357044	2.2666841749	4.7617360191
C	-4.3324167457	0.1114638182	5.9221878825
H	-3.3446203580	1.5524028326	4.6502144666
H	-3.7726506589	0.0614536737	3.8284296895
H	-4.7357318435	0.8150074424	6.6688449439
H	-3.3764700769	-0.2727675657	6.3115319204
H	-5.0286291080	-0.7405526812	5.8640506238
H	-10.0157514323	-2.5822006651	-0.4101775884
H	-7.8054128679	-2.4136852908	0.6794543402
C	-9.4718888139	1.0885219112	-2.8565986139
C	-8.7401406363	2.2014135462	-3.2063046094
C	-7.4730852156	2.4402418445	-2.5998651425
C	-6.9633570635	1.5358260669	-1.6753484424
H	-6.0246217979	1.7949794103	-1.1858628770
H	-10.4529549348	0.8955782942	-3.2940492811
H	-9.1455568989	2.8996499577	-3.9382493542
O	-6.7123965751	3.5226857965	-2.8594026039
C	-7.1726785224	4.5162477816	-3.7607237004
H	-6.3946667845	5.2899934729	-3.7895680851
H	-8.1178499047	4.9716202364	-3.4174361028
H	-7.3132667594	4.1112955742	-4.7780442104
Ni	-0.0007750964	0.0025369835	0.1259379935

System: Ni-L_2
Ligand: Thiosquaramide
Configuration: S-Ni-S
Total energy [Ha]: -6090.07120097306
Number of imaginary frequencies: 0

Multiplicity: 3
Charge: +2

C	-4.301333	1.420792	-0.697451
C	-3.695767	2.476017	-1.514569
C	-2.380212	1.919523	-1.243484
C	-2.973904	0.893362	-0.416149
S	-2.232448	-0.329777	0.450298
S	-0.761468	2.169656	-1.592042
N	-5.519564	1.087121	-0.360369
N	-4.162386	3.492864	-2.196987
C	-5.838257	-0.080010	0.508231
H	-6.300856	1.656155	-0.679932
C	-7.148226	0.180730	1.222865
C	-7.274595	-0.111044	2.621875
C	-8.519206	0.254135	3.249486
N	-9.555956	0.814907	2.574894
C	-9.424385	1.031190	1.288115
C	-8.235824	0.733744	0.570345
C	-5.681314	-1.387340	-0.324760
H	-5.037546	-0.119020	1.255800
N	-5.564687	-2.564716	0.539769
C	-6.684534	-1.632838	-1.476737
H	-4.684715	-1.275736	-0.775888
C	-6.810561	-3.167563	-1.628450
C	-6.885178	-3.141612	0.868189
C	-7.589990	-3.686673	-0.406111
H	-7.494541	-2.373108	1.356990
H	-6.726682	-3.930928	1.617518
H	-8.637971	-3.349990	-0.447375
H	-7.608584	-4.787896	-0.415419
C	-5.411362	-3.837914	-1.621505
H	-7.350478	-3.412752	-2.554071
C	-4.789376	-3.571284	-0.205280
H	-6.336714	-1.154944	-2.406687
H	-7.679680	-1.218355	-1.251131
H	-3.748631	-3.217330	-0.296816
H	-4.763706	-4.490889	0.397854
C	-3.325939	4.530334	-2.849794
H	-5.159047	3.694954	-2.140885
C	-3.248911	5.777181	-2.000266
H	-3.775128	4.736649	-3.831887
H	-2.334853	4.086713	-3.016241
C	-2.410294	5.800195	-0.874075
C	-2.363308	6.930620	-0.057088

C	-3.154339	8.045947	-0.358857
C	-3.987640	8.029972	-1.480801
C	-4.037185	6.896968	-2.299363
H	-4.683449	6.892920	-3.182343
H	-1.780843	4.934306	-0.646521
H	-1.703283	6.947906	0.813352
H	-3.114922	8.931967	0.279011
H	-4.598187	8.902829	-1.722986
C	-4.476204	-3.405373	-2.763293
H	-5.579152	-4.922576	-1.736380
C	-5.016676	-3.673133	-4.167908
H	-3.518389	-3.941118	-2.632647
H	-4.223167	-2.333195	-2.660706
H	-5.289654	-4.733942	-4.292084
H	-4.267481	-3.430786	-4.938049
H	-5.916090	-3.075301	-4.385082
H	-10.280445	1.467839	0.760053
H	-8.216814	0.950495	-0.502893
C	-8.675748	0.006667	4.639919
C	-7.679000	-0.589633	5.379532
C	-6.461879	-0.983032	4.750171
C	-6.273226	-0.745227	3.393452
H	-5.355694	-1.121591	2.939794
H	-9.622356	0.299386	5.097560
H	-7.836066	-0.767752	6.443104
O	-5.450545	-1.597286	5.394826
C	-5.571724	-1.921271	6.770502
H	-4.638061	-2.425666	7.050695
H	-6.417731	-2.605894	6.955308
H	-5.691790	-1.018438	7.394386
C	4.061194	-1.166377	-1.031458
C	3.335656	-2.089366	-1.908074
C	2.087277	-1.441077	-1.545200
C	2.800955	-0.529223	-0.678393
S	2.207674	0.696241	0.293457
S	0.439699	-1.544578	-1.823499
N	5.312067	-1.012252	-0.687789
N	3.685391	-3.090392	-2.678361
C	5.790214	-0.005855	0.297973
H	6.014050	-1.632091	-1.086532
C	7.034904	-0.544974	0.975938
C	7.202845	-0.397947	2.393523
C	8.370539	-1.011369	2.974471
N	9.304998	-1.673417	2.244339
C	9.143522	-1.755713	0.945772

C	8.020945	-1.209970	0.268692
C	5.855192	1.387010	-0.395140
H	4.995271	0.076678	1.048849
N	6.047822	2.467534	0.575431
C	6.815997	1.530518	-1.601541
H	4.828466	1.526692	-0.768794
C	7.307565	2.995649	-1.604953
C	7.475718	2.705969	0.872643
C	8.245678	3.171112	-0.396852
H	7.912247	1.790543	1.286208
H	7.522067	3.456606	1.675360
H	9.164288	2.579128	-0.537178
H	8.555712	4.224649	-0.314901
C	6.117639	3.975373	-1.433745
H	7.850850	3.208565	-2.536822
C	5.490881	3.691889	-0.022868
H	6.304233	1.270576	-2.542087
H	7.690920	0.866463	-1.515554
H	4.396215	3.581006	-0.097940
H	5.679919	4.522964	0.672491
C	2.737085	-4.006193	-3.359631
H	4.661657	-3.380689	-2.677953
C	2.588514	-5.297381	-2.588903
H	3.125891	-4.180697	-4.373098
H	1.781667	-3.471487	-3.449435
C	1.800649	-5.325445	-1.426582
C	1.690571	-6.499991	-0.681126
C	2.367004	-7.655522	-1.091310
C	3.149624	-7.634096	-2.249081
C	3.262316	-6.456994	-2.996179
H	3.868227	-6.448344	-3.907176
H	1.260898	-4.426293	-1.113842
H	1.070499	-6.519646	0.218194
H	2.278276	-8.575903	-0.509419
H	3.670943	-8.536868	-2.575314
C	5.067523	3.924777	-2.556416
H	6.543051	4.993395	-1.435409
C	5.609019	4.229905	-3.952906
H	4.274727	4.651633	-2.304259
H	4.566713	2.938621	-2.558101
H	6.143081	5.194107	-3.968099
H	4.796394	4.289225	-4.694045
H	6.314159	3.458149	-4.300368
H	9.919251	-2.277780	0.373104
H	7.974366	-1.332801	-0.818544

C	8.565336	-0.906510	4.377865
C	7.681348	-0.215575	5.176002
C	6.545963	0.421161	4.596040
C	6.319614	0.326101	3.227403
H	5.480853	0.890036	2.819721
H	9.451398	-1.385997	4.797721
H	7.867078	-0.150200	6.247853
O	5.651396	1.141834	5.300445
C	5.830153	1.338895	6.693733
H	4.997714	1.973719	7.023424
H	6.781488	1.853328	6.914887
H	5.794540	0.385539	7.249353
Ni	-0.058464	0.260548	-0.340826

System: Ni-L_2-acetate
Ligand: Thiosquaramide
Configuration: S-Ni-S
Total energy [Ha]: -6318.66873823024
Number of imaginary frequencies: -
Multiplicity: 1
Charge: +1

C 3.6714055816 2.0679309224 -1.0346313872
C 3.1793337403 3.4303869815 -0.9198371480
C 1.9837293173 2.9345963547 -0.2521253980
C 2.4056656115 1.5736775613 -0.5234307126
S 1.5707271040 0.1798732226 -0.1644261609
S 0.6658469749 3.3924829085 0.6465626206
N 4.8351229799 1.5111569458 -1.3286179869
N 3.6392563405 4.6259329938 -1.2389369267
C 5.1936634100 0.1260564394 -0.9283428181
H 5.5879009081 2.1074099720 -1.6573367164
C 6.5425994498 -0.2461502191 -1.5055362298
C 6.7928726749 -1.5794998672 -1.9752291979
C 8.1048308332 -1.8505083026 -2.5043019133
N 9.0901590291 -0.9153954774 -2.5714892582
C 8.8353481123 0.2879992114 -2.1191836840
C 7.5807448996 0.6654272663 -1.5717842310
C 5.0433177225 0.0168894462 0.6210550786
H 4.4239081274 -0.5333965100 -1.3506217973
N 5.1085822250 -1.3590924467 1.1283660652
C 5.8918850223 0.9746913376 1.4873905658
H 4.0022750827 0.3001429807 0.7841634330
C 6.0133799307 0.3016391328 2.8738180518
C 6.4815321972 -1.7395499745 1.5105326507

C 6.9771209601 -0.8908006007 2.7163896521
H 7.1354221620 -1.6078736273 0.6395416960
H 6.4841147362 -2.8160191813 1.7404644229
H 8.0065503235 -0.5363541148 2.5474984444
H 6.9899518939 -1.4837839092 3.6447604814
C 4.6366473941 -0.2491629038 3.3342567866
H 6.4040110296 1.0166479267 3.6117975328
C 4.2568854832 -1.3940883213 2.3359110020
H 5.4059938325 1.9605956377 1.5589812182
H 6.9012479978 1.1344281505 1.0743001762
H 3.2052159600 -1.2970445734 2.0242555027
H 4.3663448563 -2.3882571394 2.7963420358
C 2.8363629646 5.8635563486 -1.1236063984
H 4.4893713962 4.6826501696 -1.7935036914
C 2.0871225025 6.1693378091 -2.4018521961
H 3.5279867175 6.6764339912 -0.8600281308
H 2.1462786123 5.7221390844 -0.2801207703
C 0.8585363980 5.5415767008 -2.6595274915
C 0.1874524115 5.7766052821 -3.8612747890
C 0.7369984883 6.6416739428 -4.8141737577
C 1.9575515158 7.2746520998 -4.5609534503
C 2.6309986370 7.0380207081 -3.3586864233
H 3.5816049510 7.5429296155 -3.1598978881
H 0.4212526960 4.8758891774 -1.9104564328
H -0.7719541560 5.2893316525 -4.0511941622
H 0.2091615839 6.8281277359 -5.7526242486
H 2.3839496951 7.9589634353 -5.2983579616
C 3.5193107888 0.7997655083 3.4719587090
H 4.7913170141 -0.6880482378 4.3353540717
C 3.8408726629 1.9694977922 4.4001529208
H 2.6078041227 0.3015325907 3.8399839697
H 3.2226622010 1.1910537976 2.4816748533
H 4.1738313153 1.6110879702 5.3892002440
H 2.9398328874 2.5810179623 4.5475605204
H 4.6416126411 2.6126567492 3.9974145083
H 9.6448577927 1.0264220783 -2.1701079269
H 7.4755629062 1.6869492417 -1.1948662629
C 8.3954079871 -3.1609924444 -2.9670644413
C 7.4586966583 -4.1690841636 -2.9051421388
C 6.1674686758 -3.9029718356 -2.3702571425
C 5.8476616940 -2.6307411681 -1.9159999509
H 4.8634694765 -2.4849374983 -1.4735873548
H 9.3948402795 -3.3403039242 -3.3671744032
H 7.7179789221 -5.1648466407 -3.2643782850
O 5.1951813212 -4.8401637381 -2.2701280364

C 5.4596216342 -6.1861672230 -2.6205280984
H 4.5392676870 -6.7448127879 -2.4045390123
H 6.2837190482 -6.6097302350 -2.0204034785
H 5.7000092675 -6.2910749148 -3.6930932957
C -3.1862074359 -2.0619276288 0.6245559639
C -2.1645242324 -2.6798304769 1.4245600947
C -1.4098769632 -1.4493754527 1.4330266533
C -2.3410922727 -0.8726320933 0.4296863166
S -2.3634075596 0.4713658055 -0.5040547259
S -0.1416238351 -0.8298923832 2.3189024352
N -4.4205224148 -2.3690406575 0.2689292977
N -1.9793227537 -3.8888072870 1.9579974825
C -5.3564201898 -1.3604891831 -0.2798121958
H -4.8071541357 -3.2587053308 0.5677262931
C -6.5312043687 -2.0331040106 -0.9552404723
C -7.0800114664 -1.4764046236 -2.1602557919
C -8.1863405764 -2.1758472647 -2.7600749918
N -8.7272447266 -3.3071582259 -2.2319017277
C -8.2187731826 -3.7761896346 -1.1183124539
C -7.1235086408 -3.1729148046 -0.4445081100
C -5.6717003915 -0.3327019080 0.8498261871
H -4.7860772116 -0.7968298381 -1.0272416294
N -6.3459316503 0.8752615513 0.3642193035
C -6.3519789642 -0.8744904739 2.1283414080
H -4.6735133858 0.0147568771 1.1411431888
C -7.1144754468 0.3168446772 2.7528895444
C -7.8132162325 0.7407141239 0.3949578858
C -8.3317699947 0.6057966052 1.8556048802
H -8.0981981560 -0.1326336491 -0.2030691543
H -8.2445958500 1.6172617076 -0.1120946422
H -9.0721279637 -0.2069011635 1.9348684641
H -8.8313007962 1.5280609357 2.1931878545
C -6.2213152670 1.5890645425 2.7679837413
H -7.4439464060 0.0674548242 3.7720181634
C -5.9529455932 1.9698977388 1.2697367854
H -5.6030451636 -1.2863824760 2.8233692210
H -7.0697832123 -1.6813975040 1.9051536640
H -4.8856828534 2.1932854258 1.1091611558
H -6.5180856728 2.8687432844 0.9796730477
C -0.6562129335 -4.3357009452 2.4309438985
H -2.6608691348 -4.6070751951 1.7398679459
C 0.3694767511 -4.4333258791 1.3144527652
H -0.8114750304 -5.3098696195 2.9191178348
H -0.3111923901 -3.6349615832 3.2051739714
C 1.6029527154 -3.7788379193 1.4230290609

C 2.5365342837 -3.8366778627 0.3828442906
C 2.2420265180 -4.5562985237 -0.7790187013
C 1.0146968963 -5.2197415079 -0.8929189020
C 0.0852901092 -5.1588937565 0.1473798634
H -0.8728348158 -5.6782289033 0.0446802923
H 1.8282118168 -3.2008594926 2.3215563295
H 3.4855901547 -3.3011576566 0.4719475301
H 2.9751822815 -4.5987021901 -1.5865792329
H 0.7803282509 -5.7871372599 -1.7973708928
C -4.9230580320 1.4649381212 3.5854018278
H -6.8199930732 2.3894061574 3.2369146274
C -5.1332976541 1.0858499947 5.0510235240
H -4.3939364921 2.4319544844 3.5293725317
H -4.2296596034 0.7485185973 3.1099738743
H -5.8282785588 1.7835300790 5.5477224322
H -4.1850760903 1.1095169800 5.6109111848
H -5.5509048769 0.0720092723 5.1601801071
H -8.6756754673 -4.6815223448 -0.6997639732
H -6.7820276132 -3.6366754698 0.4859944046
C -8.7541580558 -1.6541547210 -3.9520853435
C -8.2815934381 -0.4931697484 -4.5244502832
C -7.2046709652 0.2111435301 -3.9163552807
C -6.6186480094 -0.2796200688 -2.7567812585
H -5.8384339864 0.3243832117 -2.2950125105
H -9.5875607693 -2.2043590232 -4.3926709271
H -8.7466409283 -0.1197929640 -5.4366118474
O -6.6996114216 1.3692145454 -4.3989829372
C -7.2809222698 1.9748087951 -5.5361718555
H -6.7253799313 2.9061606388 -5.7090194995
H -8.3458058848 2.2200257691 -5.3728691596
H -7.1937434637 1.3374243231 -6.4345825842
Ni 0.0932377580 1.2069374431 1.3157310913
C -0.5264522501 2.2450104737 3.6924121750
O -1.0815513621 1.9897299773 2.5374236837
O 0.6422780952 1.9880787436 3.9610471137
C -1.4738852550 2.8785396240 4.6937384941
H -2.0570950895 3.6806888366 4.2187811124
H -0.9092617034 3.2641542556 5.5517869361
H -2.1883918633 2.1163491416 5.0416931644

System: Ni-L_2-acetate
Ligand: Thiosquaramide
Configuration: S-Ni-S
Total energy [Ha]: -6318.71150574067
Number of imaginary frequencies: -

Multiplicity: 3
Charge: +1

C 3.4434044916 2.2375117478 -0.3107872815
C 2.8839330659 3.5611967246 -0.1605134962
C 1.6115263600 2.9881595105 0.2636116177
C 2.1572996143 1.6467620593 0.0243456561
S 1.4488673990 0.1562026862 0.1501380799
S 0.1334795583 3.4639789853 0.8303940280
N 4.6459445590 1.7628540812 -0.5966702023
N 3.3516232255 4.7886596957 -0.3194405245
C 5.0150791615 0.3299174329 -0.5226903993
H 5.3836624360 2.4198143491 -0.8283632649
C 6.3588410378 0.1209264724 -1.1919150086
C 6.6030941371 -1.0374255542 -2.0064193892
C 7.9047615164 -1.1466351006 -2.6133715635
N 8.8904668960 -0.2257739552 -2.4392787126
C 8.6435521825 0.8078590500 -1.6724243466
C 7.3963332947 1.0209382391 -1.0281017066
C 4.8818456926 -0.1570052664 0.9529183482
H 4.2461547685 -0.2203781381 -1.0799981995
N 5.0104218566 -1.6153285231 1.0891906811
C 5.7120523624 0.5834191698 2.0272363901
H 3.8290224834 0.0384389999 1.1785163845
C 5.9332456228 -0.4254835847 3.1766433021
C 6.4081309983 -2.0283340388 1.3187361258
C 6.9377913590 -1.4798233853 2.6745447599
H 7.0209598924 -1.6697874319 0.4838091034
H 6.4443707210 -3.1277319424 1.2809526565
H 7.9367837154 -1.0310440670 2.5503398772
H 7.0399675556 -2.2833304937 3.4216688828
C 4.6089752992 -1.1549325117 3.5287697557
H 6.3329064056 0.0883083490 4.0628504865
C 4.2129472072 -1.9991989846 2.2703561316
H 5.1766069500 1.4814584710 2.3711159033
H 6.6937872155 0.9122842992 1.6490415618
H 3.1492191741 -1.8485406142 2.0316630887
H 4.3580433252 -3.0771886566 2.4404285735
C 2.5313562222 6.0111150881 -0.2088159422
H 4.2727252851 4.8997803843 -0.7346585057
C 2.0633307710 6.5002998726 -1.5616368984
H 3.1421412989 6.7732800415 0.2970460945
H 1.6822192934 5.7702381766 0.4453587324
C 0.9917851454 5.8605688704 -2.2053488250
C 0.5785834510 6.2810537848 -3.4707635139

C 1.2307010902 7.3444733563 -4.1059130792
C 2.2958030177 7.9875498380 -3.4694911338
C 2.7106832289 7.5656216559 -2.2021934869
H 3.5382348303 8.0805218964 -1.7040804017
H 0.4701913930 5.0392933963 -1.7052221265
H -0.2617180481 5.7826777023 -3.9605792950
H 0.9036903151 7.6753551935 -5.0947023420
H 2.8027273929 8.8237785007 -3.9572261799
C 3.4556521640 -0.2456808992 3.9839199888
H 4.8389386736 -1.8422364697 4.3616844933
C 3.7528626479 0.6235530701 5.2033390277
H 2.5840667945 -0.8892594162 4.2012519276
H 3.1119212692 0.4009727236 3.1613192433
H 4.0687700337 0.0172242261 6.0690432019
H 2.8563792677 1.1936809103 5.4894148048
H 4.5540220445 1.3529365502 5.0006748413
H 9.4534264331 1.5340387149 -1.5313980256
H 7.2934795879 1.9076774415 -0.3954883284
C 8.1864816267 -2.2776043951 -3.4241124604
C 7.2539239723 -3.2721610644 -3.6192835452
C 5.9777110648 -3.1760803734 -2.9989753779
C 5.6636286635 -2.0757914801 -2.2126753204
H 4.6906573848 -2.0609812427 -1.7249624389
H 9.1766474594 -2.3319515811 -3.8799630958
H 7.5057955615 -4.1285174671 -4.2442282780
O 5.0176455445 -4.1248794470 -3.1240184135
C 5.2862411644 -5.3236214290 -3.8280733733
H 4.3857606766 -5.9447524827 -3.7310317763
H 6.1430496377 -5.8688227015 -3.3950940960
H 5.4776511268 -5.1374083880 -4.8993678962
C -3.2081501080 -2.1081940682 0.3758320078
C -2.2680104430 -2.9449527100 1.0871038789
C -1.5530801296 -1.7660021982 1.5465164806
C -2.3690497737 -0.9458756395 0.6422766052
S -2.2096766871 0.6379981600 0.1902391843
S -0.4030391081 -1.3172454929 2.6470229010
N -4.3771011865 -2.3240489168 -0.2032975165
N -2.0986170538 -4.2513380205 1.2370297269
C -5.3384378763 -1.2720850634 -0.6136700473
H -4.7131708077 -3.2806937598 -0.2528703733
C -6.5518959436 -1.9222599140 -1.2453953030
C -7.2001103703 -1.3042791531 -2.3676321338
C -8.3223016901 -2.0027213310 -2.9403810874
N -8.7993698446 -3.1774992736 -2.4473192623
C -8.2100293943 -3.6950451884 -1.3968121838

C -7.0839049809 -3.1051476991 -0.7635893113
C -5.6077922404 -0.3263036277 0.5976848666
H -4.8350957978 -0.6560786543 -1.3702398297
N -6.5178637624 0.7791007480 0.2841276415
C -5.9794623551 -0.9925746583 1.9431153237
H -4.6416630621 0.1616033043 0.7451342873
C -6.7716122757 0.0666314857 2.7416721228
C -7.9260261649 0.4342141287 0.5565294474
C -8.1562033851 0.1978930821 2.0784789777
H -8.1898176441 -0.4598037103 -0.0213989433
H -8.5550703066 1.2509161733 0.1711634320
H -8.7528563873 -0.7136888436 2.2464755331
H -8.7113108991 1.0328925657 2.5356869708
C -6.0679320056 1.4494211126 2.6642703282
H -6.8808032457 -0.2500781174 3.7892575556
C -6.1371582537 1.9020621296 1.1625052915
H -5.0735981642 -1.3054469274 2.4860703467
H -6.6062846761 -1.8904335339 1.8085308207
H -5.1629908984 2.2965024471 0.8309329585
H -6.8745609635 2.7063884498 1.0192438523
C -0.8294428774 -4.8358084187 1.7264536827
H -2.7112333234 -4.8677736847 0.7118635522
C 0.2674660965 -4.7788051222 0.6801104174
H -1.0551023830 -5.8717511273 2.0189883501
H -0.5345661227 -4.2874615089 2.6313980701
C 1.3768942608 -3.9408087679 0.8532458140
C 2.3583513831 -3.8446632978 -0.1390072514
C 2.2307251223 -4.5827513291 -1.3191941343
C 1.1296277029 -5.4296810172 -1.4962750379
C 0.1566623663 -5.5309590975 -0.4999354133
H -0.6959934473 -6.2031371858 -0.6438507206
H 1.4686614237 -3.3512537263 1.7671698799
H 3.2213069072 -3.1890262453 0.0082272239
H 2.9961781140 -4.4901599980 -2.0915551479
H 1.0308697000 -6.0170794708 -2.4128298310
C -4.6293825426 1.4876832501 3.2120128580
H -6.6708915245 2.1450651935 3.2740657992
C -4.4623809251 1.0328590536 4.6597178215
H -4.2517913643 2.5198503837 3.1128879848
H -3.9572782781 0.8932773693 2.5724081188
H -5.0993400132 1.6170273366 5.3453076819
H -3.4132451494 1.1585722644 4.9674363303
H -4.7256634586 -0.0295871488 4.7893739295
H -8.6200189765 -4.6327289762 -1.0017096132
H -6.6709624703 -3.6074146757 0.1165615514

C -8.9760401836 -1.4327470545 -4.0641816775
C -8.5731982039 -0.2263179112 -4.5942351530
C -7.4848294343 0.4787783986 -4.0076950372
C -6.8168558449 -0.0580495585 -2.9146073255
H -6.0314373669 0.5460763329 -2.4638362354
H -9.8184187373 -1.9837801581 -4.4861511361
H -9.1022331768 0.1833159747 -5.4545742141
O -7.0447724366 1.6788049852 -4.4472951137
C -7.6930679208 2.3186728826 -5.5290054557
H -7.1721535078 3.2732364619 -5.6809277064
H -8.7559668845 2.5236216802 -5.3084222908
H -7.6272838591 1.7254780684 -6.4588556193
Ni -0.3139947386 1.0656413214 1.7365141863
C -0.1323917727 1.9642118733 3.9661041198
O -1.2598916335 1.8643969669 3.3964031664
O 0.9094811307 1.5999963997 3.3377016795
C -0.0267332830 2.4732842520 5.3784983458
H -0.9233842629 3.0422603172 5.6557295413
H 0.8768694580 3.0882732772 5.4918658486
H 0.0688995886 1.6097729436 6.0568248470

System: Ni-L_2-acetate
Ligand: Thiosquaramide
Configuration: N-Ni-S
Total energy [Ha]: -6090.01641890161
Number of imaginary frequencies: 2 (-10.14 and -4.81 cm-1)
Multiplicity: 1
Charge: +2

C 3.1822417868 1.5001385099 1.5844754439
C 2.1580724824 2.2047379423 2.3007897335
C 2.5551230247 1.6289017279 3.6126146419
C 3.6082668429 0.8160771534 2.8597215851
S 4.7104089554 -0.2794070781 3.2770938752
S 2.1267090609 1.7579386329 5.1663158037
N 3.6091530608 1.4795057173 0.3468521037
N 1.0532744173 2.9149614804 1.8379404270
C 4.4085350853 0.3672930607 -0.2224581895
H 3.1873110166 2.1195864071 -0.3243966159
C 3.4641569029 -0.7784558456 -0.5631719705
C 3.8286720236 -2.1286160363 -0.2429479535
C 2.8275969499 -3.1456173034 -0.4499945390
N 1.5969836760 -2.8770932793 -0.9729770957
C 1.3241186946 -1.6386451619 -1.3186041463
C 2.2164015943 -0.5561581481 -1.1174239436

C 5.3291711044 0.9478427745 -1.3241764963
H 5.0707105438 0.0341364784 0.5832315817
N 6.3107481740 -0.0340880861 -1.7897737813
C 4.6260442826 1.6465409481 -2.5198540505
H 5.9113123027 1.7115767108 -0.7848930275
C 5.5366908045 1.4373327187 -3.7534653540
C 5.7774279715 -0.8873261204 -2.8724457721
C 5.4515245334 -0.0493957862 -4.1416630692
H 4.8841222892 -1.4085508140 -2.5101732056
H 6.5259418565 -1.6659922077 -3.0795039669
H 4.4465680847 -0.2906290402 -4.5242247288
H 6.1613779236 -0.2617651132 -4.9559766462
C 7.0126557377 1.7609014698 -3.4034128115
H 5.1929331147 2.0676370507 -4.5853382995
C 7.4639850148 0.7188363474 -2.3226235558
H 4.4716422655 2.7201593289 -2.3196618227
H 3.6390730097 1.2011964369 -2.7318690723
H 7.9775733035 1.2188798865 -1.4853676572
H 8.1748172716 -0.0088265684 -2.7406252270
C 0.2958813797 3.8350598023 2.7665927186
H 1.2021322780 3.3736058624 0.9330831458
C 0.9529671620 5.1859292542 2.8266979241
H 0.2656321977 3.3334115470 3.7420389402
H -0.7230191916 3.8848454186 2.3599364234
C 0.5522414373 6.2037250192 1.9463857361
C 1.2016772244 7.4409978319 1.9587902683
C 2.2554757298 7.6668582533 2.8501805930
C 2.6574290055 6.6569823084 3.7322508057
C 2.0105367373 5.4194442506 3.7208989394
H 2.3134854921 4.6343593566 4.4191795804
H -0.2847584200 6.0367670859 1.2610405575
H 0.8781816024 8.2335743775 1.2801060466
H 2.7587815152 8.6363997926 2.8649584720
H 3.4713065813 6.8388621107 4.4378593367
C 7.2697168062 3.2127150477 -2.9670576787
H 7.5978757833 1.5927023536 -4.3235928109
C 6.8572070372 4.2693059114 -3.9924025623
H 8.3483704235 3.3141186350 -2.7557031829
H 6.7669159291 3.4122611229 -2.0022966405
H 7.3293598984 4.0777548134 -4.9700187627
H 7.1612041889 5.2769104125 -3.6695110230
H 5.7666871233 4.2897289448 -4.1518345957
H 0.3388070448 -1.4450077638 -1.7598794506
H 1.8778179410 0.4474712881 -1.3833012497
C 3.1543400223 -4.4875283692 -0.1146685588

C 4.3945677713 -4.8237723959 0.3821232438
C 5.3931475489 -3.8219587220 0.5559361876
C 5.1021520595 -2.4981507331 0.2406005567
H 5.9126650391 -1.7740026273 0.3172399902
H 2.4068149279 -5.2617543808 -0.2860503396
H 4.6103435870 -5.8655136508 0.6184290599
O 6.6326311605 -4.0704962593 1.0031257999
C 7.0428640360 -5.3983292357 1.3011046544
H 8.0904651787 -5.3302392987 1.6193204743
H 6.9778312531 -6.0502506494 0.4132007799
H 6.4456025962 -5.8330987791 2.1211913414
C -2.4991831316 -1.8085786208 0.5323250247
C -1.4744443516 -2.7621442641 0.9877955903
C -0.6162640909 -1.6436007064 1.3487970460
C -1.6012656809 -0.7201940442 0.8620428629
S -1.4668791692 0.9595604664 0.8412619174
S 0.8477890740 -1.2413648068 2.0641290604
N -3.7078152236 -1.9144986214 0.0494211861
N -1.4057605430 -4.0638880024 1.0269507129
C -4.5670012329 -0.7527427592 -0.3050844567
H -4.1022736512 -2.8404652912 -0.1049228261
C -5.6193106664 -1.2053536452 -1.2972137151
C -5.9514706601 -0.3749682202 -2.4187685041
C -6.9141479427 -0.9026009439 -3.3520840295
N -7.5187165148 -2.1083444906 -3.1950384382
C -7.2216772920 -2.8275562675 -2.1394852122
C -6.2753681940 -2.4165125233 -1.1636132901
C -5.0393954646 -0.0570877309 1.0082714457
H -3.9067209327 -0.0303075496 -0.8015208016
N -5.6720491273 1.2355038270 0.7395321103
C -5.8772966080 -0.9044434224 1.9933926649
H -4.0975879840 0.1951238631 1.5169167316
C -6.7928422763 0.0854004325 2.7494766556
C -7.1231305795 1.0983528836 0.4900285310
C -7.8608497690 0.5799742208 1.7574455243
H -7.2719999419 0.4184105905 -0.3555225996
H -7.5002243547 2.0799896894 0.1677164928
H -8.5524220492 -0.2378943768 1.4997728333
H -8.4648871774 1.3732350925 2.2251459483
C -5.9805584877 1.3184402676 3.2315724015
H -7.2694251362 -0.4154867632 3.6039215425
C -5.4868004939 2.0661846081 1.9437090476
H -5.2253741441 -1.4605602798 2.6857555630
H -6.5067287299 -1.6433942718 1.4730326123
H -4.4189363027 2.3304322055 2.0294750751

H -6.0360666247 3.0070047276 1.7907878363
C -0.2205109327 -4.8656508176 1.4169816200
H -2.2005902115 -4.6059198667 0.6889654961
C -0.1688469135 -6.1205870740 0.5806036915
H -0.3003602489 -5.1033535303 2.4892623980
H 0.6628033119 -4.2286173375 1.2631452553
C 0.0001072007 -6.0240303854 -0.8128419080
C 0.0234103211 -7.1811117958 -1.5941485398
C -0.1195137941 -8.4380939397 -0.9938850542
C -0.2862692032 -8.5371837226 0.3903976764
C -0.3147623577 -7.3808087608 1.1765625032
H -0.4466497265 -7.4634539060 2.2592826994
H 0.1480217003 -5.0421756530 -1.2719772087
H 0.1624255133 -7.1038921086 -2.6751513994
H -0.0978803057 -9.3419817424 -1.6071351836
H -0.3959213224 -9.5166738695 0.8614146880
C -4.8290629281 0.9987198782 4.2007585106
H -6.6851916030 1.9720867348 3.7728519030
C -5.2459368765 0.2577109428 5.4712896078
H -4.3486012933 1.9540890656 4.4784674072
H -4.0425750148 0.4224508081 3.6765882360
H -6.0393416278 0.8037015847 6.0076782096
H -4.3974169448 0.1429987061 6.1640352529
H -5.6320142403 -0.7512455724 5.2536847557
H -7.7366342542 -3.7884808094 -2.0226907798
H -6.1020243627 -3.0823318624 -0.3114695743
C -7.2633533368 -0.1177715880 -4.4838619376
C -6.7204203382 1.1312677939 -4.6865082417
C -5.7919486599 1.6657508543 -3.7468705677
C -5.4208599659 0.9177584639 -2.6350716573
H -4.7656280436 1.3955639606 -1.9069103543
H -7.9890709002 -0.5393098097 -5.1816419351
H -7.0184625883 1.7072101222 -5.5622708086
O -5.2357953681 2.8885104070 -3.8569835072
C -5.6113607891 3.7499540660 -4.9201563023
H -5.0495039007 4.6815087753 -4.7745490512
H -6.6914728806 3.9762423025 -4.8988144942
H -5.3487092669 3.3239686795 -5.9041276015
Ni 0.5866902615 0.9915628269 1.7480891358
